# Therapeutic potential of garlic chive-derived vesicle-like nanoparticles in NLRP3 inflammasome-mediated inflammatory diseases

**DOI:** 10.7150/thno.60265

**Published:** 2021-09-07

**Authors:** Baolong Liu, Xingzhi Li, Han Yu, Xuan Shi, You Zhou, Sophie Alvarez, Michael J. Naldrett, Stephen D. Kachman, Seung-Hyun Ro, Xinghui Sun, Soonkyu Chung, Lili Jing, Jiujiu Yu

**Affiliations:** 1 Department of Nutrition and Health Sciences, University of Nebraska-Lincoln, 230 Filley Hall, Lincoln, NE 68583-0922, USA.; 2 Center for Biotechnology, University of Nebraska-Lincoln, E117 Beadle Center, Lincoln, NE 68588-0665, USA.; 3 Proteomics and Metabolomics Facility, Nebraska Center for Biotechnology, University of Nebraska-Lincoln, N300 Beadle Center, NE 68588-0115, USA.; 4 Department of Statistics, University of Nebraska-Lincoln, 354B Hardin Hall, Lincoln, NE 68583-0963, USA.; 5 Department of Biochemistry, University of Nebraska-Lincoln, N217 Beadle Center, Lincoln, NE 68588-0665, USA.; 6 Department of Biochemistry, University of Nebraska-Lincoln, N158 Beadle Center, Lincoln, NE 68588-0665, USA.; 7 Department of Nutrition, University of Massachusetts Amherst, 211 Chenoweth Laboratory, 100 Holdsworth Way, Amherst, MA 01003, USA.; 8 School of Pharmacy, Shanghai Jiao Tong University, 800 Dongchuan Rd, Shanghai, 200240, China.

**Keywords:** Nanoparticles, vesicles, garlic chive, NLRP3 inflammasome, obesity

## Abstract

Aberrant activation of the nucleotide-binding domain and leucine-rich repeat related (NLR) family, pyrin domain containing 3 (NLRP3) inflammasome drives the development of many complex inflammatory diseases, such as obesity, Alzheimer's disease, and atherosclerosis. However, no medications specifically targeting the NLRP3 inflammasome have become clinically available. Therefore, we aim to identify new inhibitors of the NLRP3 inflammasome in this study.

**Methods:** Vesicle-like nanoparticles (VLNs) were extracted from garlic chives and other *Allium* vegetables and their effects on the NLRP3 inflammasome were evaluated in primary macrophages. After garlic chive-derived VLNs (GC-VLNs) were found to exhibit potent anti-NLRP3 inflammasome activity in cell culture, such function was further assessed in a murine acute liver injury disease model, as well as in diet-induced obesity. Finally, GC-VLNs were subjected to omics analysis to identify the active components with anti-NLRP3 inflammasome function.

**Results:** GC-VLNs are membrane-enclosed nanoparticles containing lipids, proteins, and RNAs. They dose-dependently inhibit pathways downstream of NLRP3 inflammasome activation, including caspase-1 autocleavage, cytokine release, and pyroptotic cell death in primary macrophages. The inhibitory effects of GC-VLNs on the NLRP3 inflammasome are specific, considering their marginal impact on activation of other inflammasomes. Local administration of GC-VLNs in mice alleviates NLRP3 inflammasome-mediated inflammation in chemical-induced acute liver injury. When administered orally or intravenously, GC-VLNs accumulate in specific tissues and suppress activation of the NLRP3 inflammasome and chronic inflammation in diet-induced obese mice. The phospholipid 1,2-dilinoleoyl-sn-glycero-3-phosphocholine (DLPC) in GC-VLNs has been identified to inhibit NLRP3 inflammasome activation.

**Conclusions:** Identification of GC-VLNs and their active component DLPC as potent inflammasome inhibitors provides new therapeutic candidates in the treatment of NLRP3 inflammasome-driven diseases.

## Introduction

The NLRP3 inflammasome protein complex is formed by NLRP3, apoptotic speck protein containing a caspase recruitment domain (ASC) and caspase-1 (Casp1) 1,2. As a vital player in the innate immune system, NLRP3 inflammasome's activation is tightly regulated through a two-step process - it must first be primed and then be assembled 3,4. Priming signals, such as lipopolysaccharide (LPS) or tumor necrosis factor alpha (TNFα), upregulate transcription of the *Nlrp3* and *Il1b* genes through activation of nuclear factor-κB (NF-κB) signaling and induce post-translational modifications of NLRP3 protein. A variety of stimuli from pathogens, hosts, or the environment, such as nigericin, adenosine triphosphate (ATP), alum, serve as activating signals to trigger the assembly and activation of the NLRP3 inflammasome, leading to autocleavage of Casp1. Casp1 cleaves itself to generate Casp1 p10 and p20, which in turn cleave 1) gasdermin D to induce pyroptotic cell death and 2) pro-interleukin (IL)-1β and pro-IL-18 to release mature cytokines IL-1β and IL-18. Genetic evidence indicates that both cytokine release and pyroptosis are essential contributors to the NLRP3 inflammasome-mediated inflammatory process 5. Inappropriate activation of the NLRP3 inflammasome has been implicated in many diseases, such as obesity 6, cryopyrin-associated autoinflammatory syndrome 7, Alzheimer's disease 8, atherosclerosis 9, and gout 10. Therefore, the NLRP3 inflammasome has been considered to be a highly desirable drug target, but therapeutics specifically targeting this protein complex has not become available in clinical practice.

In obesity, the increased levels of circulating LPS from gut microbiota 11,12 and accumulated metabolic stress molecules (such as free fatty acids [FFAs] and cholesterol crystals) 2-4 act as the priming and activating signals, respectively, and lead to assembly and activation of the NLRP3 inflammasome. Reduced expression of the *NLRP3* gene in adipose tissue is correlated with decreased inflammation and improved insulin sensitivity in obese type 2 diabetic patients 6. Genetic deletion of the *Nlrp3*, *Pycard (Asc)*, or *Casp1* genes has been shown to improve glucose homeostasis, accompanied by decreased inflammatory cytokines in the serum and reduced expression of inflammatory genes in adipose tissues in diet-induced obese mice 6,13,14. Fulminant hepatic failure is a rare acute hepatic disorder associated with high mortality 15. Administering D-galactosamine (GalN) and LPS in mice induces acute inflammation and liver injury, simulating many clinical features of fulminant hepatic failure 16. The chemical inhibitor of the NLRP3 inflammasome, MCC950, alleviates inflammation and liver damage in GalN/LPS-challenged mice 17, suggesting critical involvement of the NLRP3 inflammasome in this disease model.

Dietary vesicle-like nanoparticles (VLNs) have been recently identified in many commonly consumed foods, including bovine milk, broccoli, ginger, apple, and honey 18-22. These dietary VLNs are membrane-enclosed nanoparticles (50-300 nm in diameter) containing proteins, lipids, and RNAs. Growing evidence indicates that dietary VLNs are stable, bioavailable, and bioactive in consumers. The membrane of a dietary VLN encloses the biomolecules inside the nanoparticle, thus conferring stability and resistance to solutions in the stomach and intestines and protecting the biomolecules from degradation 23. As a result, dietary VLNs are able to reach target tissues where they exert their bioactivity. For example, it has been found that orally administered ginger VLNs accumulate in the liver, induce expression in the liver of detoxifying/antioxidant genes, including *Hmox1, Nqo1, Gclm,* and *Gclc*, and ameliorate liver damage caused by alcohol consumption in mice 24. Grape-derived VLNs are taken up by intestinal stem cells and promote their proliferation through regulation of the Wnt/β-catenin signaling pathway 25. The bioavailability, stability, and bioactivity of these dietary VLNs confer upon them high therapeutic potential. Therefore, we sought to screen dietary VLNs for anti-NLRP3 inflammasome activities.

## Results

### Characterization of VLNs from garlic chives

Many species in the genus *Allium*, such as garlic, leek, onion, and scallion, are commonly consumed throughout the world. Many of them have anti-inflammatory properties 26. Therefore, in this study, we selected six vegetables from the *Allium* genus for VLN extraction and assessed the effects of their VLNs on the NLRP3 inflammasome. Bulbs of garlic (*A. sativum*) and purple or white onion (*A. cepa*) and leaves of leek (*A. ampeloprasum*), scallion (*A. fistulosum*), and garlic chive (*A. tuberosum*) were minced and subjected to VLN extraction using the protocol established in our laboratory 19,27. VLNs were obtained from all *Allium* vegetables. Nanoparticle tracking analysis (NTA) showed that sizes of *Allium*-derived VLNs ranged from 113 nm to 153 nm in diameter (Figure S1A). The yields of *Allium*-derived VLNs varied from 1.5×10^11^ g^-1^ to 8.9×10^11^ g^-1^ (Figure S1B). To evaluate the effects of *Allium*-derived VLNs on NLRP3 inflammasome activity, these nanoparticles were incubated with murine bone marrow-derived macrophages (BMDMs), followed by NLRP3 inflammasome activation using LPS and ATP. Antibodies against macrophage surface markers F4/80 28 and CD11b 29 were used to validate the macrophage identity of BMDMs. Over 96% of BMDMs were F4/80 and CD11b double positive (Figure S1C), suggesting that the majority of BMDMs were indeed macrophages. Most *Allium*-derived VLNs had marginal or mild inhibitory effects on the level of the Casp1 autocleavage product Casp1 p10 in cell lysates upon activation of the NLRP3 inflammasome, but GC-VLNs strongly inhibited Casp1 autocleavage (Figure S1D). Therefore, GC-VLNs were chosen for further studies.

**Figure 1:**
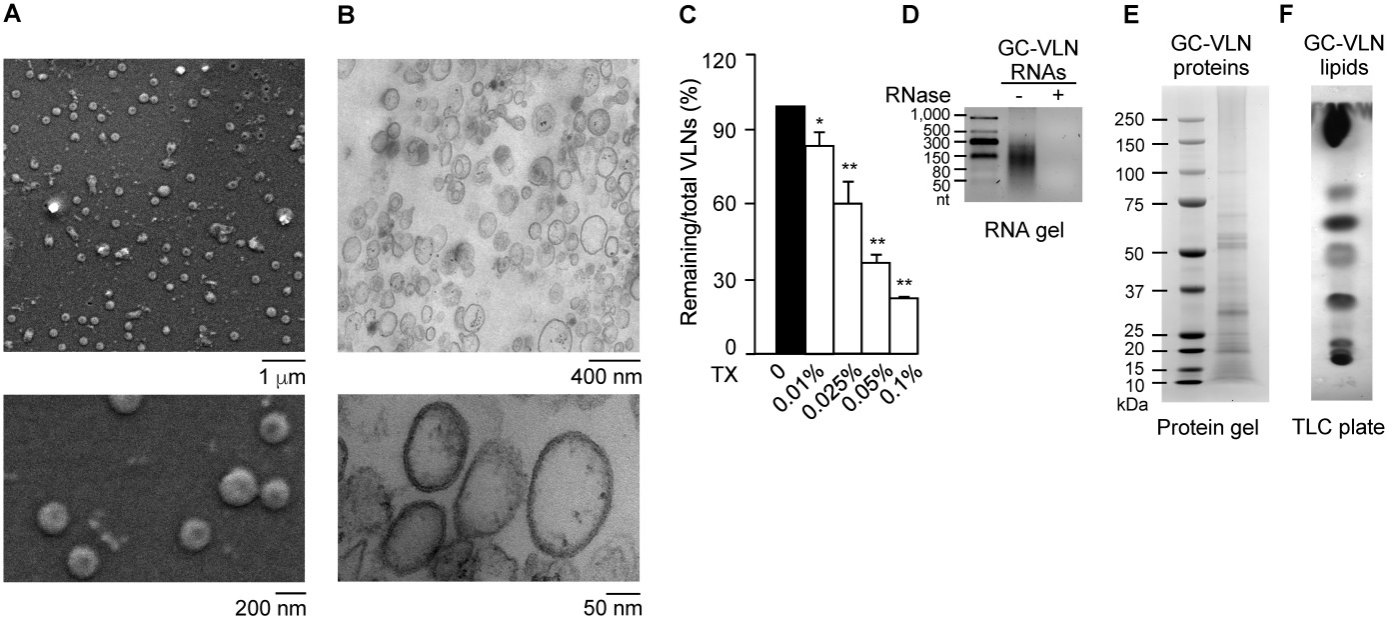
** Characterization of GC-VLNs.** (**A**) Representative SEM images of GC-VLNs. (**B**) Ultrastructure TEM images of GC-VLNs. (**C**) Effects of Triton X-100 treatment on nanoparticle numbers of GC-VLNs. Triton X-100 (TX) at different concentrations was used to lyse GC-VLNs, followed by measurement of particle numbers. Results were expressed as mean±SEM from three independent experiments. * (p < 0.05) and ** (p < 0.01) compared with the sample without Triton X-100 treatment (black bar). (**D**) Agarose gel of RNAs extracted from GC-VLNs. 2.5% agarose gel was used. RNA ladder: 50-1000 nucleotides (nt). (**E**) Coomassie blue staining of protein gel of GC-VLN proteins. 4-12% NuPAGE Bis-Tris protein gel was used. Protein ladder: 10-250 kDa. (**F**) CuSO_4_/phosphoric acid staining of GC-VLN lipids on a thin layer chromatography (TLC) silica gel plate. 10% CuSO_4_ in 8% phosphoric acid was used to visualize the lipid bands.

The vesicle-like features of GC-VLNs were assessed in detail. Scanning electron microscopy (SEM) showed that GC-VLNs were well-dispersed individual ball-like nanoparticles (Figure 1A), and ultrastructure transmission electron microscopy (TEM) demonstrated that GC-VLNs were enclosed by a membrane structure (Figure 1B). Detergent treatment is a simple way to distinguish between protein aggregates and membrane-enclosed particles, because protein aggregates are much more resistant to detergent-based lysis than membrane-bound particles 30. The non-ionic detergent Triton X-100, which disrupts lipid-lipid interaction and lipid-protein interaction 30, was found to lyse GC-VLNs and therefore reduce GC-VLN numbers in a dose-dependent manner (Figure 1C), further confirming the membrane-bound nature of GC-VLNs. Biomolecule analysis revealed that these nanoparticles contained small-sized RNAs, proteins, and lipids (Figure 1D-F).

The proteins in GC-VLNs were subjected to proteomics analysis using the reference proteome database *viridiplantae*, because the proteome of garlic chive was unavailable. A variety of proteins were identified in GC-VLNs (Table S1). A series of plasma membrane proteins, such as plasma membrane ATPase, ABC transporter G family member, pleiotropic drug resistance protein, and hypersensitive-induced response protein, were found in GC-VLNs. Clathrin heavy chain and light chain and Ras-related protein, cytosolic proteins that are known to associate with plasma membrane 31,32, were identified in GC-VLNs. The presence of plasma membrane proteins and cytosolic proteins that interacted with the plasma membrane in GC-VLNs supported their vesicle-like properties.

### GC-VLNs suppressed NLRP3 inflammasome activation

**Figure 2:**
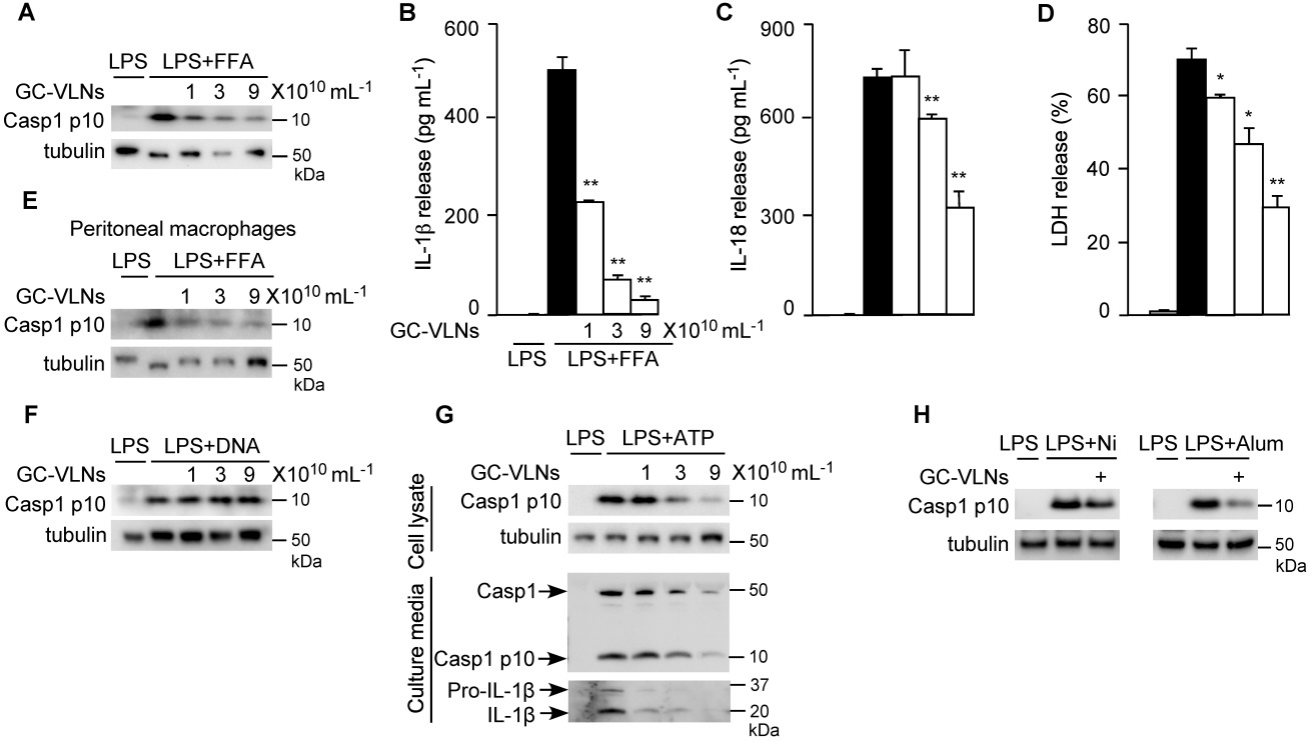
**GC-VLNs specifically inhibited NLRP3 inflammasome activation.** (**A-D**) BMDMs were preincubated with GC-VLNs for 16 h, primed with LPS for 3 h, and stimulated with FFA sodium palmitate for 12 h to activate the NLRP3 inflammasome. Cells were lysed for immunoblot analysis of Casp1 p10 (**A**), and the cell-free media were subjected to enzyme-linked immunosorbent assay (ELISA) analysis to measure the release of IL-1β (**B**) and IL-18 (**C**). Pyroptotic cell death was assessed by measuring the level of lactate dehydrogenase (LDH) released in the culture media (**D**). (**E**) Immunoblot analysis of Casp1 p10 in lysates of peritoneal macrophages preincubated with GC-VLNs for 16 h, followed by NLRP3 inflammasome activation using LPS and FFA. (**F**) Immunoblot analysis of Casp1 p10 in cell lysates of BMDMs preincubated with GC-VLNs for 16 h, followed by AIM2 inflammasome activation. (**G**) Immunoblot analysis of Casp1 and IL-1β in cell lysates and culture media of BMDMs preincubated with GC-VLNs for 16 h, primed with LPS for 3 h, and stimulated with ATP for 30 min to activate the NLRP3 inflammasome. (**H**) Immunoblot analysis of Casp1 p10 in lysates of LPS-primed BMDMs, in which the NLRP3 inflammasome was activated by nigericin (Ni) or alum. 9×10^10^ mL^-1^ of GC-VLNs were used to pretreat the cells for 16 h. Results were expressed as mean±SEM from three independent experiments. * (p < 0.05) and ** (p < 0.01) compared with LPS+FFA group (black bar). Tubulin was included to show equivalent loading in immunoblot analysis. LPS-treated macrophages were used as a negative control for inflammasome activation.

In obesity, increased levels of LPS and FFA often serve as priming and activating signals, respectively, to activate the NLRP3 inflammasome 33. Therefore, to mimic obesity condition *in vitro*, we first pre-treated BMDMs with GC-VLNs, followed by LPS priming and FFA treatment to activate the NLRP3 inflammasome. Remarkably, GC-VLNs suppressed all the downstream events of NLRP3 inflammasome activation in a dose-dependent manner, including Casp1 autocleavage (Figure 2A), IL-1β secretion (Figure 2B), IL-18 secretion (Figure 2C), and pyroptosis (Figure 2D). We were not able to detect the Casp1 autocleavage product Casp1 p10 and its cleaved mature IL-1β in the culture media using immunoblot analysis, possibly because of relative weak activation of the NLRP3 inflammasome by FFA. The levels of Casp1 p10 in cell lysates are generally consistent with its levels in culture media 34-36. Therefore we conducted immunoblot analysis of Casp1 p10 using cell lysates to monitor the Casp1 autocleavage status. Activation of NF-κB signaling via LPS priming leads to release of two potent cytokines IL-6 and TNFα 37. Interestingly, GC-VLNs also suppressed the release of both IL-6 and TNFα (Figure S2A-B). In peritoneal macrophages, GC-VLNs also suppressed Casp1 autocleavage when the NLRP3 inflammasome was activated using LPS and FFA (Figure 2E). The inhibitory effects of GC-VLNs on the NLRP3 inflammasome were specific because GC-VLNs did not inhibit Casp1 autocleavage mediated by the Absent in Melanoma 2 (AIM2) inflammasome (Figure 2F). AIM2 senses cytosolic DNAs during bacterial or viral invasion, recruits ASC and Casp1 to form the AIM2 inflammasome, and leads to Casp1 autocleavage and activation 1,2.

ATP is a commonly used stimulus to activate the NLRP3 inflammasome 38. When the NLRP3 inflammasome was activated by ATP in LPS-primed BMDMs, GC-VLNs dose-dependently decreased the level of Casp1 p10 in the cell lysates and culture media (Figure 2G). IL-1β release was also decreased by GC-VLN treatment, as indicated by IL-1β immunoblot analysis of the media (Figure 2G) and IL-1β ELISA analysis (Figure S3A). Secretion of IL-18 and pyroptotic cell death were consistently alleviated by GC-VLNs (Figure S3B-C). GC-VLNs also blunted Casp1 autocleavage when the NLRP3 inflammasome was activated by ATP in LPS-primed peritoneal macrophages (Figure S3D). A time-course experiment demonstrated that incubation for at least 6 h was needed for GC-VLNs to inhibit NLRP3 inflammasome-mediated Casp1 autocleavage (Figure S3E). In addition, GC-VLNs inhibited NLRP3 inflammasome-mediated Casp1 autocleavage when it was activated by other stimuli, such as nigericin or alum (Figure 2H).

The cellular events targeted by GC-VLNs during NLRP3 inflammasome activation were further investigated. GC-VLNs had marginal effects on the levels of either inflammasome subunits (NLRP3, Casp1, and ASC), pro-IL-1β, or the critical inflammasome modulator never in mitosis gene a (NIMA)-related kinase 7 (Nek7) 35 (Figure S4A). When the sensor NLRP3 is activated, it forms oligomers, recruits the adaptor ASC, and induces ASC oligomerization. The oligomerized ASC further recruits the effector Casp1 to form an oligomerized protein complex with high molecular mass 39. The ASC oligomerization assay showed that GC-VLNs suppressed ASC oligomerization (Figure S4B). The NLRP3 inflammasome complex with high molecular mass can be detected as a single speck in each macrophage cell using ASC immunofluorescence staining 35. GC-VLN treatment reduced the speck formation in BMDMs (Figure S4C-D). Therefore, GC-VLNs seemed to impede assembly and formation of the NLRP3 inflammasome complex.

GC-VLNs were labeled with a lipophilic dye 1,1'-dioctadecyl-3,3,3',3'-tetramethylindocarbocyanine perchlorate (DiI) and incubated with BMDMs. GC-VLNs were taken up by BMDMs in a dose-dependent manner (Figure S5A). In the time-course experiment, DiI-labeled GC-VLNs were detected in BMDMs after 3 h incubation and accumulated in cells over the time (Figure S5B). Proteins inside GC-VLNs were labeled with Exoglow protein dye, and these protein-labeled GC-VLNs accumulated dose-dependently in BMDMs (Figure S5C). When RNAs in GC-VLNs were labeled with Exoglow RNA dye, they were also readily detected in primary macrophages (Figure S5D). Together, these uptake experiments suggested that intact GC-VLNs and their contents (proteins and RNAs) were taken up by BMDMs.

### GC-VLNs reduced inflammation during acute liver injury

Administration of GalN and LPS through intraperitoneal injection triggers acute liver injury and inflammation in mice 16. MCC950, a specific chemical inhibitor of the NLRP3 inflammasome, suppresses inflammation and liver damage in this disease model 17. We confirmed the critical involvement of the NLRP3 inflammasome in this disease model using a mouse strain without expression of the NLRP3 protein (Figure S6A). A mixture of GalN and LPS was injected in *Nlrp3*^-/-^ mice and their wildtype (wt) littermates to induce acute liver injury. The mice were sacrificed after 6 h. The level of serum IL-1β in the *Nlrp3*^-/-^ mice was much lower compared to their wt littermates after GalN/LPS challenge (Figure S6B), suggesting that acute inflammation in this disease model was largely dependent on the NLRP3 inflammasome. Therefore, this disease model was used to assess the anti-NLRP3 inflammasome function of GC-VLNs *in vivo* in our initial proof-of-concept study.

**Figure 3:**
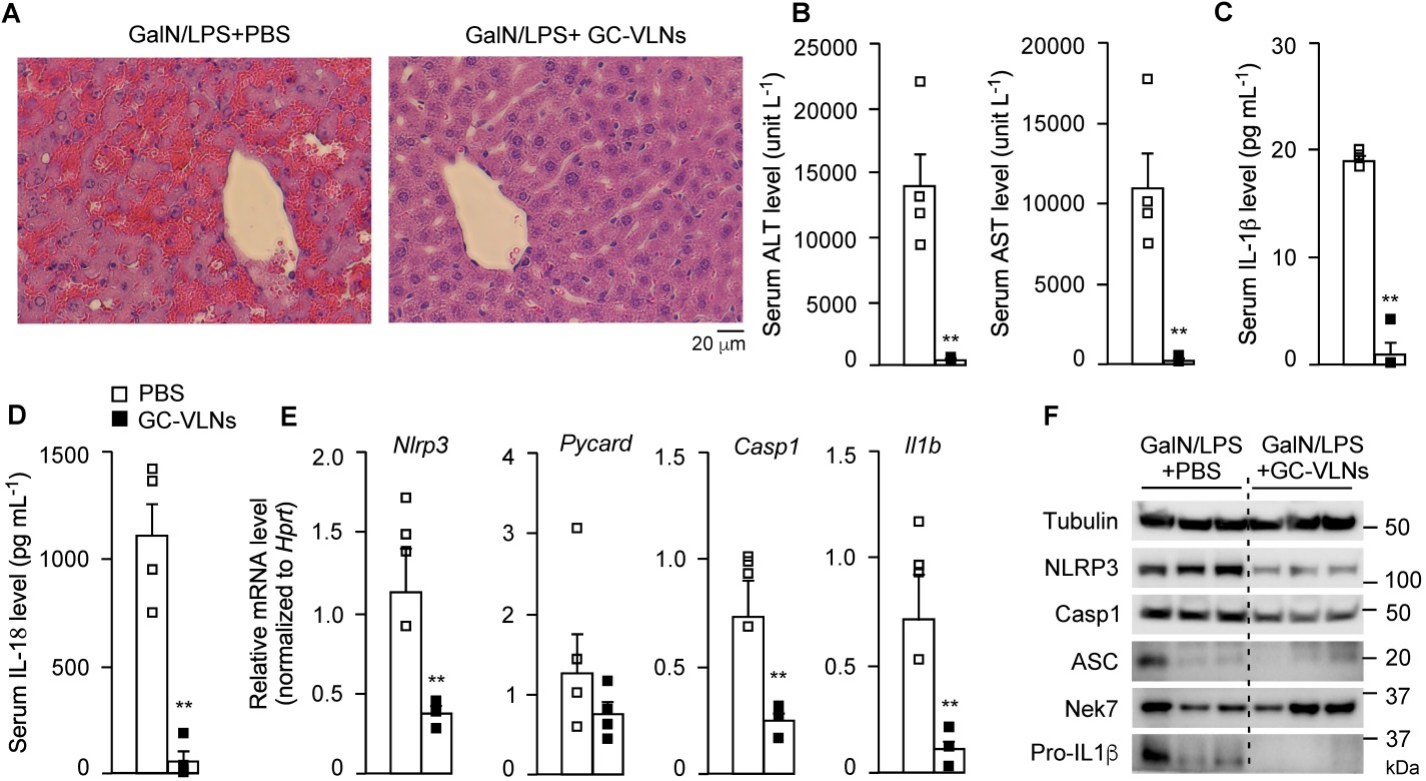
** GC-VLNs alleviated inflammation in chemical-induced acute liver injury in mice.** 8-week-old male C57BL/6J mice were intraperitoneally injected with the solvent phosphate-buffered saline (PBS) or GC-VLNs in PBS at 1×10^10^ g^-1^. 48 h later, acute liver injury was induced by intraperitoneal injection of GalN/LPS mixture. The mice were sacrificed 6 h after GalN/LPS injection. N = 4/group. (**A**) Representative images of liver sections with hematoxylin and eosin (H&E) staining. (**B**) Levels of AST and ALT in serum. (**C**) Levels of IL-1β in serum. (**D**) Levels of IL-18 in serum. (**E**) Relative mRNA levels of *Nlrp3, Pycard, Casp1*, and *Il1b* genes in the livers. The housekeeping gene hypoxanthine guanine phosphoribosyl transferase (*Hprt*) was used to normalize mRNA levels. (**F**) Immunoblot analysis of liver lysates. Data were presented as mean±SEM. * (p < 0.05) and ** (p < 0.01) compared with the control group (bar with white squares).

GC-VLNs were intraperitoneally injected into C57BL/6J mice. The therapeutic agent delivered through intraperitoneal injection would be primarily absorbed into the mesenteric vessels, drain directly into the portal vein, and pass through the liver 40. Therefore, this local injection route was chosen to efficiently deliver GC-VLNs to the liver. Considering the absorption process of GC-VLNs, the minimum 6 h of treatment needed to observe the anti-inflammasome function of GC-VLNs *in vitro*, as well as our preliminary time-course *in vivo* test, we decided to wait for 48 h after GC-VLN administration to inject a mixture of GalN and LPS into mice to induce acute liver injury. GC-VLNs remarkably alleviated severe liver bleeding and cellular damage to hepatocytes caused by the GalN/LPS challenge (Figure 3A). The levels of alanine aminotransferase (ALT) and aspartate aminotransferase (AST) in circulation are often used as indicators of liver injury 41. GC-VLNs significantly lowered the circulation levels of ALT and AST induced by GalN/LPS (Figure 3B). The levels of circulating IL-1β and IL-18, two downstream cytokines of NLRP3 inflammasome activation, were suppressed by GC-VLNs (Figure 3C-D). In the livers, GC-VLNs reduced both mRNA and protein levels of the *Nlrp3, Casp1*, and *Il1b* genes (Figure 3E-F). GC-VLNs had no impact on the protein levels of NLRP3, Casp1, and pro-IL-1β in BMDMs (Figure S4A). The exact reasons for the differential effects of GC-VLNs *in vitro* and *in vivo* were not clear. It has been reported that mature IL-1β is responsible for recruiting macrophages 42. Therefore, it is plausible that GC-VLNs suppressed the NLRP3 inflammasome and thus led to diminished mature IL-1β, which in turn reduced macrophage infiltration and detection of inflammatory gene expression in liver tissue. Considering the inhibitory effects of GC-VLNs on IL-6 and TNFα *in vitro*, the levels of these cytokines in serum during acute liver injury were measured. Surprisingly, the levels of IL-6 and TNFα in circulation were not significantly affected by GC-VLNs (Figure S6C), but the transcription of *Il6* and *Tnf* genes was decreased by GC-VLN treatment (Figure S6D). Together, GC-VLNs reduced acute inflammatory responses and alleviated liver injury in GalN/LPS-challenged mice. The potent anti-inflammatory activities of GC-VLNs in acute liver inflammation prompted us to further investigate their therapeutic potential in chronic inflammation in obesity.

### GC-VLNs were distributed in specific tissues in mice

**Figure 4:**
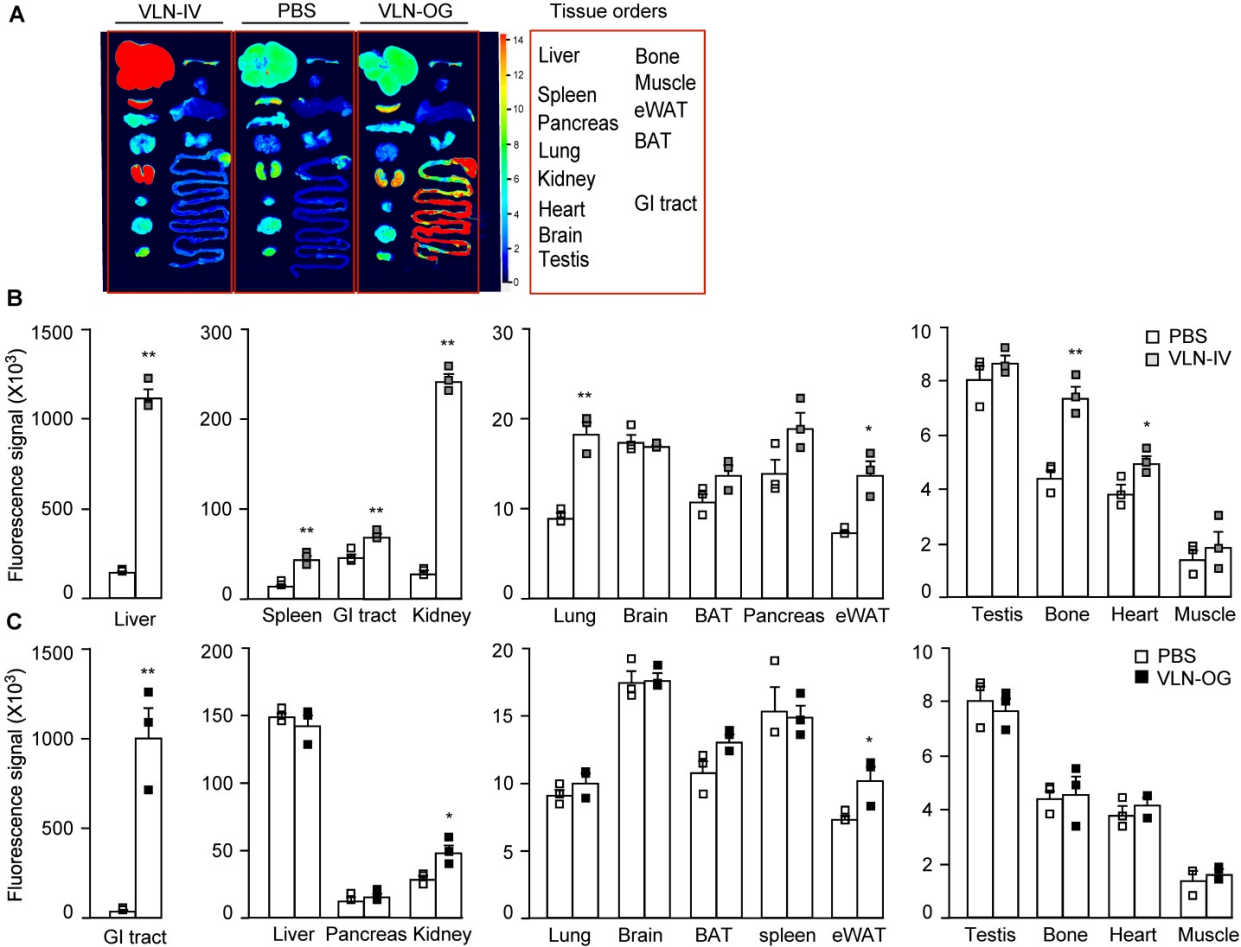
** Distribution of GC-VLNs covalently linked to a fluorescence dye in diet-induced obese mice.** GC-VLNs were covalently labeled with a fluorescence dye in near infrared ranges. The labeled GC-VLNs were intravenously administered at 3,500 FI g^-1^ or orally given at 60,000 FI g^-1^ to male C57BL/6J mice, which were fed with a HFD for 15 weeks. 6 h later, the mice were sacrificed to collect tissues to measure the fluorescence signals in each type of tissue. N = 3/group. (**A**) Representative images of mouse tissues under Licor Odyssey Clx image system. (**B**) Fluorescence signal intensity of mouse tissues collected from control mice received with the solvent PBS and mice intravenously injected with GC-VLNs in PBS (VLN-IV). (**C**) Fluorescence signals of mouse tissues collected from control mice and mice orally administered with GC-VLNs (VLN-OG). Data were presented as mean±SEM. * (p < 0.05) and ** (p < 0.01) compared with the control PBS group (bar with white squares).

The bioavailability of GC-VLNs *in vivo* was evaluated using fluorescence-labeled GC-VLNs. An ExoGlow-Vivo EV labeling kit was used to covalently link fluorescence dye to proteins on the membranes of GC-VLNs, which excluded the possibility of dye dissociation often reported for the lipophilic fluorescence dyes 24. Systemic delivery of therapeutic agents is often achieved through parenteral (outside the digestive tract) and enteral (through digestive tract) administration 40. Intravenous injection is a typical parenteral administration that leads to higher bioavailability, compared to enteral approaches, because it bypasses absorptive processes and hepatic metabolism of the agent. However, enteral approaches (typically oral administration) are less invasive and patient friendly. Therefore, we chose both intravenous and oral administration to assess the biodistribution of GC-VLNs. When GC-VLNs with fluorescence intensity (FI) of 3,500 g^-1^ were injected intravenously in lean healthy mice, the fluorescence signals of the GC-VLNs were detected in the liver, spleen, kidney, lung, brown adipose tissue (BAT), epididymal white adipose tissue (eWAT), and heart; however, intravenous injection of a free dye control with the same 3,500 FI g^-1^ led to no fluorescence signals in any of the examined tissues (Figure S7A-C). When 3,500 FI g^-1^ of GC-VLNs were given intravenously to diet-induced obese C57BL/6J mice, the labeled GC-VLNs accumulated in liver, spleen, kidney, lung, eWAT, heart, bone, and gastrointestinal (GI) tract (Figure 4A-B). It is interesting to note that the distribution patterns of GC-VLNs in lean and obese mice largely overlapped, with only a slight difference.

For oral administration, we found that 60,000 FI g^-1^ (approximately 0.6×10^10^ nanoparticles g^-1^) of GC-VLNs were needed to detect fluorescence signals in the GI tract and kidney in lean mice (Figure S8A-C). Oral administration of free dye with the same fluorescence intensity led to detection of fluorescence signals in the GI tract. We reasoned that possible free dye contamination in 60,000 FI g^-1^ of GC-VLNs should be much lower than 60,000 FI g^-1^. In our labeling procedure, GC-VLNs were washed with PBS twice after labeling. The fluorescence intensity of free dye from the first wash was approximately 3,600/0.6×10^10^ nanoparticles and the fluorescence intensity of free dye from the second wash was around 1,200/0.6×10^10^ nanoparticles. Therefore, we decided to use free dye control at 7,000 FI g^-1^, which should be much higher than any possible contamination from free dye in the final labeled GC-VLNs. Oral administration of this free dye control did not lead to detection of significant fluorescence signal in any tissues (Figure S8A-C). When 60,000 FI g^-1^ of GC-VLNs were orally administered to diet-induced obese C57BL/6J mice, GC-VLNs were found in the GI tract, kidney, and eWAT (Figure 4A and C). The distribution patterns of orally administered GC-VLNs in lean and obese mice were also similar, but GC-VLNs accumulated significantly in eWAT in obese mice.

### GC-VLNs improved metabolic health and reduced inflammation in obesity

**Figure 5:**
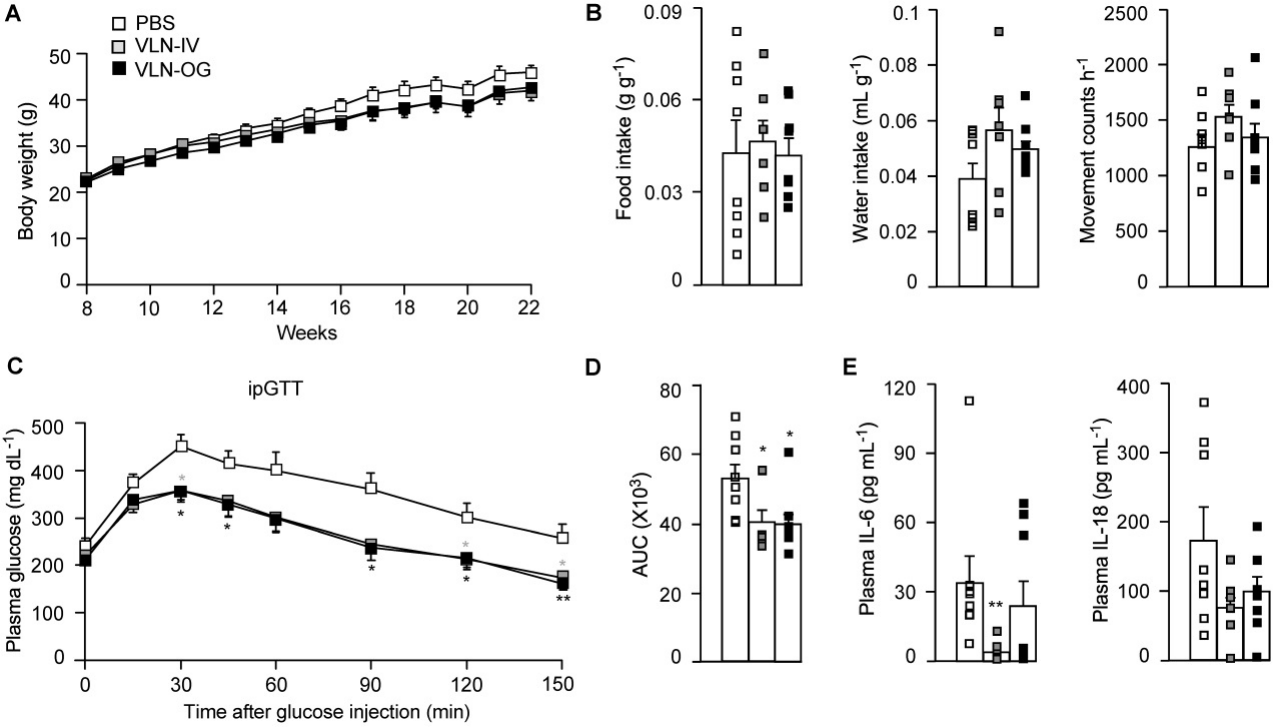
** GC-VLNs improved metabolic health in diet-induced obese mice.** 8-week-old male C57BL/6J mice were fed with a HFD, and GC-VLNs were given through oral gavage (VLN-OG) or intravenous injection (VLN-IV) at the dose of 1×10^10^ g^-1^ weekly when the HFD feeding started. N = 7-8 for each condition. (**A**) Body weight gain of the mice between 8 and 22 weeks of age. (**B**) Food intake, water intake, and movement counts. After 11-week treatment, the mice were individually kept in metabolic cages for 48 h. The data from the final 24 h were used for the calculation. (**C**) Intraperitoneal glucose tolerance test (ipGTT). After 13-week treatment, the mice were intraperitoneally injected with 1 g kg^-1^ of glucose after 6 h of fasting. The blood glucose levels were monitored at the indicated intervals in the next 150 min. (**D**) Incremental area under the curve (AUC) for the ipGTT data in (**C**). (**E**) Levels of IL-6 and IL-18 in mouse plasma. Data were presented as mean±SEM. * (p < 0.05) and ** (p < 0.01) compared with the control PBS group (bar with white squares).

To assess the effects of GC-VLNs on NLRP3 inflammasome-mediated chronic inflammation in obesity, we fed C57BL/6J mice with a high-fat diet (HFD) for 15 weeks to induce obesity and obesity-associated chronic inflammation. When the HFD feeding was started, the mice were randomly separated into three groups: mice in the control group received PBS weekly; mice in VLN-IV group were administered with GC-VLNs weekly through intravenous injection (IV); and mice in VLN-OG group were given GC-VLNs weekly through oral gavage (OG). GC-VLNs had no significant effects on the body weight of mice during the entire experiment (Figure 5A). After 11 weeks of treatment, the mice were subjected to metabolic cage analysis. OG- or IV-administered GC-VLNs had marginal impact on food intake, water intake, and general activity (Figure 5B), indicating that GC-VLNs did not cause any detrimental side effects in mice after 11 weeks of administration. After 13 weeks, mice were subjected to a glucose tolerance test. The mice treated with either IV- or OG-administered GC-VLNs cleared blood glucose more efficiently compared to the control group (Figure 5C-D), indicating improved glucose homeostasis in GC-VLN-treated mice. Upon sacrifice, the level of IL-6 in serum was found to be significantly lower in the VLN-IV group, and the level of serum IL-18 tended to be lower in both VLN-IV and VLG-OG mice, compared to the control mice, but the difference did not reach statistical significance (Figure 5E). Levels of IL-1β and TNFα in the serum were undetectable in all the mice.

**Figure 6:**
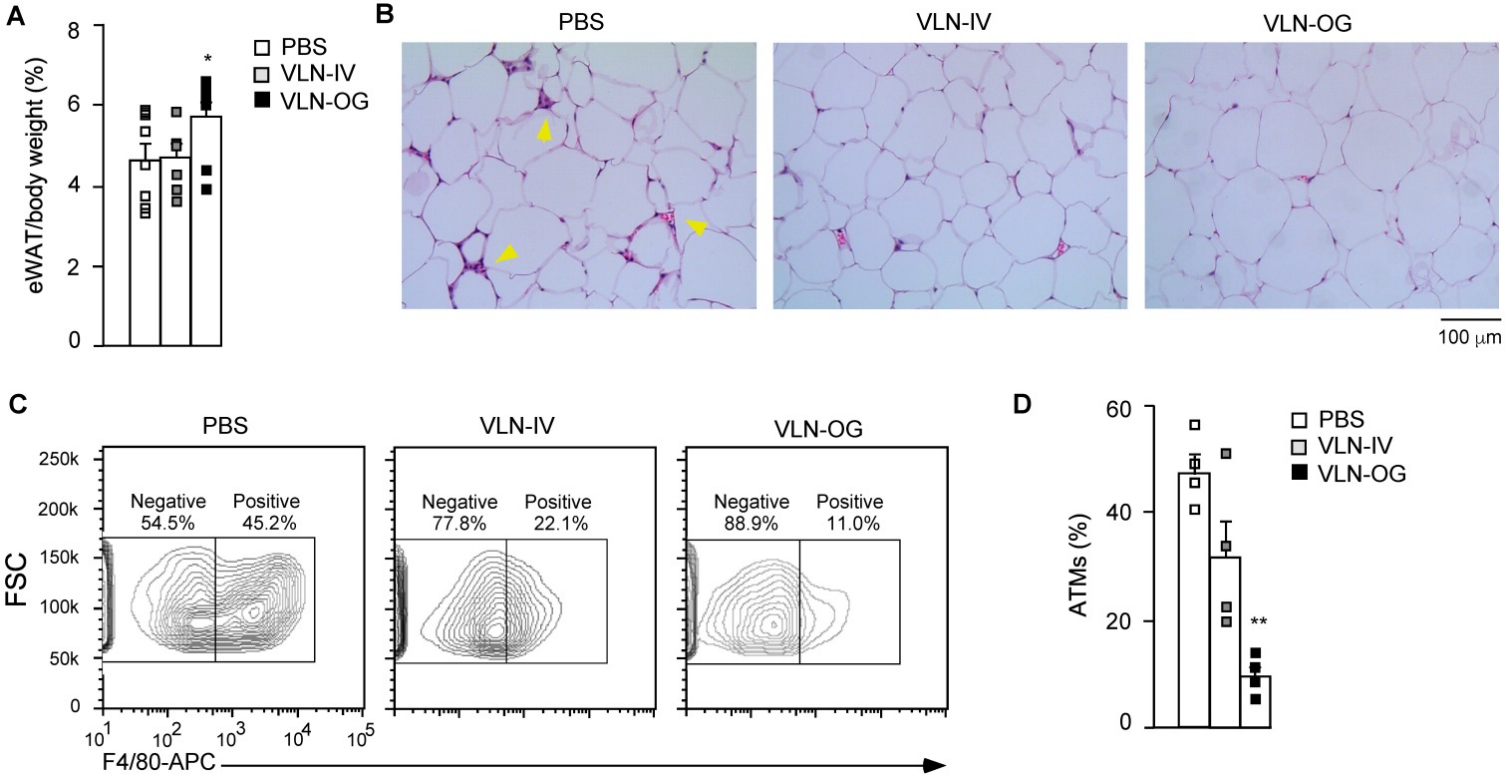
** GC-VLNs reduced immune cell infiltration in eWAT of diet-induced obese mice.** The mice described in Figure 5 were sacrificed after 15-week treatment. eWAT was taken from each mouse and subjected to different analyses. (**A**) Weight percentage of eWAT/body weight. (**B**) Representative images of H&E-stained sections of eWAT. Yellow arrows indicate CLSs in eWAT. (**C**) Representative images of eWAT SVF stained with an anti-F4/80-APC antibody. SVF was extracted from eWAT, stained with the antibody which recognized macrophage surface marker F4/80, and subjected to flow cytometry analysis. To obtain enough cells, eWAT from 2 mice in each group were combined to extract SVF. (**D**) Quantification of adipose tissue macrophages (ATMs) using the data obtained in (**C**). Data were presented as mean±SEM. * (p < 0.05) and ** (p < 0.01) compared with the control group (bar with white squares).

Obesity-associated inflammation and NLRP3 inflammasome activation initially arise in WAT 43,44. Therefore, we mainly focused on the eWAT of these mice. Interestingly, the percentage of eWAT in the VLN-IV group was similar to that in the control group, but the percentage of eWAT in the VLN-OG group was higher than in the control group (Figure 6A). In hematoxylin and eosin (H&E)-stained eWAT slides, crown-like structures (CLSs) were observed in the eWAT in diet-induced obese mice (Figure 6B, left panel). The CLSs, in which macrophages accumulate around dying or dead adipocytes, are a histologic hallmark indicating the proinflammatory state of eWAT 45,46. GC-VLN treatment, especially in the VLN-OG mice, reduced the numbers of CLSs (Figure 6B, middle and right panels). The stromal vascular fraction (SVF) of eWAT, which contains immune cells such as adipose tissue macrophages, adipose-derived stem cells, fibroblasts, and endothelial cells 47,48, was extracted, stained with antibodies against F4/80 (surface marker of macrophages) 28, and subjected to flow cytometry analysis. Orally administered GC-VLNs remarkably reduced macrophage infiltration (Figure 6C-D). Both flow cytometry and histological analysis suggested that GC-VLNs reduced immune cell infiltration in eWAT in diet-induced obese mice.

**Figure 7:**
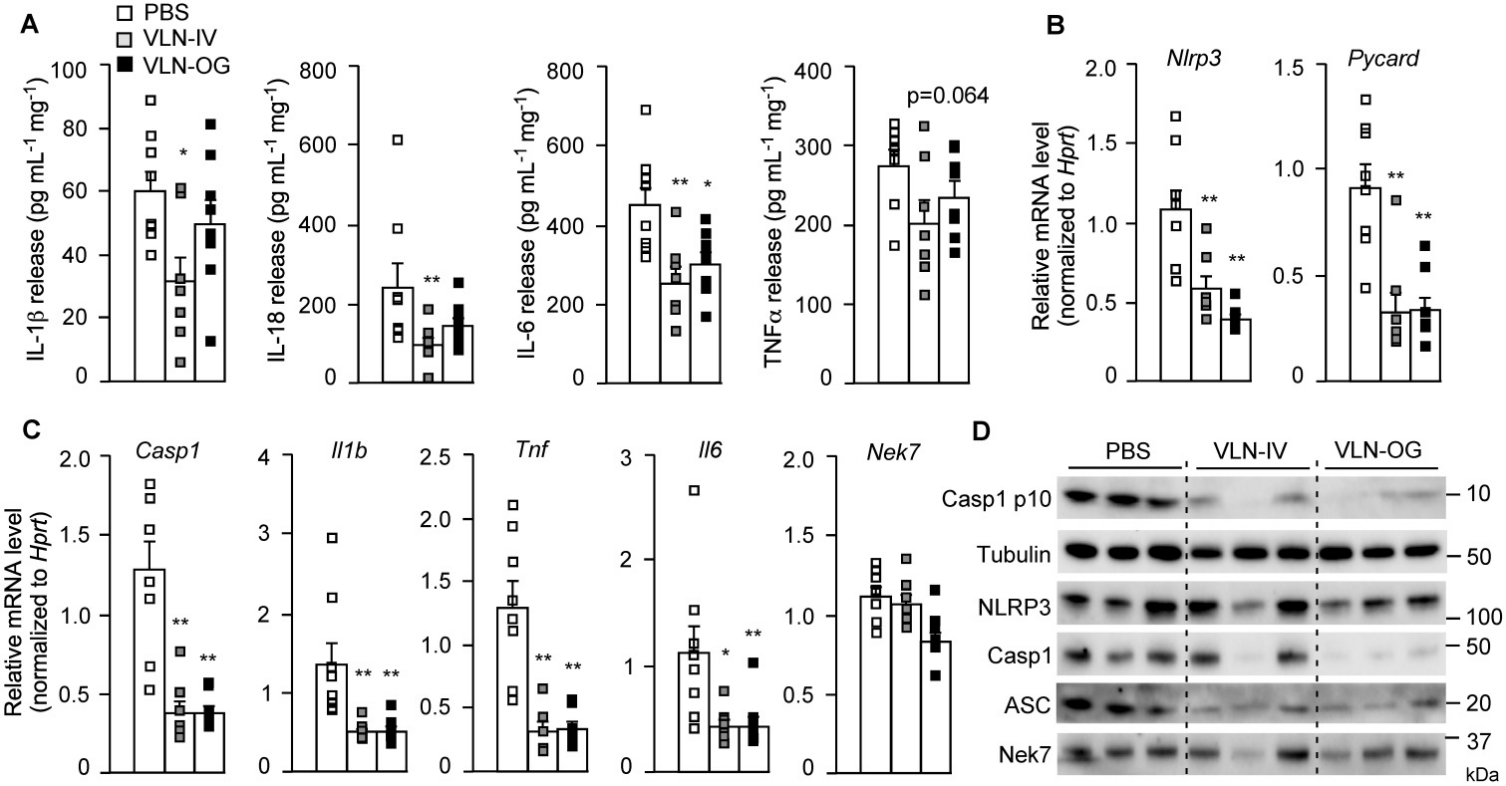
** GC-VLNs reduced inflammation in eWAT of diet-induced obese mice.** The mice described in Figure 5 were sacrificed after 15-week treatment. One piece of eWAT was *ex vivo* cultured, primed with LPS for 3 h, and stimulated with FFA sodium palmitate for 12 h. (**A**) Levels of IL-1β, IL-18, IL-6, and TNFα in the culture media. The cytokine levels were normalized to the protein amount in each cultured eWAT. (**B-C**) Relative mRNA levels of inflammatory genes in eWAT, which were snap frozen during sacrifice. (**D**) Immunoblot analysis of eWAT SVF. Each SVF sample was extracted from eWAT of 2 mice in each group. Data were presented as mean±SEM. * (p < 0.05) and ** (p < 0.01) compared with the control group (bar with white squares).

These eWATs were cultured *ex vivo* and stimulated with LPS and FFA. The release of cytokines, including IL-1β, IL-18, IL-6, and TNFα, from eWAT in VLN-IV mice was decreased, compared to control mice (Figure 7A). The cytokines released from eWAT in VLN-OG mice tended to be lower than in control mice but did not reach statistical significance for most of them (Figure 7A). At the molecular level, both orally and intravenously administered GC-VLNs decreased transcription of the *Nlrp3, Pycard, Casp1, Il1b, Tnf*, and *Il6* genes in eWAT, but had no effects on mRNA level of the *Nek7* gene (Figure 7B-C). At the protein level, the cleaved Casp1 and inflammasome components, including NLRP3, Casp1, and ASC, were much lower in the SVF of GC-VLN treated mice (Figure 7D). Altogether, it seems that both IV- and OG-administered GC-VLNs improved metabolic health and decreased NLRP3 inflammasome activity and inflammation in diet-induced obese mice.

### Lipids in GC-VLNs suppressed activation of the NLRP3 inflammasome

**Figure 8:**
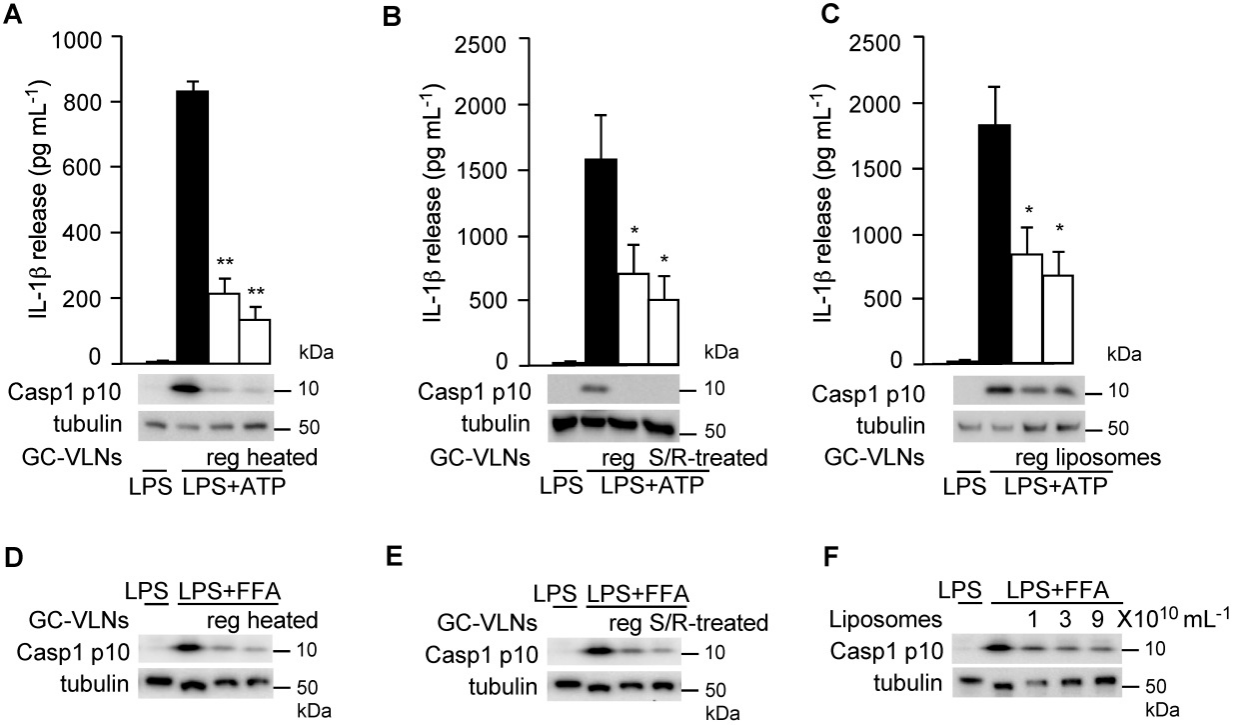
** Lipids from GC-VLNs suppressed NLRP3 inflammasome activation.** (**A** and** D**) GC-VLNs were heated at 95 ºC for 10 min to denature proteins. 9×10^10^ mL^-1^ of regular (reg) or heated GC-VLNs were preincubated with BMDMs for 16 h, followed by NLRP3 inflammasome activation using LPS+ATP (**A**) or LPS+FFA (**D**). (**B** and** E**) GC-VLNs were added with 10 μg mL^-1^ of RNase, subjected to bath sonication at room temperature for 1.5 h, and incubated at 37 ºC for an additional 1 h to degrade RNAs inside the nanoparticles (S/R-treated). 9×10^10^ mL^-1^ of regular (reg) or RNA-depleted (S/R-treated) GC-VLNs were used. (**C** and **F**) Lipids were extracted from GC-VLNs and reassembled into liposomes. 9×10^10^ mL^-1^ of regular (reg) GC-VLNs or liposomes from GC-VLN lipids were preincubated with BMDMs for 16 h, followed by NLRP3 inflammasome activation using LPS+ATP (**C**). In (**F**), different doses of liposomes from GC-VLN lipids were preincubated with cells for 16 h, followed by LPS and FFA treatment to activate the NLRP3 inflammasome. Results were expressed as mean±SEM from three independent experiments. * (p < 0.05) and ** (p < 0.01) compared with LPS+ATP group (black bar).

Next, we tried to determine which category of biomolecules in GC-VLNs was critical in mediating the anti-NLRP3 inflammasome function. GC-VLNs were first heat-treated to denature proteins in the nanoparticles. Such heat-treated GC-VLNs retained the ability to suppress the NLRP3 inflammasome in LPS-primed BMDMs activated by ATP or FFA (Figure 8A and D), indicating that proteins in GC-VLNs were not the key molecules mediating anti-inflammasome function. Second, GC-VLNs were subjected to sonication plus RNase treatment to deplete RNAs. The integrity of these membrane-bound nanoparticles was compromised with the sonication treatment and, as a result, RNase could easily enter to degrade RNAs 19. Approximately 98% of RNAs in GC-VLNs were removed by this treatment (Figure S9). The RNA-depleted GC-VLNs still suppressed the NLRP3 inflammasome (Figure 8B and E), implying that the majority of RNAs in GC-VLNs were not critical in suppressing NLRP3 inflammasome activity. Since neither proteins nor RNAs were critical in inhibiting the NLRP3 inflammasome, it was likely that lipids in GC-VLNs played a role in suppressing activation of the NLRP3 inflammasome. To directly assess the role of lipids in GC-VLNs, total lipids in GC-VLNs were purified and re-assembled into liposomes, which mimic the membrane-enclosed structure of GC-VLNs. Such liposomes potently inhibited the NLRP3 inflammasome activated by ATP or FFA (Figure 8C and F). Therefore, lipids in GC-VLNs are responsible for suppressing activation of the NLRP3 inflammasome.

### Specific phospholipid was identified to inhibit NLRP3 inflammasome activity

During our studies, we noticed that GC-VLNs from the local grocery shop did not consistently suppress NLRP3 inflammasome activation. To ensure consistency and freshness of raw material, garlic chive seeds were purchased and plants were grown in a greenhouse facility; plant leaves were freshly harvested for VLN extraction. However, the anti-inflammatory effects of GC-VLNs still varied - sometimes they exerted strong anti-inflammasome functions (active), but sometimes they lost such effects (inactive) (Figure S10A-B). There was no significant size difference between active and inactive GC-VLNs (Figure S10C). The morphology and vesicular-structure of inactive GC-VLNs (Figure S10D) were similar to those of active GC-VLNs (Figure 1B). Generally, GC-VLNs obtained in the winter-spring season, but not in summer-fall, had potent anti-inflammasome function, although the underlying mechanism was not clear. For all the studies mentioned above, we always used active GC-VLNs. Typically, one big batch of GC-VLNs was extracted and tested for their anti-inflammasome function in BMDMs. If we observed strong anti-inflammasome function, the batch would be aliquoted, stored at -80 ºC, and used for *in vitro* and* in vivo* studies. Anti-inflammasome functions of GC-VLNs were stable after -80 ºC storage but still were subjected to periodical cell culture tests to ensure their bioactivities. The inactive GC-VLNs were usually discarded and used only as negative controls in the GC-VLN comparison analysis.

**Figure 9:**
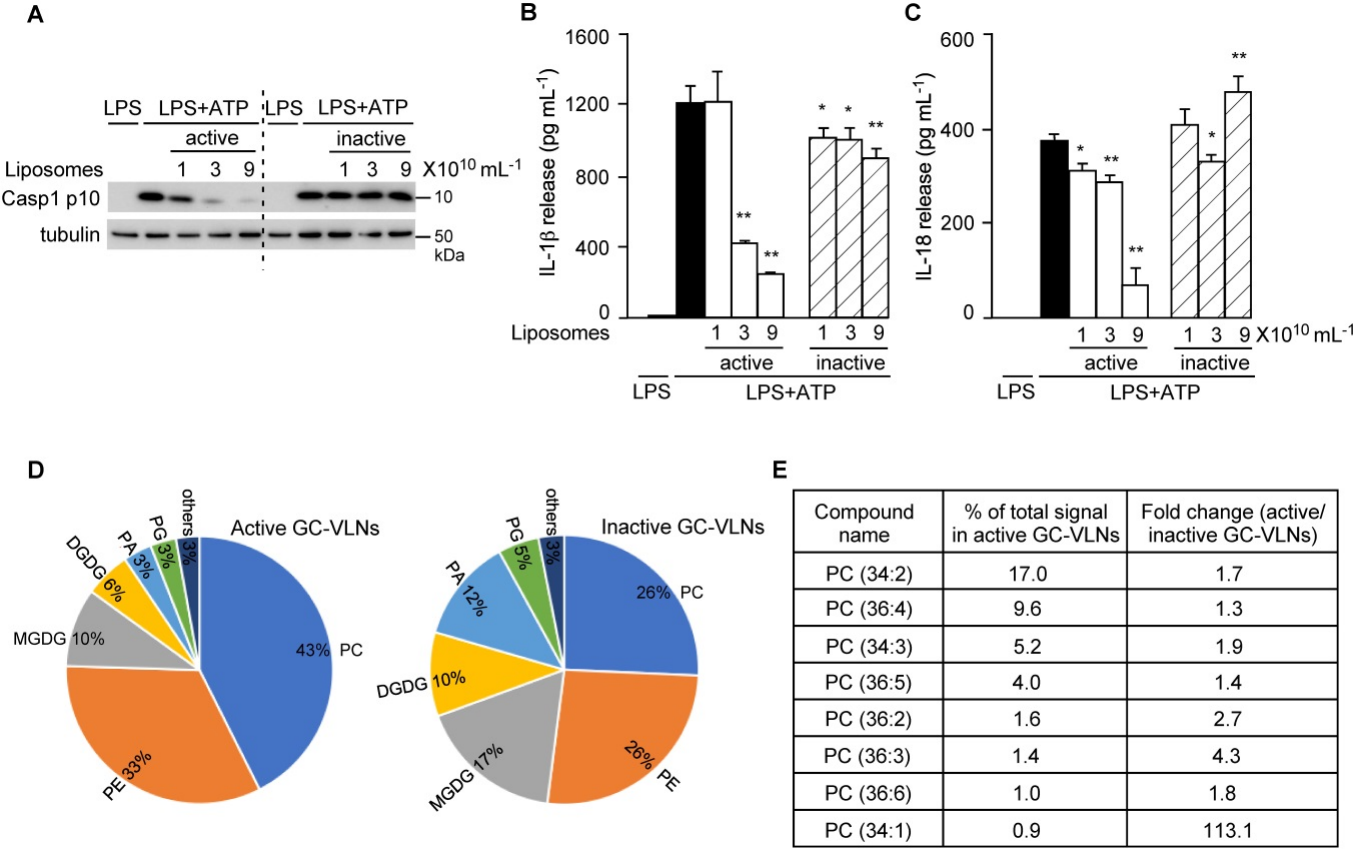
** Lipids from active and inactive GC-VLNs were subjected to lipidomic analysis.** (**A-C**) BMDMs were preincubated with liposomes prepared from lipids of active or inactive GC-VLNs, followed by NLRP3 inflammasome activation. (**A**) Immunoblot analysis of Casp1 p10 in cell lysates. (**B**) Levels of IL-1β in the culture media. (**C**) Levels of IL-18 in the culture media. Results were expressed as mean±SEM from three independent experiments. * (p < 0.05) and ** (p < 0.01) compared with LPS+ATP group (black bar). (**D**) Lipidome comparison of active and inactive GC-VLNs. PC: phosphatidylcholine; PE: phosphatidylethanolamine; PA: phosphatidic acid; PG: phosphatidylglycerol; MGDG: monogalactosyldiacylglycerol; DGDG: digalactosyldiacylglycerol. (**E**) Abundance and fold change of top eight PC species in active GC-VLNs.

After lipids were identified as bioactive molecules in GC-VLNs, total lipids were extracted from active and inactive GC-VLNs and reassembled into liposomes. Only liposomes prepared from active GC-VLNs, but not from inactive GC-VLNs, showed strong inhibitory effects on the downstream events of NLRP3 inflammasome activation, including Casp1 autocleavage (Figure 9A), IL-1β release (Figure 9B), and IL-18 release (Figure 9C). Liposomes from active GC-VLNs also retained inhibitory effects on the secretion of both IL-6 and TNFα, but liposomes from inactive GC-VLNs lost such functions (Figure S11A-B). The distinct anti-inflammatory properties of lipids from active and inactive GC-VLNs provided good sources that allowed us to compare their composition and identify specific active biomolecules.

To compare the composition of lipids from active and inactive GC-VLNs, total lipids from five replicates of these two sources were extracted and subjected to lipidomics analysis. The lipid profiles of active and inactive GC-VLNs were very distinct (Figure 9D and Table S2). Strikingly, the level of phosphatidylcholine (PC) in active GC-VLNs was much higher than the level in inactive GC-VLNs (43% vs 26%). Figure 9E highlights eight PC species that were most abundant in active GC-VLNs and whose levels in active GC-VLNs were meanwhile at least 1.3-fold higher than levels in inactive GC-VLNs. Among these PCs, PC(34:2) and PC(36:4) were most abundant in active GC-VLNs, and the level of PC(34:1) increased most significantly in active GC-VLNs compared to inactive GC-VLNs. Therefore, these three PC lipids were selected for further evaluation. Isoform analysis revealed that in active GC-VLNs, PC(34:2) was 1-palmitoyl-2-linoleoyl-sn-glycero-3-phosphocholine (PC(16:0/18:2)); PC(36:4) was dominantly 1,2-dilinoleoyl-sn-glycero-3-phosphocholine (DLPC, PC(18:2/18:2)), with a tiny amount of 1-oleoyl-2-linolenoyl-sn-glycero-3-phosphocholine; and PC(34:1) was 1-palmitoyl-2-oleoyl-sn-glycero-3-phosphocholine (PC(16:0/18:1)).

**Figure 10:**
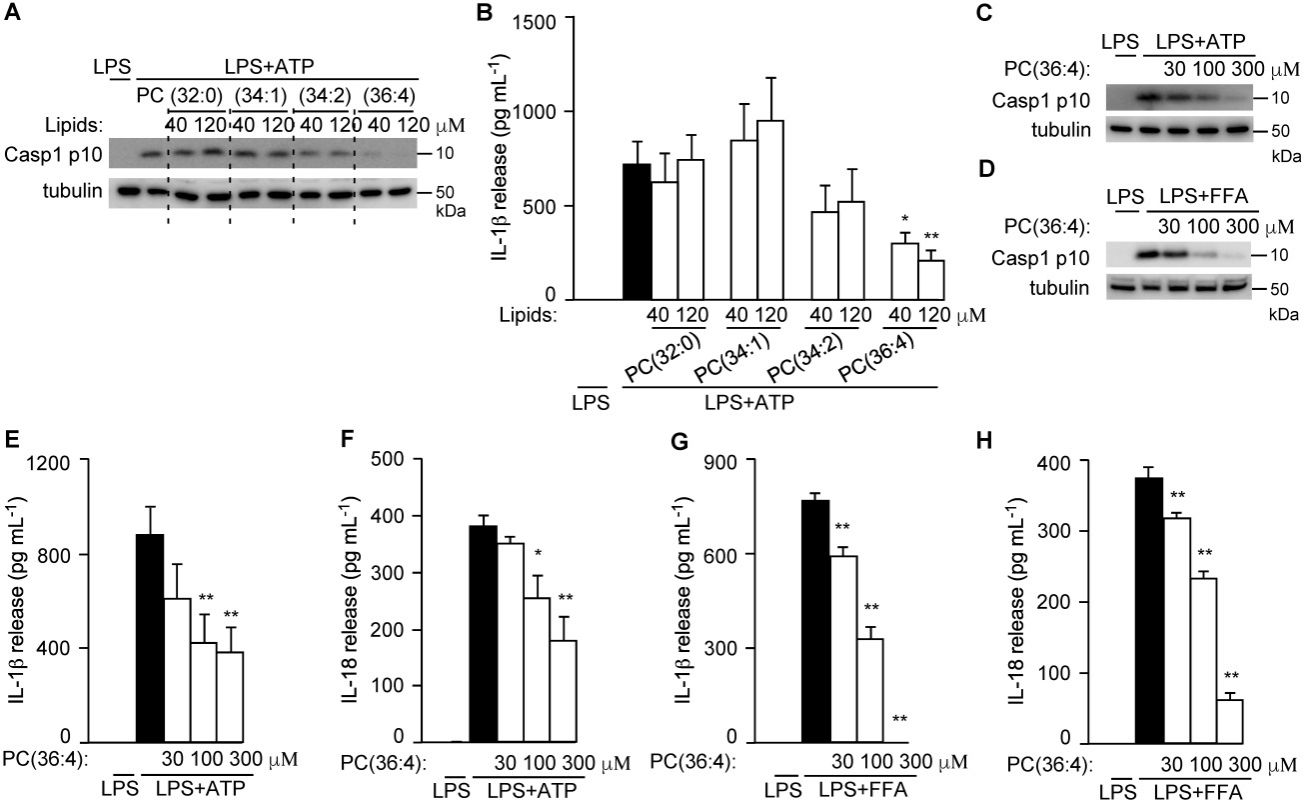
** DLPC was identified to suppress NLRP3 inflammasome activation.** (**A-B**) BMDMs were preincubated with liposomes prepared from PC lipids for 16 h, followed by LPS+ATP treatment to activate the NLRP3 inflammasome. (**A**) Immunoblot analysis of Casp1 p10 in cell lysates. (**B**) Levels of IL-1β in the culture media. (**C-H**) BMDMs were preincubated with DLPC liposomes (PC(36:4)) for 16 h, followed by LPS+ATP or LPS+FFA treatment to activate the NLRP3 inflammasome. Cell lysates were subjected to immunoblot analysis and cell-free media were used for cytokine measurement. Results were expressed as mean±SEM from three independent experiments. * (p < 0.05) and ** (p < 0.01) compared with LPS+ATP group or LPS+FFA group (black bar).

To directly test their roles in NLRP3 inflammasome activation, PC(34:2), PC(34:1), and DLPC (the dominant isoform of PC(36:4) in GC-VLNs) were purchased and assembled into liposomes to test their anti-inflammasome activity. Liposomes prepared from the PC(36:4) isoform DLPC, but not liposomes from control 1,2-dipalmitoyl-sn-glycero-3-phosphocholine (PC (32:0)), PC(34:1), or PC(34:2), potently inhibited Casp1 autocleavage (Figure 10A) and IL-1β release (Figure 10B) upon NLRP3 inflammasome activation in BMDMs. SEM analysis showed that liposomes from PC(36:4) were homogeneous individual nanoparticles (Figure S12A), and NTA showed that these liposomes were approximately 100 nm in diameter (Figure S12B). DLPC-derived liposomes dose-dependently inhibited Casp1 autocleavage (Figure 10C-D), IL-1β release (Figure 10E and G), and IL-18 secretion (Figure 10F and H) when the NLRP3 inflammasome was activated by ATP or FFA in LPS-primed BMDMs. DLPC-derived liposomes also inhibited secretion of both IL-6 and TNFα (Figure S13A-D).

## Discussion

In this study, we found that VLNs were present in commonly consumed *Allium* vegetables. VLNs from garlic chives, but not other *Allium* vegetables we tested, potently suppressed NLRP3 inflammasome activation in primary macrophages. Local injection of GC-VLNs alleviated liver damage and reduced inflammation in the mouse model of acute liver injury. Long-term weekly administration of GC-VLNs through oral gavage or intravenous injection improved metabolic health and reduced chronic inflammation in diet-induced obese mice. The phospholipid DLPC was identified in GC-VLNs to mediate anti-inflammasome functions.

Garlic chive (*A. tuberosum*) is native to Shanxi province located in north-central China and is consumed by people in many Asian countries, including China, Korea, Japan, and India 49. This *Allium* species has been reported to have anti-cancer, anti-oxidant, and anti-inflammatory functions 49, suggesting the medicinal potential of this plant. Over 40 compounds, including organosulfur compounds and flavonoids often found in other *Allium* vegetables, have been identified in garlic chive 50. Among its compounds, thiosulfinates were shown to induce apoptosis of colon cancer cells and tuberoside M suppressed proliferation of leukemia cells 51,52. Studies on the anti-inflammatory function of garlic chives are scarce. Here, we identified a new bioactive agent in garlic chive - GC-VLN, membrane-enclosed vesicle-like nanoparticles containing proteins, RNAs, and lipids (Figure 1). Proteomics analysis of proteins in GC-VLNs (Table S1) demonstrated the presence of a series of plasma membrane proteins and membrane-associated cytosolic proteins, indicating the vesicle-like nature of GC-VLNs, based on the guidelines from Minimal Information for Studies of Extracellular Vesicles 2018 (MISEV2018) 53. These GC-VLNs potently inhibited NLRP3 inflammasome-mediated inflammation in cell culture and animal disease models (Figures 2, 3, 5-7). Therefore, our research revealed that garlic chive's newly identified component GC-VLNs demonstrated a new bioactivity - an anti-NLRP3 inflammasome function, which significantly contributes to the basic understanding of how garlic chive exerts its anti-inflammatory function.

Further investigation revealed that phospholipid DLPC in GC-VLNs mediated the anti-NLRP3 inflammasome function (Figure 8-10). Lipids have been found to either impede or activate the NLRP3 inflammasome. Omega-3 polyunsaturated fatty acids, such as eicosapentaenoic acid (EPA) and docosahexaenoic acid (DHA), inhibit activation of the NLRP3 inflammasome 54, whereas saturated fatty acids, such as palmitic acid, activate the NLRP3 inflammasome 14. Oxidized PCs, including 1-palmitoyl-2-(5-oxovaleroyl)-sn-glycero-3-phosphocholine 55 and 1-O-alkyl-2-acetyl-sn-glycero-3-phosphocholine (also called platelet-activating factor) 56 activate the NLRP3 inflammasome. Our research demonstrated an unprecedented anti-NLRP3 inflammasome function of the phospholipid DLPC in GC-VLNs. Phospholipid PCs have been regarded as membrane bricks since they are the most abundant phospholipids in plasma membranes and subcellular organelles in eukaryotic cells 57. Interestingly, some PCs have been identified to serve directly as signaling molecules. For example, 1-palmitoyl-2-oleoyl-sn-glycerol-3-phosphocholine has been identified as the endogenous ligand for the nuclear receptor peroxisome proliferator-activated receptor α (PPARα) 58. PPARδ in liver regulates the rhythmic level of 1-stearoyl-2-oleoyl-sn-glycerol-3-phosphocholine in serum, which modulates fatty acid use in muscle 59. DLPC has been shown to decrease the release of TNFα in the LPS-treated murine macrophage-like RAW264.7 cell line 60, as well as in LPS-treated Kupffer cells isolated from rats fed an alcohol-containing diet 61. A purified extract of soybean lecithin (94-96% PC), which contains 40-52% DLPC, protects baboons from alcohol-induced fibrosis and cirrhosis 62. Our research revealed another interesting function of DLPC - it inhibits NLRP3 inflammasome activity.

Current NLRP3 inflammasome-related therapies in clinical practice target its cleavage product, IL-1β, using neutralizing IL-1β antibodies (Gevokizumab or Canakinumab), IL-1 receptor antagonist (Anakinra), or the soluble decoy IL-1 receptor (Rilonacept) 63. Anakinra, Rilonacept, and Canakinumab have been approved to treat cryopyrin-associated autoinflammatory syndrome patients 63, and Anakinra has been found to reduce inflammation and plasma glucose levels and enhance insulin secretion in diabetic patients in clinical trials 64. While cytokine-targeting therapies appear to be promising, they target only IL-1β, not IL-18 or other downstream events of the NLRP3 inflammasome. The treatments are costly and sometimes cause adverse effects such as redness, bruising, or pain at the injection site. Some small-molecule chemicals, such as MCC950 65 and Bay 11-7082 66, suppressed assembly and activation of the NLRP3 inflammasome and reduced the severity of NLRP3 inflammasome-driven disorders, such as cryopyrin-associated autoinflammatory syndrome or experimental autoimmune encephalomyelitis in mice. Impeding assembly and activation of the NLRP3 inflammasome blocks all its downstream pathways and theoretically offers greater therapeutic promise to treat NLRP3 inflammasome-driven diseases. However, the bioavailability, potency, and long-term safety of these chemicals in humans have not been well characterized.

GC-VLNs identified in this study potently inhibited all the downstream pathways of the NLRP3 inflammasome through blockage of its assembly and activation (Figure 2, S3, and S4). When administered locally or systemically in mice, GC-VLNs effectively suppressed acute or chronic inflammation in different disease models (Figure 3, 5-7), suggesting the promising efficacy of these dietary nanoparticles *in vivo.* In the obesity study, three months of intravenous or oral administration of GC-VLNs had no detrimental effects on the general health of mice (Figure 5). Orally given GC-VLNs can be absorbed through the digestive tract and accumulated in kidney and eWAT in obese mice. Intravenously administered GC-VLNs also significantly accumulated in kidney and eWAT, implying the possibility of intrinsic tissue selection of GC-VLNs after their absorption (Figure 4). Dietary VLNs have been shown to have distinct biodistribution *in vivo*. For example, grape VLNs accumulated in intestinal stem cells 25, grapefruit VLNs were detected in liver 24, and ginger VLNs were found in the intestinal macrophages 67, liver, and mesenteric lymph nodes 24 after oral gavage in mice. The enrichment of PC in grapefruit VLNs was critical in promoting the accumulation of these nanoparticles in the liver 68. Specific integrin proteins of tumor-derived extracellular vesicles enabled these vesicles to target specific tissues and induced metastasis in these tissues 69. The enriched phosphatidylserine of tumor-derived extracellular vesicles facilitates the recognition and uptake of these vesicles by macrophages 70. Therefore, the unique biodistribution pattern of GC-VLNs reiterates the notion that the lipid composition and/or specific surface proteins of extracellular vesicles and dietary VLNs may mediate their specific uptake by target cells and tissues. Therefore, as a natural nano-carrier, GC-VLN's unique biodistribution, high efficacy, and long-term safety *in vivo* demonstrated in our research suggest their high therapeutic potential. In addition, garlic chives generate abundant GC-VLNs (around 3.4×10^11^ g^-1^, Figure S1), can be easily cultivated at low cost, and have been safely consumed as a daily vegetable in many Asian countries for centuries 26,49. All these properties should facilitate their translational application. Identification of DLPC as an active anti-inflammasome component in GC-VLNs would facilitate quality control of GC-VLNs, as well as guide the grow-condition studies of garlic chive to stabilize the anti-inflammatory function of GC-VLNs in the future. However, as a pure lipid, DLPC alone (nor its derived liposomes) lacks the specific lipid composition and specific surface proteins of GC-VLNs and therefore is not likely to share GC-VLNs' unique biodistribution *in vivo*. Further studies on the bioavailability, efficacy, and safety of DLPC *in vivo* are warranted.

Our previous studies have identified that VLNs from ginger, shiitake mushroom, and honey inhibit the NLRP3 inflammasome 19,20,27. A microRNA miR-4057 was identified in honey VLNs in inhibiting NLRP3 inflammasome activation 20, but the active components in ginger- and shiitake mushroom-derived VLNs have not been elucidated. Our current study identified VLNs from garlic chive - GC-VLNs - with anti-inflammasome function and also revealed that DLPC in GC-VLNs strongly suppresses the NLRP3 inflammasome. Although GC-VLNs shared the same anti-NLRP3 inflammasome as three VLNs in our previous studies, this research has its own importance and novelty as reasoned below:

1) In each of our studies, we screened 5-10 foods from vegetables, fruits, spices, mushrooms, and bee-related products. In our unpublished pilot studies, we have screened more dietary VLNs. In total we have tested VLNs from over 50 dietary sources. So far we have found only four VLNs that inhibit the NLRP3 inflammasome. Therefore, finding of GC-VLNs with anti-inflammasome function, together with our previous studies, establishes an implicit consensus that anti-inflammasome function is unique and possessed only by specific dietary VLNs. 2) This study was designed to thoroughly assess the therapeutic potential of GC-VLNs in combating acute or chronic inflammation using acute liver failure and obesity disease models. Our previous studies mainly used cell culture or acute liver damage models to provide proof-of-concept evidence for the anti-inflammatory functions of dietary VLNs 19,20,27. Other dietary VLN studies have not examined the role of any dietary VLNs in obesity or acute liver failure disease models. Therefore, our current study revealed the possible new therapeutic application of dietary VLNs in the prevention or treatment of chronic inflammation in obesity. 3) This research identified DLPC as an active component of GC-VLNs, compared to microRNAs as the active components of honey-derived VLNs 20, indicating that GC-VLNs have a completely new and different mechanism to inhibit the NLRP3 inflammasome. Our two studies together corroborate that nature develops diverse yet elegant mechanisms to curb dysregulated inflammation in humans. 4) This study highlights the important yet underappreciated functions of lipids in dietary VLNs. Many dietary ELN studies have demonstrated the importance of microRNAs or other components in dietary VLNs. For example, microRNAs in ginger VLNs regulate the composition of the gut microbiota and their functions 68. Depletion of microRNAs in milk VLNs led to altered expression of immune-related genes and exacerbated intestinal inflammation in *Mdr1a*-deficient mice 71. Phytochemical shogaol in ginger VLNs induced transcription of detoxifying/antioxidant genes and protected mice from alcohol-induced liver damage 24. The functions of lipids in dietary VLNs are only beginning to be appreciated. PA(34:2) from ginger-derived VLNs was reported to specifically inhibit the growth of an oral pathogen, *Porphyromonas gingivalis*, through interaction with the outer membrane protein HBP35 of this pathogenic bacterium and suppressing expression of some of its virulence-related genes 72. Our study uncovered another lipid DLPC from GC-VLNs with anti-inflammatory function. In the current study, we examined only the functions of DLPC in inhibiting the NLRP3 inflammasome. It is possible that other lipid species in GC-VLNs could contribute to the anti-inflammasome functions of GC-VLNs, which will be further investigated in the future.

In conclusion, GC-VLNs and their active component DLPC with potent anti-inflammasome activity represent a new type of bioactive agent with potential to treat NLRP3 inflammasome-driven diseases. In the future, it would be also important to elucidate the precise molecular mechanism by which GC-VLNs and DLPC inhibit the NLRP3 inflammasome.

## Materials and Methods

### Macrophage culture

BMDMs from C57BL/6J mice were prepared as described 73. Bone marrow cells from mice were grown in RPMI medium(Corning, Tewksbury, MA, USA) containing 10% fetal bovine serum (FBS; Atlanta Biologicals, Minneapolis, MN, USA, S1150), 20% L929 cell-conditioned medium, 50 μg mL^-1^ PenStrep (Corning, Tewksbury, MA, USA), 2 mM glutamine (Corning), 1 mM sodium pyruvate (Corning), and 10 mM HEPES buffer (Corning). To assess the regulatory role of dietary VLNs in NLRP3 inflammasome activation, BMDMs were preincubated with VLNs for 16 h, then incubated with 10 ng mL^-1^ LPS (InvivoGen, San Diego, CA, USA, tlrl-peklps) for 3 h and 1000 μM sodium palmitate for 12 h (Sigma, St. Louis, MO, USA), or 2.5 mM ATP for 30 min (Sigma), or 5 μM nigericin for 30 min (Enzo Life Sci, Farmingdale, NY, USA), or 0.5% v/v alum for 5 h (ThermoFisher Scientific, Waltham, MA, USA) to activate the NLRP3 inflammasome. To activate the AIM2 inflammasome, BMDMs were incubated with LPS for 3 h, followed by transfection with calf DNA (Sigma, D3664), 2 μg for each well for 2 h. At the end of stimulation, the cells were directly lysed in SDS loading buffer; cell culture media were collected, centrifuged at 300 ×g for 5 min to remove any cell debris, and subjected to cytokine measurement or immunoblot analysis.

### Mice

C57BL/6J and *Nlrp3*^A350VneoR^ mice were purchased from Jackson Laboratory (Bar Harbor, ME, USA) and maintained in a specific pathogen-free facility. Animal experiments were approved by the Institutional Animal Care and Use Committee of University of the Nebraska-Lincoln (Project ID 1421, 1973, and 1936). For the preparation of peritoneal macrophages, 3% (v/v) brewer thioglycolate medium (Sigma) was intraperitoneally injected into C57BL/6J mice; 3 days later, peritoneal macrophages were collected from the peritoneal fluid. To study the biodistribution of GC-VLNs, GC-VLNs covalently labeled with fluorescence dye in near infrared ranges using ExoGlow-Vivo EV labeling kit (System Biosciences, Palo Alto, CA, USA) and free dye control from the same kit were orally or intravenously administered to lean 8-week-old male C57BL/6J mice. 6 h later, the mice were sacrificed to harvest a variety of tissues, which were assessed for fluorescence signals using the Odyssey Clx imaging system (LI-COR Biosciences, Lincoln, NE, USA). The same labeled GC-VLNs were intravenously or orally given to 21-week-old obese male C57BL/6J mice, which were fed with a HFD (Bio-Serv, Flemington, NJ, USA, F3282, 60% of fat calories) for 15 weeks. The mice were sacrificed after 6 h to assess the fluorescence signals in their tissues.

For the acute liver injury model, 8-week-old male C57BL/6J mice were intraperitoneally administered GC-VLNs at a dose of 1×10^10^ g^-1^. After 48 h, mice were intraperitoneally injected with 15 μg kg^-1^ LPS (Sigma, L2630) and 500 mg kg^-1^ GalN (Sigma, 34539) to induce acute liver injury. After 6 h, animals were sacrificed to collect serum and livers for further analysis. The expression of the *Nlrp3* gene is disrupted in *Nlrp3*^A350VneoR^ mice, which therefore serves a *Nlrp3*-deficient mouse strain 7. This mouse strain was crossed with C57BL/6J mice to generate heterozygous mice, which were used to generate wt and *Nlrp3*^-/-^ littermates. 8-week-old wt and *Nlrp3*^-/-^ female littermates were subjected to LPS/GalN injection and sacrificed after 6 h.

For the diet-induced obesity study, 8-week-old male C57BL/6J mice were fed with a HFD (Bio-Serv) and randomly separated into three groups. When the HFD feeding started, the mice in the control group received PBS weekly through both IV and OG, the mice in the VLN-IV group were given 1×10^10^ g^-1^ GC-VLNs through IV and PBS through OG, and the mice in the VLN-OG group were administered 1×10^10^ g^-1^ GC-VLNs through OG and PBS through IV to ensure all the mice were treated with the same administration routes. Body weight of the mice was measured weekly. After the 11-week treatment, the mice were kept individually in a Phenomaster/Labmaster caging system (TSE Systems, Chesterfield, MO, USA) for 48 h. The first 24 h was the adaption period, and the data from the last 24 h were used for calculating food intake, water intake, and movement counts. At the end of the 13-week treatment period, the mice were subjected to an ipGTT. Briefly, the mice were fasted for 6 h, followed by intraperitoneal injection of 1g kg^-1^ glucose (Sigma). The levels of blood glucose were measured every 30 min over a period of 150 min using a Bayer Contour Next blood glucose monitoring system (Parsippany, NJ, USA). After 15 weeks of treatment, the mice were sacrificed to collect serum and eWAT for further analysis.

### Garlic chive

The seeds of garlic chive (*A. tuberosum*) were purchased from Richters Herbs (Goodwood, ON, Canada) and grown in the greenhouse facility at the University of Nebraska-Lincoln (Lincoln, NE, USA). The temperature in the greenhouse facility was controlled between 24 ºC and 27 ºC. Natural sunlight was the main light source, supplemented with three 1000-watt high-pressure sodium lights turned on at 6 AM and off at 8 PM to ensure 14 h of light/day. The plants were grown in a mixture of peat, soil, sand, and vermiculite at the ratio of 5:3:2.5:2.5. The garlic chive leaves were harvested bi-weekly to ensure that maturation levels of leaves were comparable when they were used to extract VLNs.

### Isolation and characterization of VLNs

Bulbs of garlic and purple or white onion and leaves of leek, scallion, and garlic chive were purchased from local grocery shops, washed, and minced. 2-5 g of minced materials were soaked in cold PBS and shredded for 15 s in a kitchen blender. The juice was filtered through a mesh sheet and centrifuged at 500 ×g for 10 min, 2000 ×g for 20 min, and 10,000 ×g for 30 min at 4 ºC. The final supernatant underwent ultracentrifugation at 100,000 ×g for 2 h at 4 ºC using a SW-32TI rotor in an Optima XE-90 ultracentrifuge (Beckman Coulter, Brea, CA, USA). The nanoparticle pellets were washed with cold PBS, resuspended in PBS, and centrifuged at 10,000 ×g for 10 min at 4 ºC to remove any aggregates. VLNs in PBS were passed through a 200 nm Acrodisc filter (Pall Laboratory, Port Washington, NY, USA) to sterilize the solution. For TEM and SEM analysis, VLNs were further purified using a sucrose gradient. Briefly, the nanoparticles in PBS were loaded on sucrose gradients of 8%, 30%, and 60% (w/v) and underwent ultracentrifugation at 150,000 ×g for 16 h at 4 ºC. The fractions enriched with GC-VLNs were collected, diluted in PBS, and subjected to ultracentrifugation at 100,000 ×g for 2 h at 4 ºC. Characterization of VLNs, including size and yield measurement, biomolecule separation, and RNA depletion of GC-VLN, was conducted as described 19,27. For the detergent treatment of VLNs, VLN solutions were added with Triton X-100 (Sigma) at different concentrations, vortexed for 30 s, kept at room temperature for 30 min, and subjected to particle number measurement using a NanoSight NS300 instrument (Malvern, Westborough, MA, USA).

### Labeling and uptake of GC-VLNs

The lipophilic dye DiI (2 μM) was gently mixed with GC-VLNs in PBS for 30 min at 37 ºC to label the membrane lipids. 35-fold more PBS was added to the mixture, and it was subjected to ultracentrifugation at 100,000 ×g for 2 h at 4 ºC to remove free dye. The obtained GC-VLNs were resuspended in PBS and incubated at 37 ºC for 1 h, ready to be incubated with BMDMs. Proteins and RNAs inside GC-VLNs were labeled with ExoGlow protein EV labeling kit and ExoGlow RNA EV labeling kit (System Biosciences), respectively, per manufacturer's protocols. BMDMs were incubated with the fluorescence-labeled GC-VLNs for 16 h or the indicated time in the time-course experiment, washed with PBS 4 times, and fixed with 4% paraformaldehyde (Sigma). Images were taken using an A1R-Ti2 confocal system (Nikon, Melville, NY, USA).

For the distribution studies of GC-VLNs in mice, fluorescence dye from ExoGlow-Vivo EV labeling kit (EXOGV900A-1, System Biosciences) was used to label GC-VLNs at the ratio of 60×10^10^ nanoparticles : 1 μl dye, per manufacturer's protocol. The labeled GC-VLNs were resuspended in 30 mL of PBS and ultra-centrifuged at 100,000 ×g for 2 h at 4 ºC to remove the free dye. The wash step was repeated two times. The obtained GC-VLNs covalently linked to the dye were given to mice through oral gavage or intravenous injection.

### Electron microscopy

SEM analysis of active GC-VLNs and liposomes was carried out for evaluation of particle shape and size range 19,20. GC-VLNs were fixed with 2.5% glutaraldehyde and 1% tannic acid in 0.1 M cacodylate buffer (pH7.2) for 1 h and collected using a MF-Millipore membrane filter (50 nm pore size; Millipore Sigma). The membrane was briefly dried, mounted onto a SEM stub, further dried in the air for 2 h, and sputter-coated with a 2-3 nm thick layer of chromium using a Desk V sputter (Denton Vacuum, Moorestown, NJ, USA). SEM images of GC-VLNs were obtained using a Hitachi S4700 Filed-Emission SEM (Hitachi, Santa Clara, CA, USA).

Ultrastructure TEM was carried out for conformational and vesicular analysis of active and inactive GC-VLNs 20. Pellets of GC-VLNs were incubated with 2.5% glutaraldehyde in 0.1 M cacodylate buffer (pH7.2) at room temperature for 1 h. After two washes with the same buffer, the samples were post-fixed with 1% osmium tetroxide in deionized water at room temperature for 1 h to fix lipids. Fixed VLNs were rinsed in deionized water three times, followed by a dehydration process using a graduated ethanol series, and embedded in Spurr medium mix (Electron Microscopic Sciences, Fort Washington, PA, USA). A Leica UC7 ultramicrotome (Allendale, NJ, USA) was used to cut the samples to obtain ultrathin sections (~90 nm), which were stained with uranyl acetate and lead citrate. Ultrastructural images were collected using a Hitachi H7500 TEM with a bottom-mount AMT digital camera.

### Lipid extraction, lipidomics analysis, and liposome preparation

All lipid extraction reagents were high-performance liquid chromatography (HPLC) grade. Chloroform (Sigma) was supplemented with 0.01% butylated hydroxytoluene (Sigma) to protect polyunsaturated lipids. 0.8 parts GC-VLNs in PBS were added with 1 part chloroform and 2 parts methanol in a glass tube, followed by vigorous shaking. The mixture was added with 1 part chloroform and 1 part water, vigorously shaken, and centrifuged at 1000 ×g for 10 min. The lower layer containing chloroform and lipids was collected in a clean glass tube. The leftover solution was added with 1 part chloroform, mixed well, and centrifuged to collect residue lipids. The chloroform extraction was repeated one more time. All the chloroform layers containing lipids were combined, washed once with 0.25 part of 1 M KCl solution, and once more with 0.25 part of water. The organic phase containing lipids was dried at 60 ºC, with a stream of nitrogen gas blowing over the lipid solution. The dried lipids of five replicates of inactive and active GC-VLNs were sent to the Lipidomics Research Center at the Kansas State University (Manhattan, KS, USA), where analysis was conducted using a direct-infusion electrospray ionization triple quadrupole 4000QTrap mass spectrometer (Applied Biosystems, Foster City, CA, USA) to determine the lipid composition or lipid isoforms 74. Lipid composition was presented as a percentage of the total signal for the molecular species, determined after normalizing the signals to internal standards of the same lipid class.

To assemble lipids from GC-VLNs into liposomes, 500 μl of distilled water were added to the dried lipids, and the mixture was sonicated for 5 min in a bath sonicator (Branson Ultrasonics, Danbury, CT, USA) and further sonicated using a S-450D digital sonifier unit (Branson Ultrasonics) in the mode of 10 s, on and 20 s off for a total of 3 min. The phospholipids PC(32:0), PC(34:1), PC(34:2), and DLPC were purchased from Sigma, dissolved in chloroform, aliquoted, and kept at -20 ºC. To prepare liposomes from these lipids, one aliquot was dried at 60 ºC ,with a stream of nitrogen gas blowing over the lipid solution, and the dried lipids were suspended in 0.9% saline or PBS. The mixture was vigorously vortexed every 15 min for a total of 30 min and extruded through a NanoSizer mini extruder using 100 nm membrane (T&T Scientific, Knoxville, TN, USA) following the manufacturer's instructions.

### Proteomics analysis of GC-VLNs

The pellets of purified GC-VLNs were lysed in NuPAGE™ LDS sample buffer (ThermoFisher Scientific, Waltham, MA, USA) and the lysate was run in a Bolt™ 12% Bis-Tris Plus gel for 10 min, followed by in-gel digestion with trypsin. The obtained peptides were subjected to LC-MS/MS analysis using a RSLCnano system (ThermoFisher Scientific) coupled to a Q-Exactive HF mass spectrometer (ThermoFisher Scientific). The resulting proteomics data were analyzed with Mascot v 2.6.1 (Matrix Science, Boston, MA, USA) using the common contaminants database cRAP (123 entries, www.theGPM.org) and the Uniprot entries for viridiplantae (retrieved on 04/30/2019, 7108828 entries). The identified peptides and proteins were validated using Scaffold v4.8.9 (Proteome Software Inc., Portland, OR). For peptides, if they reached greater than 80.0% probability by the Peptide Prophet algorithm 75 with Scaffold delta-mass correction, their identifications were accepted. For proteins, if they reached 99.0% probability by the ProteinProphet algorithm 76 and contained at least 2 identified peptides, their identifications were established.

### ASC immunofluorescence staining and oligomer analysis

ASC immunofluorescence staining was done as described 19. 1:200 anti-ASC rabbit antibody (Adipogen, San Diego, CA, USA, AG25B0006C100) and 1:200 Alexa Fluor-594-conjugated secondary antibody (Invitrogen, Carlsbad, CA, USA, A-11037) were used for ASC immunofluorescence staining. For the ASC oligomer analysis, BMDMs were lysed 20 min with PBS containing 0.5% Triton X-100, EDTA-free protease inhibitor cocktails (Roche, Indianapolis, IN, USA) and centrifuged at 6,000 ×g at 4 ºC for 15 min. The Triton-soluble supernatant was collected for immunoblot analysis. The Triton-insoluble pellets were rinsed with PBS twice, resuspended in 300 μl PBS containing 2 mM of disuccinimidyl suberate (ThermoFisher Scientific) to crosslink the ASC oligomers at 37 ºC for 30 min, and centrifugated at 6,000 ×g at 4 ºC for 15 min. The pellets containing the crosslinked ASC oligomers were resuspended in SDS loading buffer for immunoblot analysis.

### Tissue H&E staining and liver injury marker measurement

A small piece of liver or eWAT was soaked in 10% formalin solution (VWR, Radnor, PA, USA) during mouse sacrifice. After overnight fixing, the tissues were transferred to cold PBS and submitted to the Veterinary Diagnostic Laboratory at the University of Nebraska-Lincoln for further processing. Briefly, the tissues were embedded in paraffin, cut into 8 μm thick sections, and subjected to routine H&E staining. The levels of liver injury markers in serum, including AST and ALT, were analyzed using a Vitros-250 Chemistry Analyzer (Johnson & Johnson, New Brunswick, NJ, USA).

### Tissue lysate preparation and immunoblot analysis

Tissues were minced in lysis buffer (150 mM NaCl, 50 mM Tris-HCl, pH7.5, 0.5% NP-40) supplemented with protease inhibitor, homogenized using a mini-homogenizer, and gently rotated for 30 min at 4 ºC. The obtained tissue lysates were centrifuged at 18,000 ×g at 4 ºC for 20 min, and the supernatant was collected and measured for protein concentration. Tissue lysates that contained equal amounts of proteins were loaded on the NuPAGE Bis-Tris protein gel (Invitrogen) for immunoblot analysis 19. Primary antibodies used were anti-NLRP3 mouse antibody (Adipogen, AG20B0014C100, 1:1000); anti-ASC rabbit antibody (Adipogen, AG25B0006C100, 1:1000); anti-Casp1 (p10) mouse antibody (Adipogen, AG20B0044C100, 1:1000); anti-tubulin rabbit polyantibody (Santa Cruz, Dallas, TX, USA, SC-5286, 1:200); anti-Nek7 rabbit antibody (Abcam, Cambridge, MA, USA, ab133514, 1:10000); and anti-IL-1β goat antibody (R&D systems, Minneapolis, MN, USA, AF401NA, 1:2000).

### Quantitative PCR (qPCR)

mRNAs were extracted using RNA-bee (Tel-Test, Friendswood, TX, USA) per the manufacturer's instructions. 1 μg of total RNA was used for reverse transcription using a high-capacity cDNA Reverse Transcription Kit (Applied Biosystems, Foster City, CA, USA), followed by qPCR analysis using a CFX Connect Real-time system (Bio-Rad, Hercules, CA, USA). The genes of interest were normalized to the *Hprt* gene.

### Cytokine measurement and LDH release

Cytokine analysis and LDH release were performed as described 19. The release of LDH in medium was assessed using a CytoTox 96 Nonradioactive Cytotoxicity Assay kit (Promega, Madison, WI, USA). Cytokine level was measured using ELISA kits, including IL-1β (eBioscience, San Diego, CA, USA, 88701388), IL-18 (MBL, Woburn, MA, USA, D042-3), TNFα (BioLegend, San Diego, CA, USA, 430901), and IL-6 (BioLegend, 431301).

### SVF preparation

Upon sacrifice, eWAT was soaked in Hank's balanced salt solution (HBSS, Sigma) on ice. After being washed in cold HBSS two times, eWAT from 2 mice in the same group was combined and minced in HBSS containing 1 mg mL^-1^ type I collagenase (Sigma), followed by gently shaking at 37 ºC for 30 min. Every 15 min, the tissue samples were further mixed using a plastic pipette. After enzymatic digestion, the samples went through a cell strainer to remove any residue tissues. Cells were centrifuged at 300 ×g for 10 min to separate SVF pellets from floating adipocytes.

### Flow cytometry analysis

SVF cells or BMDMs were blocked with TrueStain fcX (BioLegend) for 15 min on ice in PBS containing 0.5% FBS, 2 mM EDTA. SVF cells were incubated with F4/80-APC antibody (BioLegend) or rat isotype-APC control antibody (BioLegend) for 30 min on ice. BMDMs were incubated with both F4/80-APC antibody (BioLegend) and CD11-b-PE antibody (BioLegend) for 30 min on ice. Both rat isotype-APC and rat isotype-PE control antibodies (BioLegend) were used in BMDMs as controls. The cells were washed and subjected to flow cytometry analysis using a DxP10 FACSort flow cytometer (BD Biosciences, San Jose, CA, USA). The data were analyzed with FlowJo (BD Biosciences).

### Statistics

The results of cell culture experiments were analyzed using Excel software. A two-tailed t test was employed to compare differences between two conditions. Each cell culture experiment was repeated 3 or 4 times. R version 3.6.0 (R Core Team, The R Foundation for Statistical Computing, Vienna, Austria) 77 was used to analyze the results of animal experiments. The normality of the data was evaluated using a Shapiro-Wilks test. Because a low p-value from the Shapiro-Wilks test suggested that the data were not normally distributed, they were evaluated using a nonparametric Mann-Whitney test. Otherwise, the data were assumed to be normally distributed and analyzed using a two-tailed t test. For all statistical analyses, p < 0.05 was indicated by * and considered significant. p < 0.01 was indicated by **.

## Supplementary Material

Supplementary figures and tables.

## Abbreviations

AIM2: Absent in Melanoma 2
ALT: alanine aminotransferase
ASC: apoptotic speck protein containing a caspase recruitment domain
AST: aspartate aminotransferase
ATMs: adipose tissue macrophages
ATP: adenosine triphosphate
AUC: area under the curve
BAT: brown adipose tissue
BMDMs: bone marrow-derived macrophages
Casp1: caspase-1
CLSs: crown-like structures
DAPI: 4′,6-diamidino-2-phenylindole
DGDG: digalactosyldiacylglycerol
DHA: docosahexaenoic acid
DiI: 1,1'-dioctadecyl-3,3,3',3'-tetramethylindocarbocyanine perchlorate
DLPC: 1,2-dilinoleoyl-sn-glycero-3-phosphocholine
ELISA: enzyme-linked immunosorbent assay
EPA: eicosapentaenoic acid
eWAT: epididymal white adipose tissue
FBS: fetal bovine serum
FFAs: free fatty acids
FI: fluorescence intensity
GalN: D-galactosamine
GC-VLNs: garlic chive-derived VLNs
GI: gastrointestinal
HBSS: Hank's balanced salt solution
H&E: hematoxylin and eosin
HFD: high-fat diet
HPLC: high-performance liquid chromatography
Hprt: hypoxanthine guanine phosphoribosyl transferase
IL: interleukin
ipGTT: intraperitoneal glucose tolerance test
IV: intravenous injection
LDH: lactate dehydrogenase
LPS: lipopolysaccharide
MGDG: monogalactosyldiacylglycerol
Nek7: NIMA-related kinase 7
NF-κB: nuclear factor-κB
NIMA: never in mitosis gene a
NLR: nucleotide-binding domain and leucine-rich repeat related
NLRP3: NLR family, pyrin domain containing 3
nt: nucleotides
NTA: Nanoparticle tracking analysis
OG: oral gavage
PA: phosphatidic acid
PBS: phosphate-buffered saline
PC: phosphatidylcholine
PE: phosphatidylethanolamine
PG: phosphatidylglycerol
PPARα: peroxisome proliferator-activated receptor α
qPCR: quantitative PCR
SEM: scanning electron microscopy
SVF: stromal vascular fraction
TEM: transmission electron microscopy
TLC: thin layer chromatography
TNFα: tumor necrosis factor alpha
VLNs: vesicle-like nanoparticles
wt: wildtype
