# Supplementary material for: Therapeutic potential of garlic chive-derived vesicle-like nanoparticles in NLRP3 inflammasome-mediated inflammatory diseases
Source: Theranostics. 2021 Sep 7;11(19):9311–30. doi: 10.7150/thno.60265 (PMC8490522; doi:10.7150/thno.60265)
Supplement: Supplementary file 1 — Supplementary figures and tables. [file thnov11p9311s1.pdf]

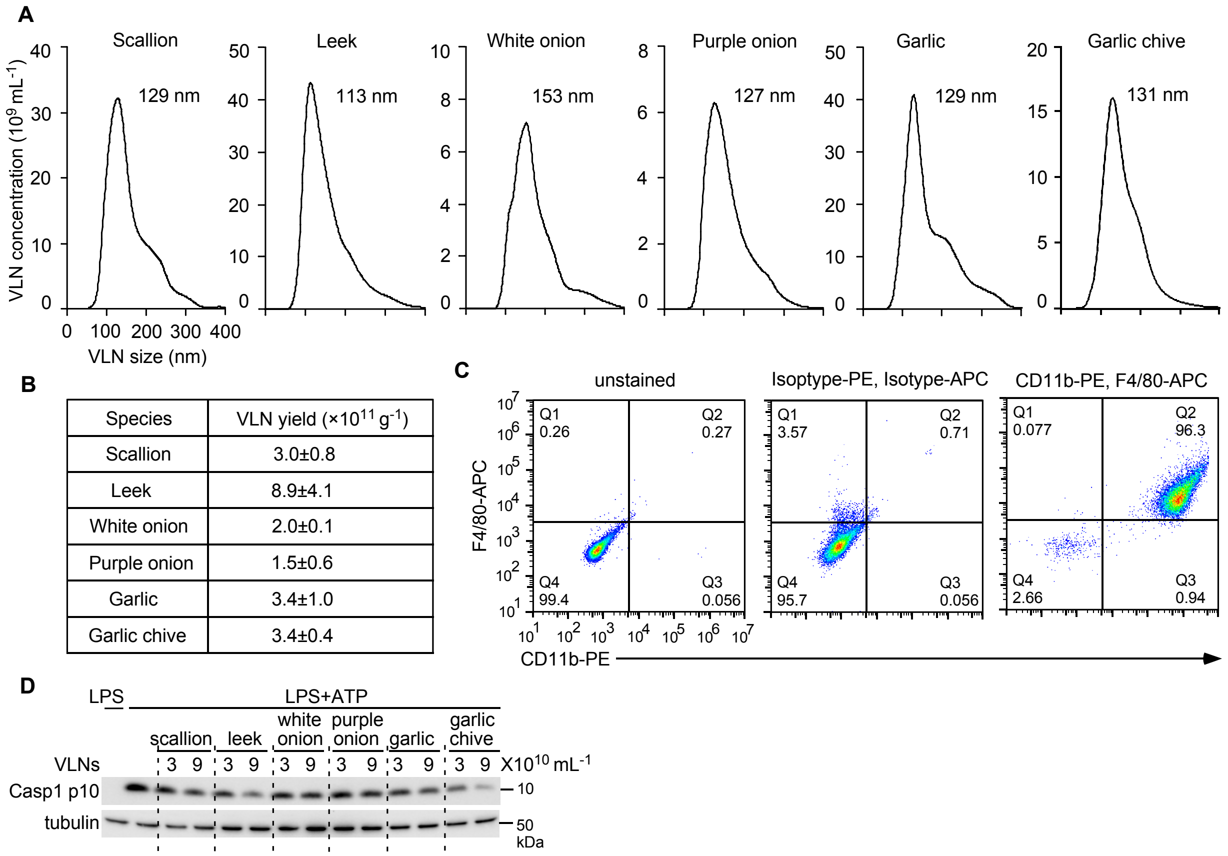

Figure S1. VLNs were present in *Allium* vegetables. (**A**) NTA analysis showed the sizes of VLNs from *Allium* vegetables using a NanoSight NS300. (**B**) Yields of VLNs from six *Allium* vegetables. (**C**) Flow cytometry analysis of BMDMs using F4/80-APC and CD11b-PE antibodies. Isotype-APC and isotype-PE antibodies were used as antibody controls. (**D**) Immunoblot analysis of Casp1 p10 in cell lysates of BMDMs preincubated with *Allium*-derived VLNs for 16 h, followed by LPS priming and ATP treatment to activate the NLRP3 inflammasome. Tubulin was included to show equivalent loading in immunoblot analysis.

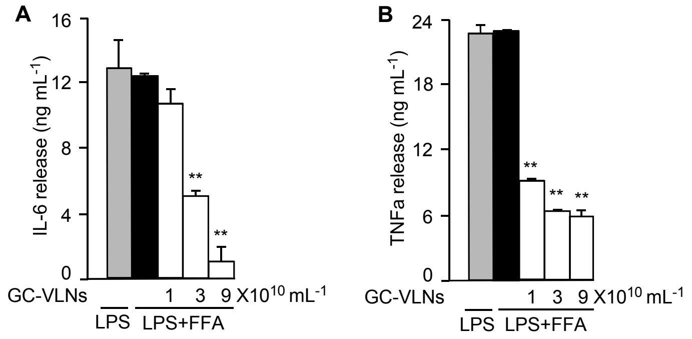

Figure S2. GC-VLNs inhibited release of IL-6 and TNFα. The cell-free culture media from Figure 2 were used to measure the levels of IL-6 (**A**) and TNFα (**B**) using ELISA kits. Results were expressed as mean±SEM from three independent experiments. * ($p<0.05$) and ** ($p<0.01$) compared with LPS+FFA group (black bar).

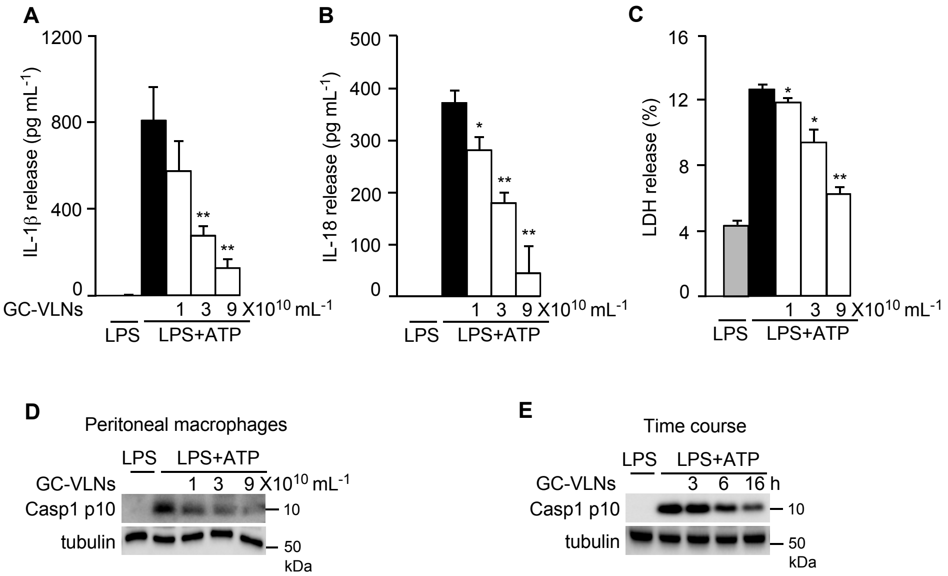

Figure S3. GC-VLNs inhibited NLRP3 inflammasome activated by ATP. (**A-C**) BMDMs were preincubated with GC-VLNs for 16 h, primed with LPS for 3 h, and stimulated with ATP for 30 min to activate the NLRP3 inflammasome. The levels of IL-1β (**A**) and IL-18 (**B**) in the media were measured. (**C**) Pyroptotic cell death. (**D**) Immunoblot analysis of Casp1 p10 in lysates of peritoneal macrophages preincubated with GC-VLNs, followed by NLRP3 inflammasome activation. (**E**) Immunoblot analysis of Casp1 p10 in lysates of BMDMs preincubated with GC-VLNs for 3, 6, or 16 h, followed by NLRP3 inflammasome activation. Results were expressed as mean±SEM from three independent experiments. * ($p<0.05$) and ** ($p<0.01$) compared with LPS+ATP group (black bar).

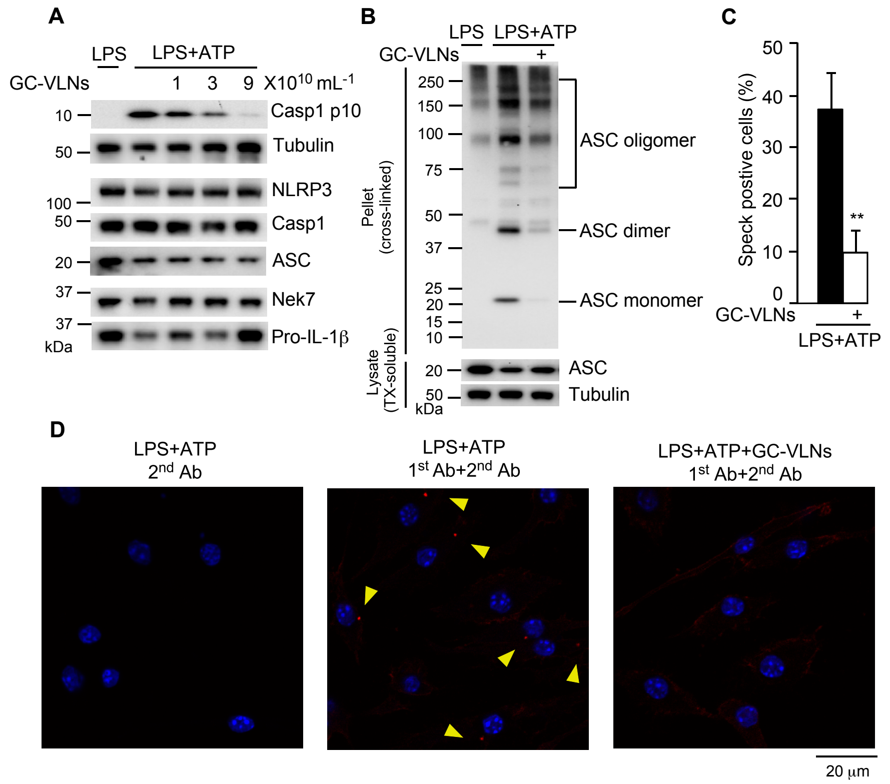

Figure S4. GC-VLNs impeded formation of NLRP3 inflammasome complex. (**A**) Immunoblot analysis of cell lysates of BMDMs preincubated with GC-VLNs, followed by LPS+ATP treatment to activate the NLRP3 inflammasome. (**B**) Immunoblot analysis of Triton X-100 (TX)-soluble fraction and Triton X-100 insoluble fraction (pellet) in the ASC oligomerization assay. The pellet was cross-linked using disuccinimidyl suberate to retain ASC dimers and oligomers. 3×10^{10} mL^{-1} of GC-VLNs were used. (**C**) Quantification and (**D**) Representative images of the speck positive BMDMs in ASC immunofluorescence staining. Casp1 inhibitor VX765 (10 μM) was added to cells 30 min before ATP treatment to stabilize the inflammasome complex, which was then stained as a single speck (yellow arrows) with an anti-ASC antibody. 1×10^{10} mL^{-1} of GC-VLNs were used. 1st Ab: anti-ASC primary antibody; 2nd Ab: Alexa Fluor-594 conjugated secondary antibody. The cell nuclei were stained with

4′,6-diamidino-2-phenylindole (DAPI). Results were expressed as mean±SEM from three independent experiments. ** ($p<0.01$) compared with LPS+ATP group (black bar).

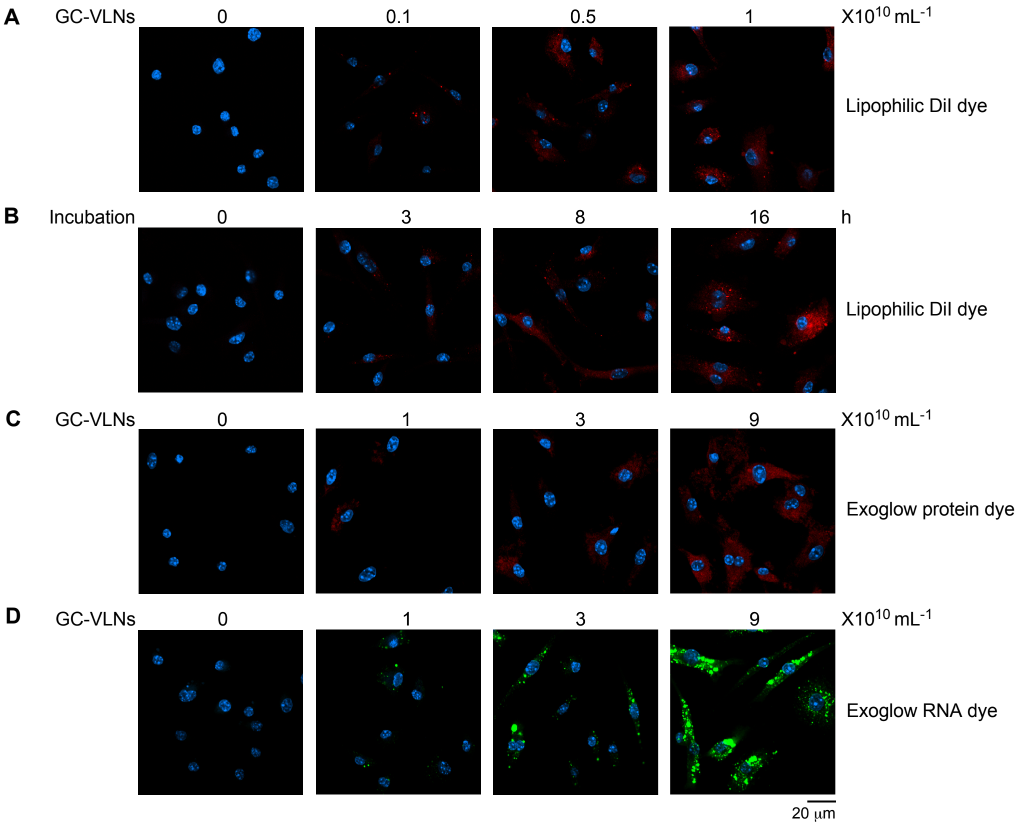

Figure S5. GC-VLNs were taken up by BMDMs. (**A**) Lipophilic dye DiI-labeled GC-VLNs were incubated with BMDMs for 16 h. (**B**) 1×10^{10} mL^{-1} of DiI-labeled GC-VLNs were incubated with BMDMs for different time. (**C**) Exoglow protein dye-labeled GC-VLNs were incubated with BMDMs for 16 h. (**D**) Exoglow RNA dye-labeled GC-VLNs were incubated with BMDMs for 16 h. After nanoparticle incubation, cells were washed with PBS extensively and fixed with formaldehyde. Images were acquired with an A1R-Ti2 confocal system (Nikon). DAPI was included to stain nuclei.

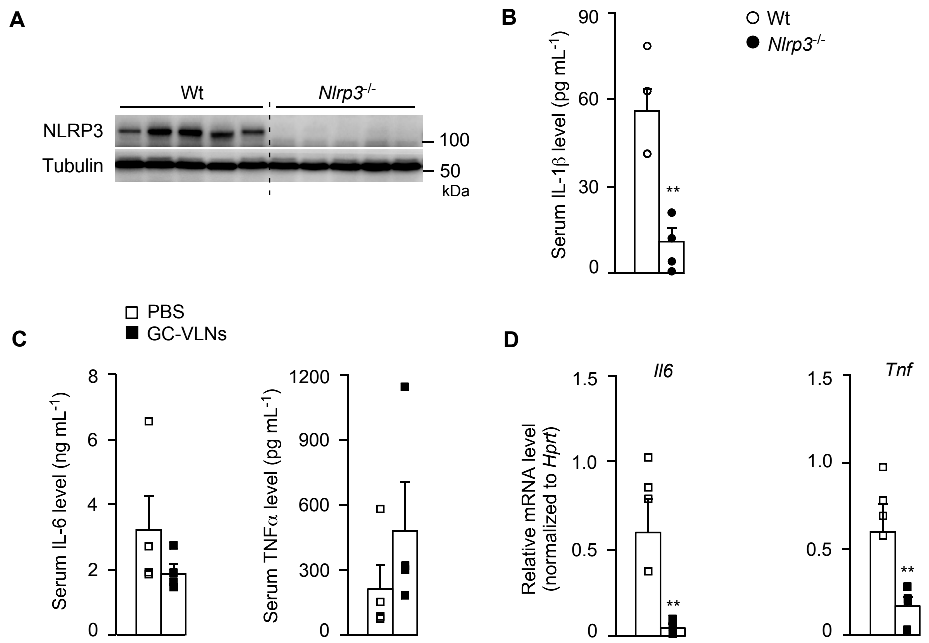

Figure S6. Chemical-induced acute liver injury in wt and *Nlrp3*-deficient mice and effects of GC-VLNs on IL-6 and TNFα in this disease model. 8-week-old female wt and *Nlrp3*-deficient littermates were intraperitoneally injected with a mixture of LPS and GalN and sacrificed after 6 h. N=5/group. (**A**) Immunoblot analysis of liver lysates of wt and *Nlrp3* deficient littermates. (**B**) Levels of IL-1β in serum of wt and *Nlrp3*-deficient littermates. (**C**-**D**) The serum and liver samples from Figure 3 were subjected to cytokine measurement and gene expression analysis, respectively. (**C**) Levels of IL-6 and TNFα in serum. (**D**) Relative mRNA levels of *Il6* and *Tnf* genes in the livers. The housekeeping gene *Hprt* was used to normalize mRNA levels. Data were presented as mean±SEM. * ($p<0.05$) and ** ($p<0.01$) compared with the control group (bar with white squares or circles).

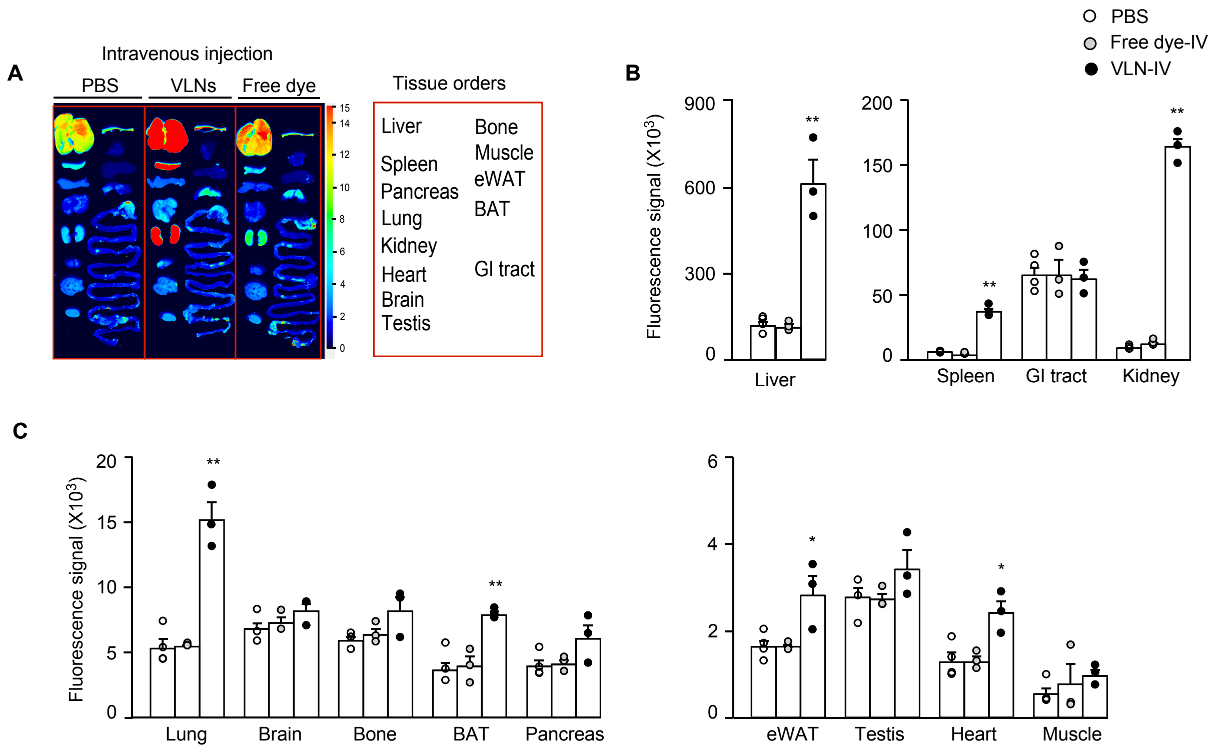

Figure S7. Distribution of intravenously injected GC-VLNs in lean mice. GC-VLNs were covalently labeled with a fluorescence dye in near infrared ranges. The labeled GC-VLNs or free dye were intravenously administered at 3,500 FI g^{-1} to 8-week-old male C57BL/6J mice. The solvent PBS was intravenously administered to the control group. 6 h later, the mice were sacrificed to collect tissues to measure the fluorescence signals in each type of tissue. N=3-4/group. (**A**) Representative images of mouse tissues under Licor Odyssey Clx image system. (**B-C**) Fluorescence signal intensity of mouse tissues collected from mice. VLN-IV: mice intravenously injected with GC-VLNs. Data were presented as mean±SEM. * ($p<0.05$) and ** ($p<0.01$) compared with the control PBS group (bar with white circles).

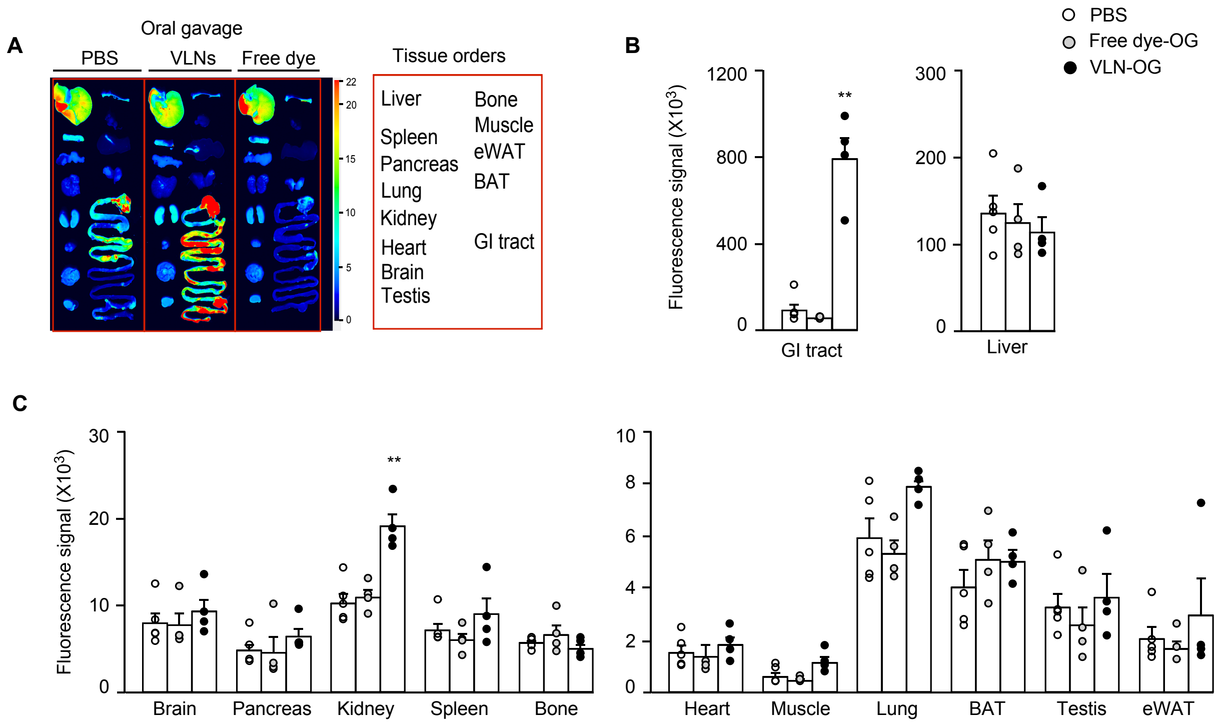

Figure S8. Distribution of orally administered GC-VLNs in lean mice. GC-VLNs were covalently labeled with a fluorescence dye in near infrared ranges. The labeled GC-VLNs were orally administered at 60,000 FI g^{-1} to 8-week-old male C57BL/6J mice. The free dye at 7,000 FI g^{-1} was orally given to the free dye group, and the solvent PBS was orally given to the control group. 6 h later, the mice were sacrificed to collect tissues to measure the fluorescence signals in each type of tissue. N=4-5/group. (**A**) Representative images of mouse tissues under Licor Odyssey Clx image system. (**B-C**) Fluorescence signal intensity of mouse tissues collected from mice. VLN-OG: mice orally gavaged with GC-VLNs. Data were presented as mean±SEM. * ($p<0.05$) and ** ($p<0.01$) compared with the control PBS group (bar with white circles).

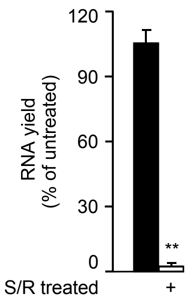

Figure S9. Majority of RNAs in GC-VLNs were depleted after sonication plus RNase treatment. GC-VLNs were added with 10 μg mL^{-1} of RNase, subjected to bath sonication at room temperature for 1.5 h, and incubated at 37 °C for an additional 1 h to degrade RNAs inside the nanoparticles (S/R-treated). The resulting GC-VLNs were subjected to RNA extraction, followed by RNA yield measurement. The untreated same amount of GC-VLNs was subjected to RNA extraction and served as controls. Data were presented as mean±SEM from three independent experiments. ** ($p<0.01$) compared with the control samples (black bar).

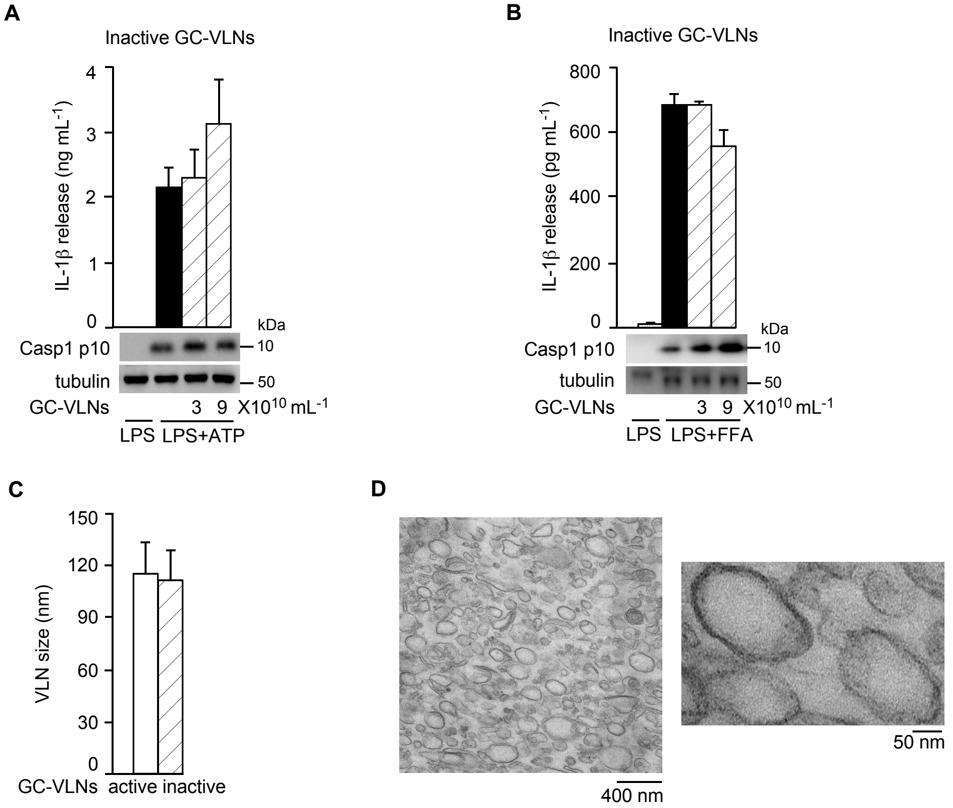

Figure S10. Identification of inactive GC-VLNs. (**A-B**) Inactive GC-VLNs had no inhibitory effects on IL-1β release and Casp1 autocleavage when the NLRP3 inflammasome was activated by LPS+ATP (**A**) or LPS+FFA (**B**). (**C**) NTA analysis showed comparable sizes of active and inactive GC-VLNs. N=11-13/group. (**D**) Ultrastructure TEM images of inactive GC-VLNs. Data were presented as mean±SEM.

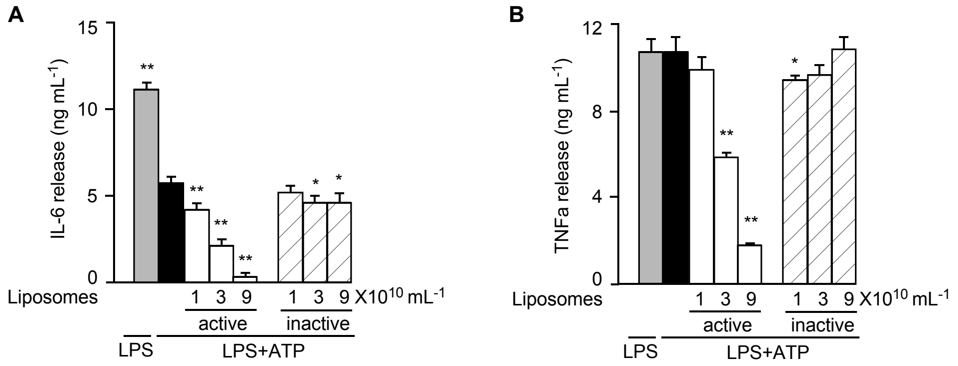

Figure S11. Effects of lipids from active and inactive GC-VLNs on release of IL-6 and TNFα. Liposomes prepared from lipids of active GC-VLNs, but not liposomes from inactive GC-VLN lipids, inhibited release of IL-6 (**A**) and TNFα (**B**). Results were expressed as mean±SEM from three or four independent experiments. * ($p<0.05$) and ** ($p<0.01$) compared with LPS+ATP group (black bar).

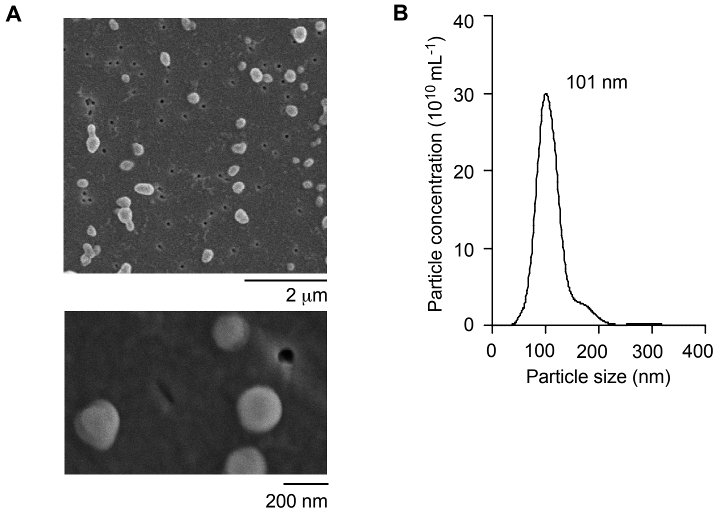

Figure S12. DLPC were verified to form nanoparticles. (**A**) Representative SEM images of liposomes prepared from DLPC. (**B**) NTA analysis showed the size of DLPC-derived liposomes using a NanoSight NS300.

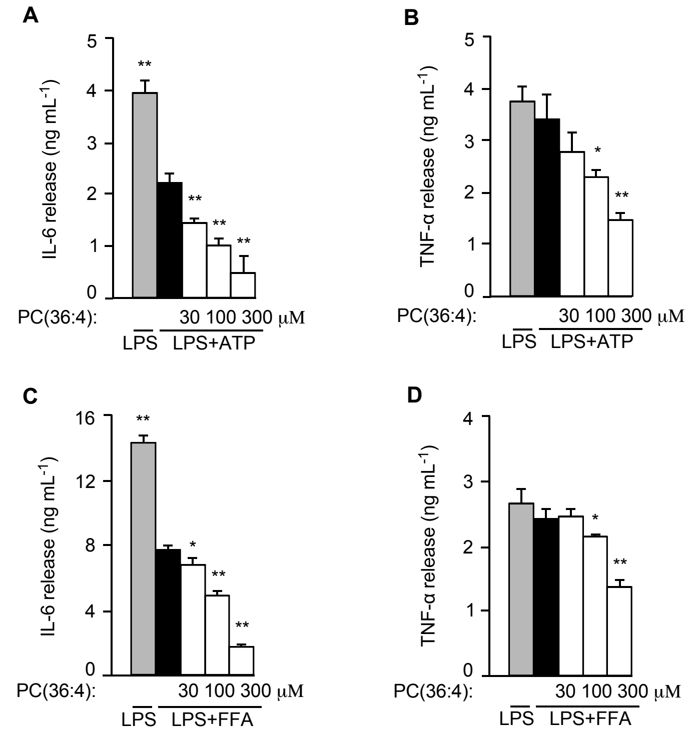

Figure S13. DLPC liposomes suppressed release of IL-6 and TNFα in BMDMs. BMDMs were preincubated with DLPC liposomes (PC(36:4)) for 16 h, followed by treatment of LPS+ATP (**A-B**) or LPS+FFA (**C-D**) to activate the NLRP3 inflammasome. Cell-free media were used to measure the levels of IL-6 and TNFα. Results were expressed as mean±SEM from three independent experiments. * ($p<0.05$) and ** ($p<0.01$) compared with LPS+ATP or LPS+FFA group (black bar).

Table S1 Protein profiles of GC-VLNs

List of Proteins identified using Mascot v2.6.1 and summarized in Scaffold v4.8.9. The databases searched were the cRAP_20150130.fasta and uniprot-viri
Proteins with shared peptides are grouped together into clusters and indicated with the same #

			Total Unique Peptide Count		
#	Identified Proteins (922)	Accession Numbe	replicate1	replicate2	replicate3
1.1	Ribulose bisphosphate carboxylase large chain (Fragment) OS=Tabebuia heterophylla OX=39	RBL_TABHE	23	24	25
1.2	Ribulose bisphosphate carboxylase large chain (Fragment) OS=Lobelia sp. OX=4383 GN=rbcL	RBL_LOBSP	14	13	16
1.3	Ribulose bisphosphate carboxylase large chain (Fragment) OS=Ephedra sinica OX=33152 GN	RBL_EPHSI	18	18	20
1.4	Ribulose bisphosphate carboxylase large chain (Fragment) OS=Fleroya rubrostipulata OX=43	RBL_FLERU	19	17	17
1.5	Ribulose bisphosphate carboxylase large chain (Fragment) OS=Kigelia africana OX=70070 GN	RBL_KIGAF	20	21	22
1.6	Ribulose bisphosphate carboxylase large chain (Fragment) OS=Hedera helix OX=4052 GN=rb	RBL_HEDHE	18	17	19
1.7	Ribulose bisphosphate carboxylase large chain (Fragment) OS=Callicarpa dichotoma OX=28!	RBL_CALDI	13	13	14
1.8	Ribulose bisphosphate carboxylase large chain OS=Acorus americanus OX=263995 GN=rbcL	RBL_ACOAM (+1)	25	24	26
1.9	Ribulose bisphosphate carboxylase large chain (Fragment) OS=Bauera rubioides OX=23051 (	RBL_BAURU	17	17	18
1.10	Ribulose bisphosphate carboxylase large chain (Fragment) OS=Serenoa repens OX=4722 GN=	RBL_SERRE	25	27	27
1.11	Ribulose bisphosphate carboxylase large chain OS=Physcomitrella patens subsp. patens OX=	RBL_PHYPA	21	21	22
1.12	Ribulose bisphosphate carboxylase large chain OS=Jasminum nudiflorum OX=126431 GN=rl	RBL_JASNU			17
1.13	Ribulose bisphosphate carboxylase large chain OS=Lolium perenne OX=4522 GN=rbcL PE=3	RBL_LOLPR	20	22	22
1.14	Ribulose bisphosphate carboxylase large chain (Fragment) OS=Convolvulus tricolor OX=412	RBL_CONTR	16	17	18
1.15	Ribulose bisphosphate carboxylase large chain OS=Phytolacca americana OX=3527 GN=rbcL	RBL_PHYAM		13	
1.16	Ribulose bisphosphate carboxylase large chain (Fragment) OS=Cornus kousa OX=28501 GN=	RBL_CORKO		11	11
1.17	Ribulose bisphosphate carboxylase large chain (Fragment) OS=Symphoricarpos albus OX=13	RBL_SYMAL	16	16	
1.18	Ribulose bisphosphate carboxylase large chain (Fragment) OS=Eriodictyon californicum OX=	RBL_ERICA		14	
1.19	Ribulose bisphosphate carboxylase large chain (Fragment) OS=Chiococca alba OX=28527 GN	RBL_CHIAL	16	17	18
1.20	Ribulose bisphosphate carboxylase large chain OS=Cuscuta sandwichiana OX=197374 GN=rl	RBL_CUSSA	10	10	
1.21	Ribulose bisphosphate carboxylase large chain OS=Stigeoclonium helveticum OX=55999 GN	RBL_STIHE		10	
1.22	Ribulose bisphosphate carboxylase large chain (Fragment) OS=Pelargonium grandiflorum O)	RBL_PELGR	16	16	18
1.23	Ribulose bisphosphate carboxylase large chain OS=Bryopsis maxima OX=3129 GN=rbcL PE=3	RBL_BRYMA	14	13	15
1.24	Ribulose bisphosphate carboxylase large chain OS=Viburnum acerifolium OX=4205 GN=rbcl	RBL_VIBAC		19	
1.25	Ribulose bisphosphate carboxylase large chain OS=Barbarea verna OX=50458 GN=rbcL PE=3	RBL_BARVE		15	
1.26	Ribulose bisphosphate carboxylase large chain (Fragment) OS=Nicandra physalodes OX=331	RBL_NICPH	13		
1.27	Ribulose bisphosphate carboxylase large chain OS=Cuscuta exaltata OX=476139 GN=rbcL PE	RBL_CUSEX			14
2.1	ATP synthase subunit beta, mitochondrial OS=Nicotiana plumbaginifolia OX=4092 GN=ATPI	ATPBM_NICPL	25	23	24

2.2	ATP synthase subunit beta, mitochondrial OS=Zea mays OX=4577 GN=ATPB PE=2 SV=1	ATPBM_MAIZE	24	24	25
2.3	ATP synthase subunit beta, mitochondrial OS=Oryza sativa subsp. japonica OX=39947 GN=A	ATPBM_ORYSJ	24	24	25
2.4	ATP synthase subunit beta, mitochondrial OS=Daucus carota OX=4039 GN=ATPB PE=3 SV=1	ATPBM_DAUCA		10	
3.1	ATP synthase subunit alpha, mitochondrial OS=Marchantia polymorpha OX=3197 GN=ATPA	ATPAM_MARPO	15	15	14
3.2	ATP synthase subunit alpha, chloroplastic OS=Oenothera argillicola OX=3940 GN=atpA PE=3	ATPA_OENAR (+1)	13	12	12
3.3	ATP synthase subunit alpha, chloroplastic OS=Spinacia oleracea OX=3562 GN=atpA PE=1 SV=	ATPA_SPIOL	10	11	11
3.4	ATP synthase subunit alpha, chloroplastic OS=Stigeoclonium helveticum OX=55999 GN=atp	ATPA_STIHE	5	5	
3.5	ATP synthase subunit alpha, mitochondrial OS=Nicotiana plumbaginifolia OX=4092 GN=ATI	ATPAM_NICPL	19	18	18
4.1	V-type proton ATPase catalytic subunit A OS=Daucus carota OX=4039 PE=2 SV=1	VATA_DAUCA	33	31	34
4.2	V-type proton ATPase catalytic subunit A (Fragment) OS=Zea mays OX=4577 PE=2 SV=1	VATA_MAIZE	25	24	24
4.3	V-type proton ATPase catalytic subunit A OS=Beta vulgaris OX=161934 PE=2 SV=1	VATA_BETVU	20	21	21
4.4	V-type proton ATPase catalytic subunit A OS=Arabidopsis thaliana OX=3702 GN=VHA-A PE=1	VATA_ARATH	26	24	26
4.5	V-type proton ATPase catalytic subunit A OS=Citrus unshiu OX=55188 PE=2 SV=1	VATA_CITUN	29		29
4.6	V-type proton ATPase catalytic subunit A OS=Gossypium hirsutum OX=3635 GN=CVA69.24 I	VATA_GOSHI	28	26	27
4.7	V-type proton ATPase catalytic subunit A (Fragment) OS=Hordeum vulgare OX=4513 PE=2 S\	VATA_HORVU		20	
5.1	V-type proton ATPase subunit B2 OS=Arabidopsis thaliana OX=3702 GN=VHA-B2 PE=1 SV=1	VATB2_ARATH	33	31	33
5.2	V-type proton ATPase subunit B 2 (Fragment) OS=Gossypium hirsutum OX=3635 PE=2 SV=1	VATB2_GOSHI	17	16	17
5.3	V-type proton ATPase subunit B3 OS=Arabidopsis thaliana OX=3702 GN=VHA-B3 PE=2 SV=1	VATB3_ARATH	28	27	28
6.1	Pyrophosphate-energized vacuolar membrane proton pump OS=Vigna radiata var. radiata O	AVP_VIGRR	17	16	17
6.2	Pyrophosphate-energized vacuolar membrane proton pump OS=Hordeum vulgare OX=4513	AVP_HORVU	15	14	15
6.3	Pyrophosphate-energized vacuolar membrane proton pump 1 OS=Arabidopsis thaliana OX=	AVP1_ARATH	15	13	15
7.1	Ribulose bisphosphate carboxylase/oxygenase activase, chloroplastic OS=Spinacia oleracea	RCA_SPIOL	6	7	7
7.2	Ribulose bisphosphate carboxylase/oxygenase activase 2, chloroplastic OS=Larrea tridentat	RCA2_LARTR	8	10	8
7.3	Ribulose bisphosphate carboxylase/oxygenase activase, chloroplastic OS=Vigna radiata var.	RCA_VIGRR	9	9	9
7.4	Ribulose bisphosphate carboxylase/oxygenase activase 1, chloroplastic OS=Nicotiana tabac	RCA1_TOBAC	12	12	11
7.5	Ribulose bisphosphate carboxylase/oxygenase activase, chloroplastic OS=Zea mays OX=457	RCA_MAIZE	6	6	5
7.6	Ribulose bisphosphate carboxylase/oxygenase activase, chloroplastic OS=Chlamydomonas r	RCA_CHLRE	3	2	2
8.1	Plasma membrane ATPase 2 (Fragment) OS=Solanum lycopersicum OX=4081 GN=LHA2 PE=3	PMA2_SOLLC	18	17	17
8.2	Plasma membrane ATPase 4 OS=Nicotiana plumbaginifolia OX=4092 GN=PMA4 PE=2 SV=1	PMA4_NICPL	19	19	21
8.3	ATPase 10, plasma membrane-type OS=Arabidopsis thaliana OX=3702 GN=AHA10 PE=2 SV=2	PMA10_ARATH	6	7	6
8.4	ATPase 4, plasma membrane-type OS=Arabidopsis thaliana OX=3702 GN=AHA4 PE=2 SV=2	PMA4_ARATH	21	20	21
8.5	ATPase 1, plasma membrane-type OS=Arabidopsis thaliana OX=3702 GN=AHA1 PE=1 SV=3	PMA1_ARATH	17	16	19
8.6	ATPase 9, plasma membrane-type OS=Arabidopsis thaliana OX=3702 GN=AHA9 PE=2 SV=2	PMA9_ARATH	12	11	11
8.7	Plasma membrane ATPase 3 OS=Nicotiana plumbaginifolia OX=4092 GN=PMA3 PE=1 SV=1	PMA3_NICPL	21	20	22
8.8	Plasma membrane ATPase OS=Triticum aestivum OX=4565 GN=ha1 PE=2 SV=1	PMA1_WHEAT	16	13	15

8.9	Plasma membrane ATPase 1 OS=Nicotiana plumbaginifolia OX=4092 GN=PMA1 PE=2 SV=1	PMA1_NICPL	19	20	
8.1	Plasma membrane ATPase OS=Oryza sativa subsp. japonica OX=39947 GN=Os04g0656100 P	PMA1_ORYSJ			22
9.1	ADP,ATP carrier protein, mitochondrial OS=Solanum tuberosum OX=4113 GN=ANT PE=2 SV	ADT1_SOLTU	17	17	16
9.2	ADP,ATP carrier protein 1, mitochondrial OS=Arabidopsis thaliana OX=3702 GN=AAC1 PE=1	ADT1_ARATH	19	19	18
9.3	ADP,ATP carrier protein 1, mitochondrial OS=Gossypium hirsutum OX=3635 GN=ANT1 PE=2	ADT1_GOSHI	18	18	18
9.4	ADP,ATP carrier protein 1, mitochondrial OS=Triticum aestivum OX=4565 GN=ANT-G1 PE=3	ADT1_WHEAT	19	19	19
10.1	ATP synthase subunit beta, chloroplastic OS=Gossypium hirsutum OX=3635 GN=atpB PE=3 S	ATPB_GOSHI	18	18	18
10.2	ATP synthase subunit beta, chloroplastic OS=Schisandra sphenanthera OX=13674 GN=atpB	ATPB_SCHSP	16	15	16
10.3	ATP synthase subunit beta, chloroplastic OS=Daucus carota OX=4039 GN=atpB PE=3 SV=1	ATPB_DAUCA	19	19	19
10.4	ATP synthase subunit beta, chloroplastic OS=Cycas taitungensis OX=54799 GN=atpB PE=3 S\	ATPB_CYCTA	10	10	10
10.5	ATP synthase subunit beta, chloroplastic OS=Asarum canadense OX=28498 GN=atpB PE=3 S\	ATPB_ASACA	15	15	15
10.6	ATP synthase subunit beta, chloroplastic OS=Coffea arabica OX=13443 GN=atpB PE=3 SV=1	ATPB_COFAR	16		16
10.7	ATP synthase subunit beta, chloroplastic OS=Manihot esculenta OX=3983 GN=atpB PE=3 SV=	ATPB_MANES		15	
11.1	Glyceraldehyde-3-phosphate dehydrogenase B, chloroplastic (Fragment) OS=Nicotiana taba	G3PB_TOBAC	17	16	15
11.2	Glyceraldehyde-3-phosphate dehydrogenase GAPA1, chloroplastic OS=Arabidopsis thaliana	G3PA1_ARATH	13	12	11
11.3	Glyceraldehyde-3-phosphate dehydrogenase A, chloroplastic (Fragment) OS=Nicotiana taba	G3PA_TOBAC	10	10	10
11.4	Glyceraldehyde-3-phosphate dehydrogenase B, chloroplastic OS=Pisum sativum OX=3888 G	G3PB_PEA	14	13	13
11.5	Glyceraldehyde-3-phosphate dehydrogenase A, chloroplastic OS=Chlamydomonas reinhard	G3PA_CHLRE	10	10	9
11.6	Glyceraldehyde-3-phosphate dehydrogenase A, chloroplastic (Fragment) OS=Coelastrella va	G3PA_COEVA	6	6	5
11.7	Glyceraldehyde-3-phosphate dehydrogenase A, chloroplastic OS=Zea mays OX=4577 GN=GA	G3PA_MAIZE	8		8
12.1	Polyubiquitin (Fragment) OS=Acetabularia peniculus OX=35862 PE=3 SV=2	UBIQP_ACEPE		6	
12.2	Ubiquitin-40S ribosomal protein S27a OS=Solanum lycopersicum OX=4081 GN=UBI3 PE=1 S	RS27A_SOLLC (+1)	9	10	11
12.3	Ubiquitin-40S ribosomal protein S27a-2 OS=Arabidopsis thaliana OX=3702 GN=RPS27AB PE	R27AB_ARATH (+1)	9	9	11
13.1	Actin-7 OS=Arabidopsis thaliana OX=3702 GN=ACT7 PE=1 SV=1	ACT7_ARATH	21	20	19
13.2	Actin-41 (Fragment) OS=Solanum lycopersicum OX=4081 PE=3 SV=1	ACT1_SOLLC	15	15	14
13.3	Actin-1 OS=Glycine max OX=3847 GN=SAC1 PE=3 SV=2	ACT1_SOYBN	8	8	8
13.4	Actin-3 OS=Pisum sativum OX=3888 PE=2 SV=1	ACT3_PEA	17	17	17
13.5	Actin-3 OS=Glycine max OX=3847 GN=SAC3 PE=3 SV=2	ACT3_SOYBN	15	14	14
13.6	Actin-104 (Fragment) OS=Nicotiana tabacum OX=4097 PE=3 SV=1	ACT7_TOBAC	16	16	
13.7	Actin-1 OS=Daucus carota OX=4039 PE=2 SV=1	ACT1_DAUCA			17
14.1	Heat shock 70 kDa protein 18 OS=Arabidopsis thaliana OX=3702 GN=HSP70-18 PE=2 SV=1	HSP7N_ARATH	18	19	19
14.2	Heat shock 70 kDa protein 3 OS=Arabidopsis thaliana OX=3702 GN=HSP70-3 PE=1 SV=1	HSP7C_ARATH	18	18	18
14.3	Chloroplast envelope membrane 70 kDa heat shock-related protein OS=Spinacia oleracea O)	HSP7E_SPIOL	17	19	17
14.4	Heat shock cognate 70 kDa protein 2 OS=Solanum lycopersicum OX=4081 GN=HSC-2 PE=2 S	HSP72_SOLLC	21	22	20
14.5	Heat shock 70 kDa protein OS=Zea mays OX=4577 GN=HSP70 PE=3 SV=2	HSP70_MAIZE	21	20	22

14.6	Heat shock cognate 70 kDa protein OS=Petunia hybrida OX=4102 GN=HSP70 PE=2 SV=1	HSP7C_PETHY		22	
15.1	NADH dehydrogenase [ubiquinone] iron-sulfur protein 2 OS=Arabidopsis thaliana OX=3702	NDUS2_ARATH	19	19	16
15.2	NADH dehydrogenase [ubiquinone] iron-sulfur protein 2 OS=Nicotiana sylvestris OX=4096 G	NDUS2_NICSY	9	8	7
16.1	V-type proton ATPase subunit E OS=Spinacia oleracea OX=3562 GN=VATE PE=2 SV=1	VATE_SPIOL	9	8	7
16.2	V-type proton ATPase subunit E2 OS=Arabidopsis thaliana OX=3702 GN=VHA-E2 PE=2 SV=1	VATE2_ARATH	4	3	4
16.3	V-type proton ATPase subunit E OS=Mesembryanthemum crystallinum OX=3544 GN=VATE P	VATE_MESCR	10	10	9
16.4	V-type proton ATPase subunit E OS=Citrus limon OX=2708 GN=VATE PE=2 SV=1	VATE_CITLI	14	12	13
16.5	V-type proton ATPase subunit E OS=Gossypium hirsutum OX=3635 GN=VATE PE=2 SV=1	VATE_GOSHI	10	9	
17.1	Chaperone protein ClpC1, chloroplastic OS=Oryza sativa subsp. japonica OX=39947 GN=CLP	CLPC1_ORYSJ	29	27	31
17.2	ATP-dependent Clp protease ATP-binding subunit ClpA homolog, chloroplastic (Fragment) C	CLPA_BRANA	20	19	22
17.3	Chaperone protein ClpC2, chloroplastic OS=Oryza sativa subsp. japonica OX=39947 GN=CLP	CLPC2_ORYSJ	23	23	25
17.4	ATP-dependent Clp protease ATP-binding subunit ClpA homolog CD4A, chloroplastic OS=So	CLPAA_SOLLC	26	24	28
18.1	Clathrin heavy chain 1 OS=Oryza sativa subsp. japonica OX=39947 GN=Os11g0104900 PE=3	CLH1_ORYSJ (+1)	26	27	29
18.2	Clathrin heavy chain 1 OS=Arabidopsis thaliana OX=3702 GN=CHC1 PE=1 SV=1	CLAH1_ARATH	22	23	26
19.1	Pleiotropic drug resistance protein 1 OS=Nicotiana tabacum OX=4097 GN=PDR1 PE=2 SV=1	PDR1_TOBAC	7	5	8
19.2	Pleiotropic drug resistance protein TUR2 OS=Spirodela polyrhiza OX=29656 GN=TUR2 PE=1	TUR2_SPIPO	8	8	8
19.3	ABC transporter G family member 42 OS=Oryza sativa subsp. japonica OX=39947 GN=ABCG4	AB42G_ORYSJ	9	7	7
19.4	ABC transporter G family member 52 OS=Oryza sativa subsp. japonica OX=39947 GN=ABCG5	AB52G_ORYSJ	2	2	2
19.5	ABC transporter G family member 43 OS=Oryza sativa subsp. japonica OX=39947 GN=ABCG4	AB43G_ORYSJ		6	7
19.6	ABC transporter G family member 36 OS=Arabidopsis thaliana OX=3702 GN=ABCG36 PE=1 S	AB36G_ARATH	8	7	6
19.7	ABC transporter G family member 40 OS=Arabidopsis thaliana OX=3702 GN=ABCG40 PE=1 S	AB40G_ARATH	5	4	4
19.8	ABC transporter G family member 37 OS=Oryza sativa subsp. japonica OX=39947 GN=ABCG3	AB37G_ORYSJ	10		10
19.9	ABC transporter G family member 39 OS=Oryza sativa subsp. japonica OX=39947 GN=ABCG3	AB39G_ORYSJ	10	12	10
19.10	ABC transporter G family member 29 OS=Arabidopsis thaliana OX=3702 GN=ABCG29 PE=2 S	AB29G_ARATH	8	5	6
19.11	ABC transporter G family member 44 OS=Oryza sativa subsp. japonica OX=39947 GN=ABCG4	AB44G_ORYSJ	9		
20.1	Tubulin beta-1 chain (Fragment) OS=Daucus carota OX=4039 GN=TUBB1 PE=1 SV=2	TBB1_DAUCA	9	10	10
20.2	Tubulin beta-8 chain OS=Oryza sativa subsp. japonica OX=39947 GN=TUBB8 PE=2 SV=1	TBB8_ORYSJ	14	15	13
20.3	Tubulin beta chain OS=Volvox carteri OX=3067 GN=TUBB1 PE=3 SV=1	TBB1_VOLCA (+3)		7	7
20.4	Tubulin beta-5 chain OS=Gossypium hirsutum OX=3635 PE=2 SV=1	TBB5_GOSHI	14	15	14
20.5	Tubulin beta-2 chain OS=Oryza sativa subsp. japonica OX=39947 GN=TUBB2 PE=2 SV=1	TBB2_ORYSJ	14	15	14
20.6	Tubulin beta-1 chain OS=Glycine max OX=3847 GN=TUBB1 PE=3 SV=1	TBB1_SOYBN	10	10	9
20.7	Tubulin beta-1 chain OS=Pisum sativum OX=3888 GN=TUBB1 PE=3 SV=1	TBB1_PEA	15	16	
20.8	Tubulin beta-2 chain OS=Daucus carota OX=4039 GN=TUBB2 PE=2 SV=1	TBB2_DAUCA		10	
20.9	Tubulin beta-4 chain OS=Oryza sativa subsp. japonica OX=39947 GN=TUBB4 PE=2 SV=1	TBB4_ORYSJ (+1)			14
20.10	Tubulin beta-9 chain OS=Gossypium hirsutum OX=3635 PE=2 SV=1	TBB9_GOSHI		14	

21	Alliin lyase (Fragment) OS=Allium cepa var. aggregatum OX=28911 PE=2 SV=1	ALLN_ALLCG	7	9	9
22.1	Elongation factor 1-alpha OS=Nicotiana tabacum OX=4097 PE=2 SV=1	EF1A_TOBAC	17	14	18
22.2	Elongation factor 1-alpha OS=Zea mays OX=4577 GN=EF1A PE=3 SV=1	EF1A_MAIZE	13	10	14
22.3	Elongation factor 1-alpha OS=Triticum aestivum OX=4565 GN=TEF1 PE=2 SV=1	EF1A_WHEAT	16	12	17
23	Probable aquaporin TIP-type RB7-5A OS=Nicotiana tabacum OX=4097 PE=2 SV=1	TIP1_TOBAC (+5)	2	2	2
24.1	Eukaryotic initiation factor 4A-3 OS=Arabidopsis thaliana OX=3702 GN=TIF4A-3 PE=1 SV=1	IF4A3_ARATH	15	13	12
24.2	Eukaryotic initiation factor 4A-15 OS=Nicotiana tabacum OX=4097 PE=2 SV=1	IF415_TOBAC	17	15	14
24.3	Eukaryotic initiation factor 4A-2 OS=Arabidopsis thaliana OX=3702 GN=TIF4A-2 PE=1 SV=1	IF4A2_ARATH	16	14	13
24.4	Eukaryotic initiation factor 4A-1 OS=Oryza sativa subsp. japonica OX=39947 GN=Os06g0701	IF4A1_ORYSJ	15	13	12
24.5	Eukaryotic initiation factor 4A-11 OS=Nicotiana tabacum OX=4097 PE=2 SV=1	IF411_TOBAC	17	13	
25.1	NADH dehydrogenase [ubiquinone] iron-sulfur protein 3 OS=Beta trigyna OX=19769 GN=NA	NDUS3_BETTR (+2)	14	13	13
25.2	NADH dehydrogenase [ubiquinone] iron-sulfur protein 3 OS=Solanum tuberosum OX=4113	NDUS3_SOLTU	11	11	10
26.1	Hypersensitive-induced response protein 1 OS=Arabidopsis thaliana OX=3702 GN=HIR1 PE=	HIR1_ARATH	8	8	8
26.2	Hypersensitive-induced response protein-like protein 2 OS=Oryza sativa subsp. japonica OX=	HIRL2_ORYSJ	5	6	6
26.3	Hypersensitive-induced reaction 1 protein OS=Capsicum annuum OX=4072 GN=HIR1 PE=1 S	HIR1_CAPAN	9	9	9
26.4	Hypersensitive-induced response protein-like protein 1 OS=Oryza sativa subsp. japonica OX=	HIRL1_ORYSJ	8	8	8
26.5	Hypersensitive-induced response protein 1 OS=Oryza sativa subsp. japonica OX=39947 GN=I	HIR1_ORYSJ	6		
27.1	Phosphoenolpyruvate carboxylase OS=Pisum sativum OX=3888 PE=2 SV=1	CAPP_PEA	10	6	10
27.2	Phosphoenolpyruvate carboxylase 2 OS=Sorghum bicolor OX=4558 PE=3 SV=1	CAPP2_SORBI	10	8	12
27.3	Phosphoenolpyruvate carboxylase 1 OS=Arabidopsis thaliana OX=3702 GN=PPC1 PE=1 SV=1	CAPP1_ARATH	13	11	14
27.4	Phosphoenolpyruvate carboxylase OS=Phaseolus vulgaris OX=3885 PE=2 SV=1	CAPP_PHAVU	10	8	9
27.5	Phosphoenolpyruvate carboxylase 1 OS=Mesembryanthemum crystallinum OX=3544 GN=P	CAPP1_MESCR	9		10
27.6	Phosphoenolpyruvate carboxylase, housekeeping isozyme OS=Saccharum hybrid OX=15819	CAPP1_SACHY	10		
27.7	Phosphoenolpyruvate carboxylase OS=Flaveria pringlei OX=4226 GN=PPCA1 PE=1 SV=1	CAPP1_FLAPR	11		
28	NADH dehydrogenase [ubiquinone] flavoprotein 1, mitochondrial OS=Arabidopsis thaliana (	NDUV1_ARATH	12	14	12
29.1	Probable aquaporin PIP1-5 OS=Arabidopsis thaliana OX=3702 GN=PIP1-5 PE=1 SV=2	PIP15_ARATH	4	4	4
29.2	Probable aquaporin PIP-type pTOM75 OS=Solanum lycopersicum OX=4081 PE=2 SV=1	PIP1_SOLLC	2	2	2
29.3	Probable aquaporin PIP1-4 OS=Arabidopsis thaliana OX=3702 GN=PIP1.4 PE=1 SV=1	PIP14_ARATH	3	3	3
30.1	Photosystem I P700 chlorophyll a apoprotein A2 OS=Zea mays OX=4577 GN=psaB PE=3 SV=2	PSAB_MAIZE	10	9	10
30.2	Photosystem I P700 chlorophyll a apoprotein A2 OS=Vitis vinifera OX=29760 GN=psaB PE=3	PSAB_VITVI	11	10	11
31.1	NADH dehydrogenase [ubiquinone] iron-sulfur protein 1, mitochondrial OS=Arabidopsis tha	NDUS1_ARATH	11	10	11
31.2	NADH dehydrogenase [ubiquinone] iron-sulfur protein 1, mitochondrial OS=Solanum tuber	NDUS1_SOLTU	10	9	11
32.1	Probable V-type proton ATPase subunit d OS=Oryza sativa subsp. japonica OX=39947 GN=Os	VA0D_ORYSJ	9	9	9
32.2	V-type proton ATPase subunit d2 OS=Arabidopsis thaliana OX=3702 GN=VHA-d2 PE=2 SV=1	VA0D2_ARATH	6	5	6
33.1	Photosystem I reaction center subunit II, chloroplastic OS=Cucumis sativus OX=3659 GN=ps	PSAD_CUCSA	7	8	7

33.2	Photosystem I reaction center subunit II, chloroplastic OS=Hordeum vulgare OX=4513 GN=	PSAD_HORVU	4	4	3
33.3	Photosystem I reaction center subunit II-2, chloroplastic OS=Arabidopsis thaliana OX=3702	PSAD2_ARATH	6	5	
34.1	Ras-related protein RABB1c OS=Arabidopsis thaliana OX=3702 GN=RABB1C PE=1 SV=1	RAB1C_ARATH	14	14	14
34.2	Ras-related protein RABB1a OS=Arabidopsis thaliana OX=3702 GN=RABB1A PE=2 SV=1	RAB1A_ARATH	4		
35.1	14-3-3-like protein C OS=Glycine max OX=3847 GN=GF14C PE=2 SV=1	1433C_SOYBN	4	4	3
35.2	14-3-3-like protein GF14 kappa OS=Arabidopsis thaliana OX=3702 GN=GRF8 PE=1 SV=2	14338_ARATH	3	3	4
35.3	14-3-3-like protein 16R OS=Solanum tuberosum OX=4113 PE=2 SV=1	14335_SOLTU (+1)	4	7	6
35.4	14-3-3-like protein GF14 nu OS=Arabidopsis thaliana OX=3702 GN=GRF7 PE=1 SV=1	14337_ARATH	4	6	6
35.5	14-3-3-like protein GF14 omicron OS=Arabidopsis thaliana OX=3702 GN=GRF11 PE=2 SV=2	14311_ARATH	2	2	2
35.6	14-3-3-like protein B OS=Nicotiana tabacum OX=4097 PE=2 SV=1	1433B_TOBAC		5	4
35.7	14-3-3-like protein (Fragment) OS=Spinacia oleracea OX=3562 PE=2 SV=1	1433_SPIOL	3		5
35.8	14-3-3-like protein OS=Lilium longiflorum OX=4690 PE=2 SV=1	1433_LILLO	5	7	7
35.9	14-3-3-like protein OS=Helianthus annuus OX=4232 PE=2 SV=1	1433_HELAN	5	8	6
35.10	14-3-3-like protein OS=Pisum sativum OX=3888 PE=2 SV=1	1433_PEA	4		6
35.11	14-3-3 protein 4 OS=Solanum lycopersicum OX=4081 GN=TFT4 PE=2 SV=1	14334_SOLLC		4	
36.1	Glyceraldehyde-3-phosphate dehydrogenase, cytosolic OS=Petunia hybrida OX=4102 GN=G	G3PC_PETHY	10	10	11
36.2	Glyceraldehyde-3-phosphate dehydrogenase, cytosolic OS=Antirrhinum majus OX=4151 GN	G3PC_ANTMA			11
36.3	Glyceraldehyde-3-phosphate dehydrogenase, cytosolic OS=Craterostigma plantagineum OX	G3PC_CRAPL			8
37.1	Probably inactive leucine-rich repeat receptor-like protein kinase At5g48380 OS=Arabidops	Y5838_ARATH	2	2	2
37.2	L-type lectin-domain containing receptor kinase IV.1 OS=Arabidopsis thaliana OX=3702 GN=	LRK41_ARATH	2	2	2
37.3	Mitogen-activated protein kinase homolog NTF4 OS=Nicotiana tabacum OX=4097 GN=NTF4	NTF4_TOBAC	3	2	3
37.4	Probable LRR receptor-like serine/threonine-protein kinase At1g07650 OS=Arabidopsis thal	Y1765_ARATH	4	3	2
37.5	Probable leucine-rich repeat receptor-like serine/threonine-protein kinase At3g14840 OS=A	Y3148_ARATH	3	2	2
37.6	Receptor-like cytoplasmic kinase 185 OS=Oryza sativa subsp. japonica OX=39947 GN=RLCK1	RK185_ORYSJ	2		
37.7	Probable receptor-like protein kinase At2g42960 OS=Arabidopsis thaliana OX=3702 GN=At2	Y2296_ARATH	3		
37.8	L-type lectin-domain containing receptor kinase IX.1 OS=Arabidopsis thaliana OX=3702 GN=	LRK91_ARATH	2	2	2
38.1	Pyruvate decarboxylase 2 OS=Nicotiana tabacum OX=4097 GN=PDC2 PE=2 SV=1	PDC2_TOBAC	3	3	2
38.2	Pyruvate decarboxylase 3 OS=Arabidopsis thaliana OX=3702 GN=PDC3 PE=2 SV=1	PDC3_ARATH	4	4	4
38.3	Pyruvate decarboxylase 1 OS=Oryza sativa subsp. indica OX=39946 GN=PDC1 PE=2 SV=1	PDC1_ORYSI (+1)	5	5	4
39.1	40S ribosomal protein S5 (Fragment) OS=Cicer arietinum OX=3827 GN=RPS5 PE=2 SV=1	RS5_CICAR	8	8	8
39.2	40S ribosomal protein S5 (Fragment) OS=Nicotiana plumbaginifolia OX=4092 GN=RPS5 PE=	RS5_NICPL	7	7	7
40.1	60S ribosomal protein L10 OS=Vitis riparia OX=96939 GN=RPL10 PE=2 SV=1	RL10_VITRI	10	10	11
40.2	60S ribosomal protein L10 (Fragment) OS=Nicotiana tabacum OX=4097 GN=RPL10 PE=2 SV=	RL10_TOBAC	4	4	4
41.1	Alliin lyase 2 OS=Allium sativum OX=4682 PE=1 SV=1	ALLN2_ALLSA	4	5	4
41.2	Alliin lyase 1 OS=Allium sativum OX=4682 PE=1 SV=1	ALLN1_ALLSA	5		6

42.1	NADH dehydrogenase [ubiquinone] iron-sulfur protein 8, mitochondrial OS=Solanum tuber	NDUS8_SOLTU	7	9	6
42.2	NADH dehydrogenase [ubiquinone] iron-sulfur protein 8, mitochondrial OS=Nicotiana taba	NDUS8_TOBAC		6	
43.1	Photosystem I P700 chlorophyll a apoprotein A1 OS=Agrostis stolonifera OX=63632 GN=psa	PSAA_AGRST (+6)	10	11	11
43.2	Photosystem I P700 chlorophyll a apoprotein A1 OS=Calycanthus floridus var. glaucus OX=2	PSAA_CALFG	7	8	9
44.1	60S ribosomal protein L18a-2 OS=Arabidopsis thaliana OX=3702 GN=RPL18AB PE=1 SV=2	R18A2_ARATH	6	6	7
44.2	60S ribosomal protein L18a-3 OS=Arabidopsis thaliana OX=3702 GN=RPL18AC PE=2 SV=1	R18A3_ARATH	4	4	5
45.1	Ras-related protein RABA1f OS=Arabidopsis thaliana OX=3702 GN=RABA1F PE=2 SV=1	RAA1F_ARATH	9	9	7
45.2	Ras-related protein YPT3 OS=Nicotiana plumbaginifolia OX=4092 GN=YPT3 PE=2 SV=1	YPT3_NICPL	7	6	7
45.3	Ras-related protein Rab11C OS=Nicotiana tabacum OX=4097 GN=RAB11C PE=2 SV=1	RB11C_TOBAC	4	3	3
45.4	Ras-related protein RABA2c OS=Arabidopsis thaliana OX=3702 GN=RABA2C PE=2 SV=4	RAA2C_ARATH		5	5
45.5	Ras-related protein Rab11B OS=Nicotiana tabacum OX=4097 GN=RAB11B PE=2 SV=1	RB11B_TOBAC			5
46.1	Heat shock protein 81-1 OS=Oryza sativa subsp. indica OX=39946 GN=HSP81-1 PE=2 SV=1	HSP81_ORYSI (+1)	14	12	13
46.2	Heat shock protein 90-2 OS=Arabidopsis thaliana OX=3702 GN=HSP90-2 PE=1 SV=1	HS902_ARATH	9	9	8
46.3	Heat shock cognate protein 80 OS=Solanum lycopersicum OX=4081 GN=HSC80 PE=2 SV=1	HSP80_SOLLC	12	12	12
47.1	40S ribosomal protein S4 OS=Gossypium hirsutum OX=3635 GN=RPS4 PE=2 SV=1	RS4_GOSHI	8	10	9
47.2	40S ribosomal protein S4 OS=Oryza sativa subsp. japonica OX=39947 GN=RPS4 PE=2 SV=3	RS4_ORYSJ	7	8	9
48.1	Heat shock 70 kDa protein BIP1 OS=Oryza sativa subsp. japonica OX=39947 GN=BIP1 PE=1 S'	BIP1_ORYSJ	13	12	13
48.2	Luminal-binding protein 5 OS=Nicotiana tabacum OX=4097 GN=BIP5 PE=2 SV=1	BIP5_TOBAC	9	10	8
49.1	Tubulin alpha-1 chain OS=Hordeum vulgare OX=4513 GN=TUBA1 PE=2 SV=1	TBA1_HORVU (+4)	7	8	7
49.2	Tubulin alpha-6 chain OS=Arabidopsis thaliana OX=3702 GN=TUBA6 PE=1 SV=1	TBA6_ARATH	9	9	9
50.1	60S ribosomal protein L8 OS=Solanum lycopersicum OX=4081 GN=RPL8 PE=2 SV=1	RL8_SOLLC	4	4	4
50.2	60S ribosomal protein L8-1 OS=Arabidopsis thaliana OX=3702 GN=RPL8A PE=1 SV=2	RL81_ARATH	4	4	4
51.1	ADP-ribosylation factor 2-A OS=Arabidopsis thaliana OX=3702 GN=ARF2-A PE=2 SV=2	ARF2A_ARATH (+2)	8	8	8
52.1	30S ribosomal protein S4, chloroplastic (Fragment) OS=Hymenocallis littoralis OX=59040 G	RR4_HYMLI	7	7	6
52.2	30S ribosomal protein S4, chloroplastic OS=Canalohypopterygium tamariscinum OX=9873!	RR4_CANTA	3	3	3
53	60S ribosomal protein L11 OS=Medicago sativa OX=3879 GN=RPL11 PE=2 SV=1	RL11_MEDSA	8	9	8
54.1	60S ribosomal protein L3 OS=Oryza sativa subsp. japonica OX=39947 GN=RPL3 PE=2 SV=2	RL3_ORYSJ	9	10	11
54.2	60S ribosomal protein L3-1 OS=Arabidopsis thaliana OX=3702 GN=ARP1 PE=1 SV=5	RL31_ARATH	9	9	10
54.3	60S ribosomal protein L3-2 OS=Arabidopsis thaliana OX=3702 GN=ARP2 PE=2 SV=4	RL32_ARATH	7	8	
55.1	RuBisCO large subunit-binding protein subunit beta, chloroplastic (Fragment) OS=Secale cer	RUBB_SECCE	8	8	9
55.2	RuBisCO large subunit-binding protein subunit beta, chloroplastic OS=Brassica napus OX=3	RUBB_BRANA	7	10	11
55.3	Chaperonin 60 subunit beta 3, chloroplastic OS=Arabidopsis thaliana OX=3702 GN=CPN60E	CPNB3_ARATH	9	9	13
55.4	RuBisCO large subunit-binding protein subunit beta, chloroplastic OS=Pisum sativum OX=3	RUBB_PEA	8	10	12
56	40S ribosomal protein S15 OS=Oryza sativa subsp. japonica OX=39947 GN=RPS15 PE=2 SV=2	RS15_ORYSJ	4	4	4
57	Mitochondrial-processing peptidase subunit alpha OS=Solanum tuberosum OX=4113 GN=M	MPPA_SOLTU	4	4	4

58	V-type proton ATPase subunit c1 OS=Arabidopsis thaliana OX=3702 GN=VHA-c1 PE=2 SV=1	VATL1_ARATH (+11)	2	2	
59.1	Phosphoglycerate kinase, chloroplastic OS=Nicotiana tabacum OX=4097 PE=2 SV=1	PGKH_TOBAC	9	8	8
59.2	Phosphoglycerate kinase, cytosolic OS=Nicotiana tabacum OX=4097 PE=2 SV=1	PGKY_TOBAC	7	6	6
59.3	Phosphoglycerate kinase 1, chloroplastic OS=Arabidopsis thaliana OX=3702 GN=PGK1 PE=1	PGKH1_ARATH	9	7	
60	60S ribosomal protein L7a-1 OS=Arabidopsis thaliana OX=3702 GN=RPL7AA PE=2 SV=2	RL7A1_ARATH (+1)	5	4	5
61.1	Elongation factor 2 OS=Arabidopsis thaliana OX=3702 GN=LOS1 PE=1 SV=1	EF2_ARATH	8	8	8
61.2	Elongation factor 2 OS=Beta vulgaris OX=161934 PE=2 SV=1	EF2_BETVU	9	7	10
62.1	5-methyltetrahydropteroyltriglutamate--homocysteine methyltransferase OS=Plectranthus	METE_PLESU	11	8	9
62.2	5-methyltetrahydropteroyltriglutamate--homocysteine methyltransferase 1 OS=Oryza sativa	METE1_ORYSJ	8	3	5
62.3	5-methyltetrahydropteroyltriglutamate--homocysteine methyltransferase OS=Mesembryan	METE_MESCR	9	6	6
62.4	5-methyltetrahydropteroyltriglutamate--homocysteine methyltransferase OS=Catharanthu	METE_CATRO	8	4	5
63.1	Catalase OS=Avicennia marina OX=82927 PE=2 SV=1	CATA_AVIMR		3	2
63.2	Catalase isozyme 2 OS=Ricinus communis OX=3988 GN=CAT2 PE=2 SV=1	CATA2_RICCO		4	3
63.3	Catalase isozyme 1 (Fragment) OS=Nicotiana plumbaginifolia OX=4092 GN=CAT1 PE=2 SV=1	CATA1_NICPL	3	6	5
63.4	Catalase isozyme 1 OS=Hordeum vulgare OX=4513 GN=CAT1 PE=2 SV=1	CATA1_HORVU (+4)	3	5	4
63.5	Catalase isozyme 1 OS=Ricinus communis OX=3988 GN=CAT1 PE=2 SV=2	CATA1_RICCO	3	6	
64.1	40S ribosomal protein S3a OS=Brassica campestris OX=3711 GN=RPS3A PE=2 SV=2	RS3A_BRACM	4	3	3
64.2	40S ribosomal protein S3a OS=Catharanthus roseus OX=4058 GN=RPS3A PE=2 SV=4	RS3A_CATRO	4	3	4
64.3	40S ribosomal protein S3a-1 OS=Vitis vinifera OX=29760 GN=GSVIVT00020038001 PE=3 SV	RS3A1_VITVI	2	2	2
65	40S ribosomal protein S6 OS=Asparagus officinalis OX=4686 GN=rps6 PE=2 SV=1	RS6_ASPOF	10	11	10
66.1	NADH dehydrogenase [ubiquinone] iron-sulfur protein 7, mitochondrial OS=Brassica olerac	NDUS7_BRAOL	7	7	7
66.2	NADH dehydrogenase [ubiquinone] iron-sulfur protein 7, mitochondrial OS=Arabidopsis tha	NDUS7_ARATH	6	6	7
67	60S ribosomal protein L23A OS=Fritillaria agrestis OX=64177 GN=RPL23A PE=2 SV=1	RL23A_FRIAG	9	8	8
68	Adenylate kinase 4 OS=Oryza sativa subsp. japonica OX=39947 GN=ADK-B PE=2 SV=1	KAD4_ORYSJ	8	8	9
69	Mitochondrial phosphate carrier protein 3, mitochondrial OS=Arabidopsis thaliana OX=370	MPCP3_ARATH	9	9	9
70	ATP-dependent zinc metalloprotease FTSH 1, chloroplastic OS=Arabidopsis thaliana OX=370	FTSH1_ARATH	15	13	14
71.1	40S ribosomal protein S8 OS=Zea mays OX=4577 GN=RPS8 PE=2 SV=2	RS8_MAIZE	8	8	7
71.2	40S ribosomal protein S8-2 OS=Arabidopsis thaliana OX=3702 GN=RPS8B PE=2 SV=1	RS82_ARATH	8	7	8
72.1	Ferredoxin--NADP reductase, chloroplastic OS=Vicia faba OX=3906 GN=PETH PE=2 SV=1	FENR_VICFA	3	3	3
72.2	Ferredoxin--NADP reductase, leaf isozyme 1, chloroplastic OS=Oryza sativa subsp. indica OX=	FENR1_ORYSI	5	5	5
72.3	Ferredoxin--NADP reductase, leaf isozyme 2, chloroplastic OS=Oryza sativa subsp. japonica C	FENR2_ORYSJ	4	4	4
73.1	Cytochrome c oxidase subunit 2 OS=Zea mays OX=4577 GN=COX2 PE=2 SV=4	COX2_MAIZE	4	3	3
73.2	Cytochrome c oxidase subunit 2 OS=Arabidopsis thaliana OX=3702 GN=COX2 PE=1 SV=2	COX2_ARATH	4	3	3
73.3	Cytochrome c oxidase subunit 2 OS=Beta vulgaris OX=161934 GN=COX2 PE=2 SV=1	COX2_BETVU			3
74.1	Photosystem II CP43 reaction center protein OS=Agrostis stolonifera OX=63632 GN=psbC PI	PSBC_AGRST (+20)	7	6	8

74.2	Photosystem II CP43 reaction center protein OS=Lemna minor OX=4472 GN=psbC PE=3 SV=1	PSBC_LEMMI	7	6	8
75.1	40S ribosomal protein S3-3 OS=Arabidopsis thaliana OX=3702 GN=RPS3C PE=1 SV=1	RS33_ARATH	6	6	7
75.2	40S ribosomal protein S3-2 OS=Arabidopsis thaliana OX=3702 GN=RPS3B PE=1 SV=1	RS32_ARATH		7	7
76	60S ribosomal protein L13a-1 OS=Arabidopsis thaliana OX=3702 GN=RPL13AA PE=2 SV=1	R13A1_ARATH	4	3	
77.1	60S ribosomal protein L10a-2 OS=Arabidopsis thaliana OX=3702 GN=RPL10AB PE=1 SV=1	R10A2_ARATH	4	3	4
77.2	60S ribosomal protein L10a-3 OS=Arabidopsis thaliana OX=3702 GN=RPL10AC PE=1 SV=1	R10A3_ARATH	2		2
78.1	40S ribosomal protein S14 OS=Zea mays OX=4577 PE=3 SV=1	RS141_MAIZE	5	5	5
78.2	40S ribosomal protein S14 OS=Chlamydomonas reinhardtii OX=3055 GN=RPS14 PE=3 SV=1	RS14_CHLRE		7	7
79	ATP synthase subunit gamma, mitochondrial OS=Ipomoea batatas OX=4120 GN=ATPC PE=1	ATPG3_IPOBA	5	5	5
80.1	Ras-related protein RABE1c OS=Arabidopsis thaliana OX=3702 GN=RABE1C PE=1 SV=1	RAE1C_ARATH (+1)	9	8	10
80.2	GTP-binding protein YPTC1 OS=Chlamydomonas reinhardtii OX=3055 GN=YPTC1 PE=3 SV=1	YPTC1_CHLRE			4
80.3	GTP-binding protein YPTM2 OS=Zea mays OX=4577 GN=YPTM2 PE=2 SV=1	YPTM2_MAIZE	6	6	4
80.4	Ras-related protein RABD2a OS=Arabidopsis thaliana OX=3702 GN=RABD2A PE=1 SV=3	RAD2A_ARATH	5	5	4
81.1	Photosystem II CP47 reaction center protein OS=Acorus americanus OX=263995 GN=psbB P	PSBB_ACOAM (+5)	7	6	7
81.2	Photosystem II CP47 reaction center protein OS=Lemna minor OX=4472 GN=psbB PE=3 SV=1	PSBB_LEMMI	7		7
82	40S ribosomal protein S13 OS=Glycine max OX=3847 GN=RPS13 PE=2 SV=1	RS13_SOYBN	8	8	8
83	Chlorophyll a-b binding protein 4, chloroplastic OS=Arabidopsis thaliana OX=3702 GN=LHC	CA4_ARATH	6	6	6
84	Cytochrome c1-2, heme protein, mitochondrial (Fragment) OS=Solanum tuberosum OX=41	CY12_SOLTU	5	5	5
85.1	Photosystem I iron-sulfur center OS=Liriodendron tulipifera OX=3415 GN=psaC PE=3 SV=1	PSAC_LIRTU	7	6	5
85.2	Photosystem I iron-sulfur center OS=Dioscorea elephantipes OX=145284 GN=psaC PE=3 SV=	PSAC_DIOEL	7	6	5
86	V-type proton ATPase subunit D OS=Arabidopsis thaliana OX=3702 GN=VHA-D PE=1 SV=2	VATD_ARATH	4	5	4
87.1	ATP-dependent zinc metalloprotease FTSH 8, chloroplastic OS=Arabidopsis thaliana OX=370	FTSH8_ARATH	7	7	7
87.2	ATP-dependent zinc metalloprotease FTSH 2, chloroplastic OS=Arabidopsis thaliana OX=370	FTSH2_ARATH	6	6	6
87.3	ATP-dependent zinc metalloprotease FTSH 2, chloroplastic OS=Oryza sativa subsp. japonica	FTSH2_ORYSJ	6	6	6
88	V-type proton ATPase subunit a3 OS=Arabidopsis thaliana OX=3702 GN=VHA-a3 PE=1 SV=1	VHAA3_ARATH	6	6	6
89	Hexokinase-1 OS=Arabidopsis thaliana OX=3702 GN=HXK1 PE=1 SV=2	HXK1_ARATH	5	5	5
90	50S ribosomal protein L2, chloroplastic OS=Triticum aestivum OX=4565 GN=rpl2-A PE=3 SV	RK2_WHEAT	7	6	7
91.1	60S ribosomal protein L13-1 OS=Brassica napus OX=3708 PE=2 SV=1	RL131_BRANA	5	5	4
91.2	60S ribosomal protein L13 OS=Nicotiana tabacum OX=4097 GN=RPL13 PE=2 SV=1	RL13_TOBAC		4	3
92	40S ribosomal protein S2-1 OS=Arabidopsis thaliana OX=3702 GN=RPS2A PE=2 SV=2	RS21_ARATH (+2)	5	5	5
93	40S ribosomal protein S15a-1 OS=Arabidopsis thaliana OX=3702 GN=RPS15AA PE=2 SV=2	R15A1_ARATH (+1)	5	5	5
94.1	60S ribosomal protein L7-2 OS=Arabidopsis thaliana OX=3702 GN=RPL7B PE=1 SV=1	RL72_ARATH	5	5	3
94.2	60S ribosomal protein L7-4 OS=Arabidopsis thaliana OX=3702 GN=RPL7D PE=2 SV=1	RL74_ARATH	6	6	4
95.1	40S ribosomal protein S16 OS=Fritillaria agrestis OX=64177 GN=RPS16 PE=2 SV=1	RS16_FRIAG	4	4	5
95.2	40S ribosomal protein S16 OS=Lupinus polyphyllus OX=3874 GN=RPS16 PE=2 SV=1	RS16_LUPPO	4	4	5

96.1	Adenosylhomocysteinase OS=Nicotiana sylvestris OX=4096 GN=SAHH PE=2 SV=1	SAHH_NICSY (+1)	10	7	9
96.2	Adenosylhomocysteinase OS=Medicago sativa OX=3879 GN=SAHH PE=2 SV=1	SAHH_MEDSA	10	7	8
97.1	Cell division cycle protein 48 homolog OS=Glycine max OX=3847 GN=CDC48 PE=2 SV=1	CDC48_SOYBN	8	9	9
97.2	Cell division control protein 48 homolog D OS=Arabidopsis thaliana OX=3702 GN=CDC48D	CD48D_ARATH	6	8	7
98	Probable mitochondrial-processing peptidase subunit beta, mitochondrial OS=Arabidopsis	MPPB_ARATH	5	4	4
99.1	Prohibitin-3, mitochondrial OS=Arabidopsis thaliana OX=3702 GN=PHB3 PE=1 SV=1	PHB3_ARATH	4	4	4
99.2	Prohibitin-5, mitochondrial OS=Arabidopsis thaliana OX=3702 GN=PHB5 PE=1 SV=1	PHB5_ARATH		3	3
100	60S ribosomal protein L14-1 OS=Arabidopsis thaliana OX=3702 GN=RPL14A PE=2 SV=1	RL141_ARATH	2	2	2
101.1	Elongation factor Tu, chloroplastic OS=Tetradesmus obliquus OX=3088 GN=tufA PE=3 SV=1	EFTU_TETOB	3	3	2
101.2	Elongation factor Tu, chloroplastic OS=Glycine max OX=3847 GN=TUFA PE=3 SV=1	EFTU1_SOYBN	8	7	6
102	Ribulose bisphosphate carboxylase large chain (Fragments) OS=Vitis sp. OX=3604 GN=rbcL P	RBL_VITSX	5	4	5
103	Photosystem II protein D1 OS=Acorus calamus OX=4465 GN=psbA PE=3 SV=1	PSBA_ACOCL (+25)	6	6	6
104.1	Succinate dehydrogenase [ubiquinone] flavoprotein subunit 1, mitochondrial OS=Arabidop	SDHA1_ARATH	7	7	7
104.2	Succinate dehydrogenase [ubiquinone] flavoprotein subunit 2, mitochondrial OS=Arabidop	SDHA2_ARATH	7		
105.1	Chlorophyll a-b binding protein, chloroplastic OS=Oryza sativa subsp. indica OX=39946 GN=	CB23_ORYSI (+1)	2	2	2
105.2	Chlorophyll a-b binding protein 91R, chloroplastic OS=Petunia sp. OX=4104 GN=CAB91R PE	CB25_PETSP	5	5	5
106	Ras-related protein Rab7 OS=Gossypium hirsutum OX=3635 GN=RAB7 PE=2 SV=1	RAB7_GOSHI	7	6	6
107.1	Enolase OS=Oryza sativa subsp. japonica OX=39947 GN=ENO1 PE=1 SV=2	ENO_ORYSJ	4	4	4
107.2	Enolase OS=Ricinus communis OX=3988 PE=2 SV=1	ENO_RICCO	4	4	4
107.3	Enolase 1 OS=Zea mays OX=4577 GN=ENO1 PE=2 SV=1	ENO1_MAIZE	5	4	5
108	40S ribosomal protein S11 OS=Zea mays OX=4577 GN=RPS11 PE=2 SV=1	RS11_MAIZE	6	5	7
109.1	40S ribosomal protein S9-2 OS=Arabidopsis thaliana OX=3702 GN=RPS9C PE=1 SV=1	RS92_ARATH	5	5	5
109.2	40S ribosomal protein S9-1 OS=Arabidopsis thaliana OX=3702 GN=RPS9B PE=1 SV=1	RS91_ARATH			4
110	Histone H4 variant TH011 OS=Triticum aestivum OX=4565 PE=3 SV=2	H41_WHEAT (+14)	6	7	7
111	30S ribosomal protein S7, chloroplastic OS=Allium textile OX=207935 GN=rps7 PE=3 SV=1	RR7_ALLTE	9	7	8
112.1	GTP-binding protein SAR1A OS=Arabidopsis thaliana OX=3702 GN=SAR1A PE=2 SV=1	SAR1A_ARATH	4	4	5
112.2	GTP-binding protein SAR1 OS=Nicotiana tabacum OX=4097 GN=SAR1 PE=2 SV=1	SAR1_TOBAC	4	4	5
113.1	Protein TIC 55, chloroplastic OS=Arabidopsis thaliana OX=3702 GN=TIC55 PE=1 SV=1	TIC55_ARATH	4	4	4
113.2	Protein TIC 55, chloroplastic OS=Pisum sativum OX=3888 GN=TIC55 PE=1 SV=1	TIC55_PEA	4	4	
114.1	Linoleate 9S-lipoxygenase A OS=Solanum lycopersicum OX=4081 GN=LOX1.1 PE=2 SV=1	LOXA_SOLLC	4	4	4
114.2	Linoleate 9S-lipoxygenase 1 OS=Arabidopsis thaliana OX=3702 GN=LOX1 PE=1 SV=1	LOX1_ARATH	2	2	2
114.3	Probable linoleate 9S-lipoxygenase 4 OS=Oryza sativa subsp. japonica OX=39947 GN=Os03g	LOX4_ORYSJ	3	3	4
115	Nucleoside diphosphate kinase IV, chloroplastic/mitochondrial OS=Arabidopsis thaliana OX	NDK4_ARATH	5	5	6
116.1	Oxygen-evolving enhancer protein 1-2, chloroplastic OS=Arabidopsis thaliana OX=3702 GN=	PSBO2_ARATH	2		
116.2	Oxygen-evolving enhancer protein 1, chloroplastic OS=Solanum tuberosum OX=4113 GN=P	PSBO_SOLTU	4	5	5

116.3	Oxygen-evolving enhancer protein 1, chloroplastic OS=Pisum sativum OX=3888 GN=PSBO P	PSBO_PEA	4	4	4
117	NADH dehydrogenase [ubiquinone] flavoprotein 2, mitochondrial OS=Arabidopsis thaliana (	NDUV2_ARATH	3	3	3
118	Histone H2B OS=Capsicum annuum OX=4072 GN=HIS2B PE=2 SV=3	H2B_CAPAN	4	2	3
119	Chlorophyll a-b binding protein 8, chloroplastic OS=Solanum lycopersicum OX=4081 GN=C	CB13_SOLLC	4	4	3
120.1	Chaperonin CPN60-like 1, mitochondrial OS=Arabidopsis thaliana OX=3702 GN=At2g3321C	CH60B_ARATH	6	5	6
120.2	Chaperonin CPN60-2, mitochondrial OS=Zea mays OX=4577 GN=CPN60II PE=2 SV=1	CH62_MAIZE	6	6	7
120.3	Chaperonin CPN60, mitochondrial OS=Arabidopsis thaliana OX=3702 GN=CPN60 PE=1 SV=2	CH60A_ARATH		5	6
121.1	RuBisCO large subunit-binding protein subunit alpha, chloroplastic OS=Pisum sativum OX=3	RUBA_PEA	7	7	7
121.2	RuBisCO large subunit-binding protein subunit alpha (Fragment) OS=Ricinus communis OX=	RUBA_RICCO	7	5	6
122	Ribulose bisphosphate carboxylase small chain, chloroplastic OS=Musa acuminata OX=4641	RBS_MUSAC	3	2	2
123.1	Calcium-dependent protein kinase 20 OS=Oryza sativa subsp. japonica OX=39947 GN=CPK2(	CDPKK_ORYSJ	7	5	6
123.2	Calcium-dependent protein kinase 32 OS=Arabidopsis thaliana OX=3702 GN=CPK32 PE=1 S\	CDPKW_ARATH	2	4	2
123.3	Calcium-dependent protein kinase 8 OS=Arabidopsis thaliana OX=3702 GN=CPK8 PE=1 SV=1	CDPK8_ARATH	3		
124.1	Pto-interacting protein 1 OS=Solanum lycopersicum OX=4081 GN=PTI1 PE=1 SV=2	PTI1_SOLLC	2	3	3
124.2	PTI1-like tyrosine-protein kinase 3 OS=Arabidopsis thaliana OX=3702 GN=PTI13 PE=1 SV=1	PTI13_ARATH			4
124.3	PTI1-like tyrosine-protein kinase 1 OS=Arabidopsis thaliana OX=3702 GN=PTI11 PE=1 SV=1	PTI11_ARATH		5	
125	Ras-related protein RABH1e OS=Arabidopsis thaliana OX=3702 GN=RABH1E PE=2 SV=1	RAH1E_ARATH	6	7	7
126	Probable V-type proton ATPase subunit H OS=Oryza sativa subsp. japonica OX=39947 GN=Os	VATH_ORYSJ	3	3	3
127	2-methyl-6-phytyl-1,4-hydroquinone methyltransferase 2, chloroplastic OS=Oryza sativa sul	BQMT2_ORYSJ			4
128	Calcineurin B-like protein 3 OS=Oryza sativa subsp. japonica OX=39947 GN=CBL3 PE=1 SV=1	CNBL3_ORYSJ	7	9	7
129.1	Sucrose synthase 1 OS=Oryza sativa subsp. japonica OX=39947 GN=SUS1 PE=1 SV=1	SUS1_ORYSJ	3	2	2
129.2	Sucrose synthase 1 OS=Tulipa gesneriana OX=13306 PE=2 SV=1	SUS1_TULGE	3		3
129.3	Sucrose synthase isoform 1 OS=Daucus carota OX=4039 PE=2 SV=1	SUS1_DAUCA	2	2	
129.4	Sucrose synthase OS=Vicia faba OX=3906 GN=SUCS PE=2 SV=1	SUS_VICFA		3	4
130	Calmodulin-3 OS=Oryza sativa subsp. indica OX=39946 GN=CAM3 PE=3 SV=2	CALM3_ORYSI (+3)	5	6	5
131	Glutamine synthetase root isozyme 4 OS=Zea mays OX=4577 GN=GLN5 PE=2 SV=1	GLNA4_MAIZE	3	4	4
132.1	Phototropin-2 OS=Arabidopsis thaliana OX=3702 GN=PHOT2 PE=1 SV=2	PHOT2_ARATH	4	4	3
132.2	Phototropin-1 OS=Arabidopsis thaliana OX=3702 GN=PHOT1 PE=1 SV=1	PHOT1_ARATH	5	4	4
133.1	26S proteasome regulatory subunit 7 homolog A OS=Arabidopsis thaliana OX=3702 GN=RPT	PRS7A_ARATH	5	6	7
133.2	26S proteasome regulatory subunit 7 OS=Spinacia oleracea OX=3562 GN=RPT1 PE=2 SV=1	PRS7_SPIOL	5		7
134	26S proteasome regulatory subunit 8 homolog A OS=Arabidopsis thaliana OX=3702 GN=RPT	PRS8A_ARATH (+1)	6	11	7
135	Putative 60S ribosomal protein L18-1 OS=Arabidopsis thaliana OX=3702 GN=RPL18A PE=3 S	RL181_ARATH	4	3	4
136.1	60S acidic ribosomal protein P0-3 OS=Arabidopsis thaliana OX=3702 GN=RPP0C PE=1 SV=1	RLA03_ARATH	6	5	6
136.2	60S acidic ribosomal protein P0-1 OS=Arabidopsis thaliana OX=3702 GN=RPP0A PE=1 SV=1	RLA01_ARATH	6	5	6
137.1	Cytochrome b-c1 complex subunit Rieske-2, mitochondrial OS=Arabidopsis thaliana OX=37	UCRI2_ARATH	5	4	5

137.2	Cytochrome b-c1 complex subunit Rieske-3, mitochondrial OS=Nicotiana tabacum OX=409	UCRI3_TOBAC	3	3	3
138	Prohibitin-2, mitochondrial OS=Arabidopsis thaliana OX=3702 GN=PHB2 PE=1 SV=1	PHB2_ARATH	3	3	2
139	Aquaporin PIP2-1 OS=Arabidopsis thaliana OX=3702 GN=PIP2-1 PE=1 SV=1	PIP21_ARATH	2	2	2
140.1	Ferredoxin-dependent glutamate synthase, chloroplastic OS=Spinacia oleracea OX=3562 GN	GLTB_SPIOL	4		3
140.2	Ferredoxin-dependent glutamate synthase 1, chloroplastic/mitochondrial OS=Arabidopsis t	GLTB1_ARATH	3	3	
141	Peptidyl-prolyl cis-trans isomerase OS=Allium cepa OX=4679 GN=CYP PE=2 SV=1	CYPH_ALLCE	3	3	3
142	Glycine dehydrogenase (decarboxylating) A, mitochondrial OS=Flaveria pringlei OX=4226 GI	GCSPA_FLAPR (+2)	3	3	4
143	Mitochondrial phosphate carrier protein 2, mitochondrial OS=Arabidopsis thaliana OX=370	MPCP2_ARATH	7	6	7
144	NADH dehydrogenase [ubiquinone] 1 alpha subcomplex subunit 9, mitochondrial OS=Arabi	NDUA9_ARATH	4	4	4
145.1	60S ribosomal protein L12-1 OS=Arabidopsis thaliana OX=3702 GN=RPL12A PE=2 SV=2	RL121_ARATH (+2)	2	2	2
145.2	60S ribosomal protein L12 OS=Prunus armeniaca OX=36596 GN=RPL12 PE=2 SV=1	RL12_PRUAR	2	2	2
146	60S ribosomal protein L23 OS=Arabidopsis thaliana OX=3702 GN=RPL23A PE=2 SV=3	RL23_ARATH	4	4	3
147	Cytochrome c1 2, heme protein, mitochondrial OS=Arabidopsis thaliana OX=3702 GN=CYC1	CYC1B_ARATH	4	4	4
148.1	30S ribosomal protein S3, chloroplastic OS=Dioscorea elephantipes OX=145284 GN=rps3 PI	RR3_DIOEL (+1)	3	4	3
148.2	30S ribosomal protein S3, chloroplastic OS=Calycanthus floridus var. glaucus OX=212734 G	RR3_CALFG	4	5	3
149	40S ribosomal protein S18 OS=Arabidopsis thaliana OX=3702 GN=RPS18A PE=1 SV=1	RS18_ARATH	3	4	5
150	60S ribosomal protein L17-1 OS=Hordeum vulgare OX=4513 PE=2 SV=1	RL171_HORVU	3	3	4
151	30S ribosomal protein S5, chloroplastic OS=Arabidopsis thaliana OX=3702 GN=rps5 PE=2 S\	RR5_ARATH	3	4	3
152	RuBisCO large subunit-binding protein subunit alpha, chloroplastic (Fragment) OS=Triticum	RUBA_WHEAT	6	5	6
153.1	Protein Ycf2 OS=Dioscorea elephantipes OX=145284 GN=ycf2-A PE=3 SV=1	YCF2_DIOEL	4	4	
153.2	Protein Ycf2 OS=Drimys granadensis OX=224735 GN=ycf2-A PE=3 SV=1	YCF2_DRIGR	3		
154.1	Coatomer subunit alpha-3 OS=Oryza sativa subsp. japonica OX=39947 GN=Os09g0127800 F	COPA3_ORYSJ	5	7	9
154.2	Coatomer subunit alpha-2 OS=Oryza sativa subsp. japonica OX=39947 GN=Os03g0711500 F	COPA2_ORYSJ	5	7	9
155.1	Phospholipase D alpha 2 OS=Oryza sativa subsp. japonica OX=39947 GN=PLD2 PE=2 SV=2	PLDA2_ORYSJ	2	3	4
155.2	Phospholipase D alpha 1 OS=Carica papaya OX=3649 GN=PLD1 PE=1 SV=1	PLDA1_CARPA		2	
156	26S proteasome regulatory subunit 6B homolog OS=Helianthus annuus OX=4232 PE=1 SV=1	PRS6B_HELAN	8	8	6
157	Protein TIC110, chloroplastic OS=Pisum sativum OX=3888 GN=TIC110 PE=1 SV=1	TI110_PEA	4	3	3
158	60S ribosomal protein L38 OS=Arabidopsis thaliana OX=3702 GN=RPL38A PE=3 SV=1	RL38_ARATH (+1)	2	3	2
159	Probable L-ascorbate peroxidase 4, peroxisomal OS=Oryza sativa subsp. japonica OX=39947	APX4_ORYSJ	4	4	4
160	NADP-dependent glyceraldehyde-3-phosphate dehydrogenase OS=Apium graveolens OX=40	GAPN_APIGR	5	4	5
161	Malate dehydrogenase, chloroplastic OS=Arabidopsis thaliana OX=3702 GN=At3g47520 PE=	MDHP_ARATH	5	4	4
162	60S ribosomal protein L19-2 OS=Arabidopsis thaliana OX=3702 GN=RPL19B PE=2 SV=1	RL192_ARATH	5	4	5
163.1	Rac-like GTP-binding protein RHO1 OS=Pisum sativum OX=3888 GN=RHO1 PE=2 SV=1	RHO1_PEA	3		3
163.2	Rac-like GTP-binding protein ARAC7 OS=Arabidopsis thaliana OX=3702 GN=ARAC7 PE=1 SV=	RAC7_ARATH	2		3
163.3	Rac-like GTP-binding protein RAC2 OS=Lotus japonicus OX=34305 GN=RAC2 PE=2 SV=1	RAC2_LOTJA	3		

164.1	Cellulose synthase A catalytic subunit 1 [UDP-forming] OS=Arabidopsis thaliana OX=3702 GI	CESA1_ARATH		2	3
164.2	Cellulose synthase A catalytic subunit 3 [UDP-forming] OS=Arabidopsis thaliana OX=3702 GI	CESA3_ARATH	2	2	
165	Glutamate--glyoxylate aminotransferase 1 OS=Arabidopsis thaliana OX=3702 GN=GGAT1 PE	GGT1_ARATH	6	6	5
166.1	Membrane steroid-binding protein 2 OS=Arabidopsis thaliana OX=3702 GN=MSBP2 PE=1 SV	MSBP2_ARATH	2	3	3
166.2	Membrane steroid-binding protein 1 OS=Oryza sativa subsp. japonica OX=39947 GN=MSBP1	MSBP1_ORYSJ	2	3	3
167	60S ribosomal protein L26-2 OS=Arabidopsis thaliana OX=3702 GN=RPL26B PE=2 SV=1	RL262_ARATH	5	5	6
168	Peroxisomal (S)-2-hydroxy-acid oxidase OS=Spinacia oleracea OX=3562 PE=1 SV=1	GOX_SPIOL	4	3	3
169.1	Acetyl-coenzyme A carboxylase carboxyl transferase subunit beta, chloroplastic OS=Illicium	ACCD_ILLOL	3	4	4
169.2	Acetyl-coenzyme A carboxylase carboxyl transferase subunit beta, chloroplastic OS=Phalaen	ACCD_PHAAO			2
170.1	Chaperone protein ClpB1 OS=Arabidopsis thaliana OX=3702 GN=CLPB1 PE=1 SV=2	CLPB1_ARATH	8	5	4
170.2	Chaperone protein ClpB1 OS=Oryza sativa subsp. japonica OX=39947 GN=CLPB1 PE=2 SV=1	CLPB1_ORYSJ		5	5
171	NADH-ubiquinone oxidoreductase chain 1 OS=Arabidopsis thaliana OX=3702 GN=ND1 PE=1	NU1M_ARATH	6	6	4
172	26S proteasome regulatory subunit 6A homolog B OS=Arabidopsis thaliana OX=3702 GN=RI	PS6AB_ARATH	8	6	7
173	Somatic embryogenesis receptor kinase 1 OS=Arabidopsis thaliana OX=3702 GN=SERK1 PE=	SERK1_ARATH (+1)	5	5	5
174	NADH-ubiquinone oxidoreductase 49 kDa subunit OS=Prototheca wickerhamii OX=3111 GI	NDUS2_PROWI	2	2	2
175.1	Membrane-associated 30 kDa protein, chloroplastic OS=Pisum sativum OX=3888 GN=IM30	IM30_PEA	4	4	5
175.2	Probable membrane-associated 30 kDa protein, chloroplastic OS=Oryza sativa subsp. japoni	IM30_ORYSJ	2	2	3
176.1	60S ribosomal protein L4-1 OS=Arabidopsis thaliana OX=3702 GN=RPL4A PE=1 SV=1	RL4A_ARATH	3	3	3
176.2	60S ribosomal protein L4 OS=Prunus armeniaca OX=36596 GN=RPL4 PE=2 SV=1	RL4_PRUAR	3	3	3
177.1	DNA-directed RNA polymerase subunit beta OS=Ipomoea purpurea OX=4121 GN=rpoB PE=3	RPOB_IPOPU	4	2	4
177.2	DNA-directed RNA polymerase subunit beta OS=Acorus americanus OX=263995 GN=rpoB PE	RPOB_ACOAM (+2)	4	3	5
177.3	DNA-directed RNA polymerase subunit beta OS=Lolium perenne OX=4522 GN=rpoB PE=3 SV	RPOB_LOLPR			2
178	40S ribosomal protein S17 OS=Solanum lycopersicum OX=4081 GN=RPS17 PE=2 SV=3	RS17_SOLLC	4	4	4
179.1	Phosphoinositide phosphatase SAC2 OS=Arabidopsis thaliana OX=3702 GN=SAC2 PE=2 SV=1	SAC2_ARATH	4	4	4
179.2	Phosphoinositide phosphatase SAC6 OS=Arabidopsis thaliana OX=3702 GN=SAC6 PE=2 SV=1	SAC6_ARATH			2
179.3	Phosphoinositide phosphatase SAC3 OS=Arabidopsis thaliana OX=3702 GN=SAC3 PE=2 SV=1	SAC3_ARATH	3		3
180	60S ribosomal protein L37a OS=Gossypium hirsutum OX=3635 GN=RPL37A PE=3 SV=1	RL37A_GOSHI	2	3	4
181.1	Calcium-dependent protein kinase 19 OS=Oryza sativa subsp. japonica OX=39947 GN=CPK1	CDPKJ_ORYSJ	4	5	4
181.2	Calcium-dependent protein kinase 9 OS=Arabidopsis thaliana OX=3702 GN=CPK9 PE=1 SV=1	CDPK9_ARATH	4	4	4
182	Dynamin-2B OS=Arabidopsis thaliana OX=3702 GN=DRP2B PE=1 SV=2	DRP2B_ARATH	7	6	6
183.1	Photosystem II D2 protein OS=Agrostis stolonifera OX=63632 GN=psbD PE=3 SV=1	PSBD_AGRST (+1)	6	6	6
183.2	Photosystem II D2 protein OS=Pisum sativum OX=3888 GN=psbD PE=1 SV=5	PSBD_PEA	6	6	6
184	26S proteasome non-ATPase regulatory subunit 1 homolog A OS=Arabidopsis thaliana OX=3	PSD1A_ARATH	5	4	6
185	26S proteasome non-ATPase regulatory subunit 2 homolog A OS=Arabidopsis thaliana OX=3	PSD2A_ARATH	4	4	5
186	Ras-related protein RABG3a OS=Arabidopsis thaliana OX=3702 GN=RABG3A PE=2 SV=1	RAG3A_ARATH	4	4	3

187.1	Transketolase, chloroplastic OS=Solanum tuberosum OX=4113 PE=2 SV=1	TKTC_SOLTU	5	4	3
187.2	Transketolase-2, chloroplastic OS=Arabidopsis thaliana OX=3702 GN=TKL-2 PE=1 SV=1	TKTC2_ARATH	2	2	2
187.3	Transketolase, chloroplastic OS=Zea mays OX=4577 PE=1 SV=1	TKTC_MAIZE		2	2
188	Probable receptor-like protein kinase At5g59700 OS=Arabidopsis thaliana OX=3702 GN=At5	Y5597_ARATH	2	2	2
189	30S ribosomal protein S11, chloroplastic OS=Nicotiana tomentosiformis OX=4098 GN=rps1	RR11_NICTO			3
190.1	Endoplasmin homolog OS=Hordeum vulgare OX=4513 PE=2 SV=1	ENPL_HORVU	5	4	6
190.2	Endoplasmin homolog OS=Catharanthus roseus OX=4058 GN=HSP90 PE=2 SV=1	ENPL_CATRO	6	5	6
190.3	Endoplasmin homolog OS=Arabidopsis thaliana OX=3702 GN=HSP90-7 PE=1 SV=1	ENPL_ARATH			6
191	Tripeptidyl-peptidase 2 OS=Oryza sativa subsp. japonica OX=39947 GN=TPP2 PE=2 SV=1	TPPII_ORYSJ	3	4	5
192	Mitochondrial dicarboxylate/tricarboxylate transporter DTC OS=Arabidopsis thaliana OX=3	DTC_ARATH	3	3	2
193	ADP-ribosylation factor-like protein 8a OS=Arabidopsis thaliana OX=3702 GN=ARL8A PE=2 S	ARL8A_ARATH (+1)	4	4	4
194	Bifunctional 6(G)-fructosyltransferase/2,1-fructan:2,1-fructan 1-fructosyltransferase OS=All	GFT_ALLCE	5	4	3
195	Malate dehydrogenase, cytoplasmic OS=Zea mays OX=4577 PE=1 SV=2	MDHC_MAIZE	4	4	3
196	60S ribosomal protein L34-3 OS=Arabidopsis thaliana OX=3702 GN=RPL34C PE=2 SV=1	RL343_ARATH	3	3	3
197.1	Glutamine synthetase leaf isozyme, chloroplastic OS=Phaseolus vulgaris OX=3885 PE=2 SV=1	GLNA4_PHAVU	2	2	2
197.2	Glutamine synthetase cytosolic isozyme 1-2 OS=Arabidopsis thaliana OX=3702 GN=GLN1-2 I	GLN12_ARATH	3	3	
197.3	Glutamine synthetase, chloroplastic/mitochondrial OS=Arabidopsis thaliana OX=3702 GN=	GLNA2_ARATH			2
198	Phosphoribulokinase, chloroplastic OS=Mesembryanthemum crystallinum OX=3544 PE=2 S	KPPR_MESCR	5	5	4
199.1	26S proteasome regulatory subunit 4 homolog OS=Oryza sativa subsp. japonica OX=39947 (	PRS4_ORYSJ	4	6	5
199.2	26S proteasome regulatory subunit 4 homolog B OS=Arabidopsis thaliana OX=3702 GN=RPT	PRS4B_ARATH	4	6	5
200	GTP-binding nuclear protein Ran-1 OS=Arabidopsis thaliana OX=3702 GN=RAN1 PE=1 SV=1	RAN1_ARATH (+6)	5	4	5
201	50S ribosomal protein L28, chloroplastic OS=Arabidopsis thaliana OX=3702 GN=RPL28 PE=:	RK28_ARATH	4	2	3
202	Elongation factor 1-gamma 1 OS=Oryza sativa subsp. japonica OX=39947 GN=Os02g022060	EF1G1_ORYSJ	2	3	2
203	Calnexin homolog OS=Helianthus tuberosus OX=4233 PE=2 SV=1	CALX_HELTU	4	4	4
204	Cytochrome c oxidase subunit 6b-1 OS=Arabidopsis thaliana OX=3702 GN=COX6B-1 PE=1 SV	CX6B1_ARATH	2	2	2
205.1	60S ribosomal protein L9-1 OS=Arabidopsis thaliana OX=3702 GN=RPL9B PE=1 SV=3	RL91_ARATH		2	2
205.2	60S ribosomal protein L9 OS=Pisum sativum OX=3888 GN=RPL9 PE=2 SV=1	RL9_PEA		2	2
206	Cytochrome b-c1 complex subunit 7 OS=Solanum tuberosum OX=4113 PE=1 SV=1	QCR7_SOLTU	2	2	2
207.1	Aminomethyltransferase, mitochondrial OS=Flaveria pringlei OX=4226 GN=GDCST PE=2 SV=	GCST_FLAPR	3	3	3
207.2	Aminomethyltransferase, mitochondrial OS=Solanum tuberosum OX=4113 GN=GDCST PE=2	GCST_SOLTU	2		
208	Nascent polypeptide-associated complex subunit alpha-like protein 3 OS=Arabidopsis thalia	NACA3_ARATH	4	4	4
209	26S proteasome regulatory subunit 10B homolog A OS=Arabidopsis thaliana OX=3702 GN=F	PS10A_ARATH	5	5	5
210	Protein RETICULATA-RELATED 4, chloroplastic OS=Arabidopsis thaliana OX=3702 GN=RER4 F	RER4_ARATH	3	3	3
211.1	DEAD-box ATP-dependent RNA helicase 52C OS=Oryza sativa subsp. japonica OX=39947 GN=	RH52C_ORYSJ	2	3	4
211.2	DEAD-box ATP-dependent RNA helicase 52B OS=Oryza sativa subsp. japonica OX=39947 GN=	RH52B_ORYSJ		2	2

211.3	DEAD-box ATP-dependent RNA helicase 37 OS=Arabidopsis thaliana OX=3702 GN=RH37 PE=:	RH37_ARATH	2	3	
212.1	60S ribosomal protein L24 OS=Prunus avium OX=42229 GN=RPL24 PE=2 SV=1	RL24_PRUAV	2	2	2
212.2	60S ribosomal protein L24 OS=Cicer arietinum OX=3827 GN=RPL24 PE=2 SV=1	RL24_CICAR	2	2	2
213	40S ribosomal protein S23 OS=Fragaria ananassa OX=3747 GN=RPS23 PE=2 SV=1	RS23_FRAAN	4	4	3
214.1	Protein TOC75-3, chloroplastic OS=Arabidopsis thaliana OX=3702 GN=TOC75-3 PE=1 SV=1	TC753_ARATH	3	4	4
214.2	Protein TOC75, chloroplastic OS=Oryza sativa subsp. japonica OX=39947 GN=TOC75 PE=2 S\	TOC75_ORYSJ	3	4	3
215.1	50S ribosomal protein L14, chloroplastic OS=Vitis vinifera OX=29760 GN=rpl14 PE=3 SV=1	RK14_VITVI	2	2	
215.2	50S ribosomal protein L14, chloroplastic OS=Ipomoea purpurea OX=4121 GN=rpl14 PE=3 S	RK14_IPOPU	2		
216	Thioredoxin-like protein CDSP32, chloroplastic OS=Arabidopsis thaliana OX=3702 GN=CDSI	CDSP_ARATH	2	2	2
217	50S ribosomal protein L16, chloroplastic OS=Calycanthus floridus var. glaucus OX=212734	RK16_CALFG	3	2	2
218	60S ribosomal protein L35-1 OS=Arabidopsis thaliana OX=3702 GN=RPL35A PE=2 SV=1	RL351_ARATH	3	2	2
219	60S ribosomal protein L18-2 OS=Arabidopsis thaliana OX=3702 GN=RPL18B PE=1 SV=2	RL182_ARATH	2	2	2
220	3-deoxy-manno-octulosonate cytidylyltransferase OS=Zea mays OX=4577 GN=KDSB PE=1 SV	KDSB_MAIZE		2	
221.1	Stromal 70 kDa heat shock-related protein, chloroplastic (Fragment) OS=Spinacia oleracea (	HSP7S_SPIOL	5		4
221.2	Heat shock 70 kDa protein 6, chloroplastic OS=Arabidopsis thaliana OX=3702 GN=HSP70-6 I	HSP7F_ARATH	5	4	4
222.1	Triosephosphate isomerase, cytosolic OS=Oryza sativa subsp. japonica OX=39947 GN=TPI PE	TPIS_ORYSJ	2	2	2
222.2	Triosephosphate isomerase, cytosolic OS=Zea mays OX=4577 PE=1 SV=3	TPIS_MAIZE	2	2	2
222.3	Triosephosphate isomerase, cytosolic OS=Secale cereale OX=4550 PE=2 SV=3	TPIS_SECCE		2	
223	Probable serine/threonine-protein kinase BSK3 OS=Oryza sativa subsp. japonica OX=39947 (	BSK3_ORYSJ	3	2	4
224.1	ABC transporter C family member 9 OS=Arabidopsis thaliana OX=3702 GN=ABCC9 PE=2 SV=2	AB9C_ARATH	2	2	2
224.2	ABC transporter C family member 5 OS=Arabidopsis thaliana OX=3702 GN=ABCC5 PE=2 SV=2	AB5C_ARATH		3	3
225	Probable NADH dehydrogenase [ubiquinone] 1 alpha subcomplex subunit 12 OS=Arabidops	NDUAC_ARATH	2	2	2
226	G-type lectin S-receptor-like serine/threonine-protein kinase At1g34300 OS=Arabidopsis th	Y1343_ARATH	4	2	
227	ATP synthase subunit d, mitochondrial OS=Arabidopsis thaliana OX=3702 GN=At3g52300 P	ATP5H_ARATH	4	4	5
228.1	Formate--tetrahydrofolate ligase OS=Arabidopsis thaliana OX=3702 GN=THFS PE=2 SV=1	FTHS_ARATH	3	2	2
228.2	Formate--tetrahydrofolate ligase OS=Spinacia oleracea OX=3562 PE=1 SV=3	FTHS_SPIOL	4	2	2
229.1	Malate dehydrogenase, glyoxysomal OS=Oryza sativa subsp. japonica OX=39947 GN=Os12g(	MDHG_ORYSJ	4	5	3
229.2	Malate dehydrogenase 2, glyoxysomal OS=Brassica napus OX=3708 GN=MDH2 PE=3 SV=1	MDHG2_BRANA (+1	4	4	3
230	S-adenosylmethionine synthase 1 OS=Populus trichocarpa OX=3694 GN=METK1 PE=2 SV=1	METK1_POPTR	3	4	4
231	NADH-ubiquinone oxidoreductase chain 2 OS=Arabidopsis thaliana OX=3702 GN=ND2 PE=2	NU2M_ARATH (+1)	3	2	4
232.1	Photosystem II 22 kDa protein, chloroplastic OS=Spinacia oleracea OX=3562 GN=PSBS PE=1	PSBS_SPIOL	4	4	3
232.2	Photosystem II 22 kDa protein 2, chloroplastic OS=Oryza sativa subsp. indica OX=39946 GN=	PSBS2_ORYSI (+1)	2	2	2
233	40S ribosomal protein SA OS=Vitis vinifera OX=29760 GN=GSVIVT00034021001 PE=3 SV=1	RSSA_VITVI	3	5	4
234	Cluster of NADH-ubiquinone oxidoreductase chain 4 OS=Arabidopsis thaliana OX=3702 GN=	NU4M_ARATH	3	3	3
234.1	NADH-ubiquinone oxidoreductase chain 4 OS=Arabidopsis thaliana OX=3702 GN=ND4 PE=2	NU4M_ARATH	3	3	3

235	60S ribosomal protein L31 OS=Perilla frutescens OX=48386 GN=RPL31 PE=2 SV=1	RL31_PERFR	3	3	3
236	50S ribosomal protein L15, chloroplastic (Fragment) OS=Pisum sativum OX=3888 GN=RPL1	RK15_PEA	3	4	3
237	Ras-related protein RABC1 OS=Arabidopsis thaliana OX=3702 GN=RABC1 PE=1 SV=1	RABC1_ARATH	5	4	5
238.1	ABC transporter C family member 12 OS=Arabidopsis thaliana OX=3702 GN=ABCC12 PE=2 SV	AB12C_ARATH	3	2	2
238.2	ABC transporter C family member 1 OS=Arabidopsis thaliana OX=3702 GN=ABCC1 PE=1 SV=1	AB1C_ARATH			4
239	Acetyl-coenzyme A carboxylase carboxyl transferase subunit alpha, chloroplastic OS=Arabid	ACCA_ARATH	3	2	
240	Serine hydroxymethyltransferase 1, mitochondrial OS=Arabidopsis thaliana OX=3702 GN=SI	GLYM1_ARATH	3	2	3
241	30S ribosomal protein S2, chloroplastic OS=Spinacia oleracea OX=3562 GN=rps2 PE=1 SV=1	RR2_SPIOL	2		2
242	Trans-cinnamate 4-monooxygenase OS=Petroselinum crispum OX=4043 GN=CYP73A10 PE=1	TCMO_PETCR	2		
243	ABC transporter B family member 25 OS=Oryza sativa subsp. japonica OX=39947 GN=ABCB2	AB25B_ORYSJ	4	4	4
244.1	ATP-dependent zinc metalloprotease FTSH 3, mitochondrial OS=Oryza sativa subsp. japonica	FTSH3_ORYSJ	3	4	4
244.2	ATP-dependent zinc metalloprotease FTSH 8, mitochondrial OS=Oryza sativa subsp. japonica	FTSH8_ORYSJ	4	3	3
245	NADP-dependent malic enzyme OS=Vitis vinifera OX=29760 PE=2 SV=1	MAOX_VITVI	3	3	3
246	26S proteasome non-ATPase regulatory subunit 11 homolog OS=Arabidopsis thaliana OX=37	PSD11_ARATH	3	5	3
247	Probable calcium-transporting ATPase 9, plasma membrane-type OS=Oryza sativa subsp. jap	ACA9_ORYSJ	4		3
248	Syntaxin-23 OS=Arabidopsis thaliana OX=3702 GN=SYP23 PE=1 SV=1	SYP23_ARATH	3	4	3
249.1	Gamma carbonic anhydrase 3, mitochondrial OS=Arabidopsis thaliana OX=3702 GN=GAMM	GCA3_ARATH	2	2	2
249.2	Gamma carbonic anhydrase 2, mitochondrial OS=Arabidopsis thaliana OX=3702 GN=GAMM	GCA2_ARATH	2		2
250	V-type proton ATPase subunit H OS=Arabidopsis thaliana OX=3702 GN=VHA-H PE=1 SV=1	VATH_ARATH	3	3	2
251.1	UTP--glucose-1-phosphate uridylyltransferase 1 OS=Arabidopsis thaliana OX=3702 GN=UGP1	UGPA1_ARATH	3		2
251.2	UTP--glucose-1-phosphate uridylyltransferase OS=Hordeum vulgare OX=4513 PE=2 SV=1	UGPA_HORVU		3	
252	ATP synthase gamma chain 1, chloroplastic OS=Arabidopsis thaliana OX=3702 GN=ATPC1 PE	ATPG1_ARATH	3		
253	ATP synthase epsilon chain, chloroplastic OS=Phalaenopsis aphrodite subsp. formosana OX=	ATPE_PHAAO	3	4	2
254	Sodium/calcium exchanger NCL1 OS=Oryza sativa subsp. japonica OX=39947 GN=NCL1 PE=2	NCL1_ORYSJ (+1)	2	2	2
255	Photosystem I reaction center subunit N, chloroplastic (Fragment) OS=Zea mays OX=4577 G	PSAN_MAIZE	5	5	4
256	Succinate dehydrogenase [ubiquinone] flavoprotein subunit, mitochondrial OS=Oryza sativa	SDHA_ORYSJ	5	7	6
257	ADP,ATP carrier protein 1, chloroplastic OS=Arabidopsis thaliana OX=3702 GN=AATP1 PE=1	TLC1_ARATH	3	2	3
258	Fructose-1,6-bisphosphatase 1, chloroplastic OS=Arabidopsis thaliana OX=3702 GN=CFBP1	F16P1_ARATH	4	3	5
259	Proline-rich receptor-like protein kinase PERK2 OS=Arabidopsis thaliana OX=3702 GN=PERK	PERK2_ARATH	3	3	3
260	Mitochondrial uncoupling protein 1 OS=Arabidopsis thaliana OX=3702 GN=PUMP1 PE=1 SV	PUMP1_ARATH	2	2	3
261	Triose phosphate/phosphate translocator, chloroplastic OS=Pisum sativum OX=3888 PE=1 S	TPT_PEA	4	3	2
262	Fructose-1,6-bisphosphatase, cytosolic OS=Brassica napus OX=3708 PE=2 SV=1	F16P2_BRANA	4	4	4
263	Obg-like ATPase 1 OS=Arabidopsis thaliana OX=3702 GN=YchF1 PE=1 SV=1	OLA1_ARATH	5	4	5
264	Ethanolamine-phosphate cytidylyltransferase OS=Arabidopsis thaliana OX=3702 GN=PECT1	PECT1_ARATH	3	4	3
265.1	Proteasome subunit alpha type-5 OS=Oryza sativa subsp. japonica OX=39947 GN=PAE1 PE=2	PSA5_ORYSJ	4	4	5

265.2	Proteasome subunit alpha type-5-A OS=Arabidopsis thaliana OX=3702 GN=PAE1 PE=1 SV=1	PSA5A_ARATH (+1)	4		
266	Vesicle-associated membrane protein 721 OS=Arabidopsis thaliana OX=3702 GN=VAMP721	VA721_ARATH (+1)	3	3	3
267	V-type proton ATPase subunit C OS=Hordeum vulgare OX=4513 GN=VATC PE=1 SV=1	VATC_HORVU	4	4	2
268	Cytochrome P450 97B1, chloroplastic OS=Pisum sativum OX=3888 GN=CYP97B1 PE=2 SV=1	C97B1_PEA	3	3	3
269.1	Isocitrate dehydrogenase [NAD] catalytic subunit 6, mitochondrial OS=Arabidopsis thaliana	IDH6_ARATH		2	2
269.2	3-isopropylmalate dehydrogenase, chloroplastic OS=Solanum tuberosum OX=4113 PE=2 SV	LEU3_SOLTU	2	3	3
270	Succinate dehydrogenase [ubiquinone] iron-sulfur subunit 1, mitochondrial OS=Oryza sativa	SDHB1_ORYSJ	2	2	
271	Delta(24)-sterol reductase OS=Arabidopsis thaliana OX=3702 GN=DIM PE=1 SV=2	DIM_ARATH	2	2	2
272	60S ribosomal protein L36 OS=Daucus carota OX=4039 GN=RPL36 PE=3 SV=2	RL36_DAUCA	2	2	2
273	Chlorophyll a-b binding protein, chloroplastic OS=Petunia hybrida OX=4102 PE=2 SV=1	CB12_PETHY	2		
274.1	Ras-related protein Rab5A OS=Oryza sativa subsp. japonica OX=39947 GN=RAB5A PE=1 SV=1	RAB5A_ORYSJ	2	2	2
274.2	Ras-related protein RHN1 OS=Nicotiana plumbaginifolia OX=4092 GN=RHN1 PE=2 SV=1	RHN1_NICPL	2		
275	Serine/threonine-protein kinase BSK6 OS=Arabidopsis thaliana OX=3702 GN=BSK6 PE=1 SV=	BSK6_ARATH	3	3	3
276	Chlorophyll a-b binding protein CP29.1, chloroplastic OS=Arabidopsis thaliana OX=3702 GI	CB4A_ARATH	2	2	
277	Phosphoinositide phospholipase C 7 OS=Arabidopsis thaliana OX=3702 GN=PLC7 PE=1 SV=1	PLCD7_ARATH			2
278	Phosphoinositide phospholipase C 3 OS=Arabidopsis thaliana OX=3702 GN=PLC3 PE=2 SV=1	PLCD3_ARATH	2	2	
279	Callose synthase 5 OS=Arabidopsis thaliana OX=3702 GN=CALS5 PE=1 SV=1	CALS5_ARATH			2
280	30S ribosomal protein S12, chloroplastic OS=Arabis hirsuta OX=78191 GN=rps12-A PE=3 SV	RR12_ARAHI	2		2
281	Serine/threonine-protein kinase BSK1 OS=Arabidopsis thaliana OX=3702 GN=BSK1 PE=1 SV=	BSK1_ARATH	2	3	4
282	Chlorophyll a-b binding protein CP26, chloroplastic OS=Arabidopsis thaliana OX=3702 GN=	CB5_ARATH	3	3	4
283	Casein kinase II subunit alpha-1 OS=Arabidopsis thaliana OX=3702 GN=CKA1 PE=1 SV=3	CSK21_ARATH	3	3	3
284.1	Glucose-6-phosphate 1-dehydrogenase, chloroplastic OS=Nicotiana tabacum OX=4097 PE=2	G6PDC_TOBAC		4	3
284.2	Glucose-6-phosphate 1-dehydrogenase, chloroplastic OS=Solanum tuberosum OX=4113 PE=	G6PDC_SOLTU	3		3
285	Photosystem II stability/assembly factor HCF136, chloroplastic OS=Oryza sativa subsp. japo	P2SAF_ORYSJ	4	5	2
286	50S ribosomal protein L17, chloroplastic OS=Spinacia oleracea OX=3562 GN=RPL17 PE=1 S\	RK17_SPIOL	3	4	4
287	Protein translocase subunit SecA, chloroplastic OS=Pisum sativum OX=3888 GN=SecA PE=1	SECA_PEA	5	4	4
288.1	Fructose-bisphosphate aldolase, cytoplasmic isozyme 2 OS=Pisum sativum OX=3888 PE=3 S\	ALF2_PEA	4	4	
288.2	Fructose-bisphosphate aldolase 8, cytosolic OS=Arabidopsis thaliana OX=3702 GN=FBA8 PE=	ALFC8_ARATH			6
289	Cysteine synthase OS=Brassica juncea OX=3707 PE=2 SV=1	CYSK2_BRAJU	3	2	3
290	Callose synthase 10 OS=Arabidopsis thaliana OX=3702 GN=CALS10 PE=2 SV=5	CALSA_ARATH	5	3	3
291	Carotenoid 9,10(9',10')-cleavage dioxygenase 1 OS=Phaseolus vulgaris OX=3885 GN=CCD1 P	CCD1_PHAVU	2	2	3
292	Probable methyltransferase PMT8 OS=Arabidopsis thaliana OX=3702 GN=At1g04430 PE=2 S	PMT8_ARATH	3	4	2
293	Protein TOC75, chloroplastic OS=Pisum sativum OX=3888 GN=TOC75 PE=1 SV=1	TOC75_PEA	3	5	5
294	ATP synthase delta chain, chloroplastic OS=Spinacia oleracea OX=3562 GN=ATPD PE=1 SV=2	ATPD_SPIOL	2	2	2
295	Cytochrome f OS=Citrus sinensis OX=2711 GN=petA PE=3 SV=1	CYF_CITSI	2	2	2

296	Cytochrome b559 subunit alpha OS=Amborella trichopoda OX=13333 GN=psbE PE=3 SV=3	PSBE_AMBTC (+15)	2	2	2
297	Guanosine nucleotide diphosphate dissociation inhibitor 1 OS=Arabidopsis thaliana OX=37	GDI1_ARATH	2	2	
298	NADH-cytochrome b5 reductase-like protein OS=Arabidopsis thaliana OX=3702 GN=CBR2 P	NB5R2_ARATH	2	2	
299	Protochlorophyllide reductase, chloroplastic OS=Pisum sativum OX=3888 GN=3PCR PE=1 S	POR_PEA	2		3
300	60S ribosomal protein L39-1 OS=Arabidopsis thaliana OX=3702 GN=RPL39A PE=3 SV=2	RL391_ARATH	2	2	2
301	Serine/threonine-protein phosphatase 2A 65 kDa regulatory subunit A gamma isoform OS=A	2AAG_ARATH	2	3	2
302	60S ribosomal protein L32-1 OS=Arabidopsis thaliana OX=3702 GN=RPL32A PE=2 SV=2	RL321_ARATH	2		2
303	Cyclin-dependent kinase A-2 OS=Oryza sativa subsp. japonica OX=39947 GN=CDKA-2 PE=2 S\	CDKA2_ORYSJ	3	4	
304	Biotin carboxylase 2, chloroplastic OS=Populus trichocarpa OX=3694 GN=POPTR_0018s14.	ACCC2_POPTR	4	4	3
305.1	Alcohol dehydrogenase OS=Fragaria ananassa OX=3747 GN=ADH PE=3 SV=2	ADH_FRAAN	3	4	3
305.2	Alcohol dehydrogenase OS=Malus domestica OX=3750 GN=ADH PE=2 SV=1	ADH_MALDO	2	3	2
305.3	Alcohol dehydrogenase 1 OS=Petunia hybrida OX=4102 GN=ADH1 PE=1 SV=1	ADH1_PETHY		3	2
306	Fructose-bisphosphate aldolase 5, cytosolic OS=Arabidopsis thaliana OX=3702 GN=FBA5 PE	ALFC5_ARATH	4	3	5
307	Fructose-bisphosphate aldolase 1, chloroplastic OS=Arabidopsis thaliana OX=3702 GN=FBA	ALFP1_ARATH	3	3	3
308	Probable inactive ATP-dependent zinc metalloprotease FTSHI 4, chloroplastic OS=Arabidops	FTSI4_ARATH	3	3	4
309	DnaJ homolog subfamily C GRV2 OS=Arabidopsis thaliana OX=3702 GN=GRV2 PE=1 SV=1	GRV2_ARATH		4	
310	DEAD-box ATP-dependent RNA helicase 15 OS=Oryza sativa subsp. japonica OX=39947 GN=A	RH15_ORYSJ (+1)	3	3	2
311	Receptor-like kinase TMK2 OS=Arabidopsis thaliana OX=3702 GN=TMK2 PE=1 SV=1	TMK2_ARATH	3	4	3
312.1	Serine/threonine-protein phosphatase BSL1 homolog OS=Oryza sativa subsp. japonica OX=3	BSL1_ORYSJ	2	2	3
312.2	Serine/threonine-protein phosphatase BSL2 OS=Arabidopsis thaliana OX=3702 GN=BSL2 PE	BSL2_ARATH	2		
313	Ubiquitin-conjugating enzyme E2 36 OS=Arabidopsis thaliana OX=3702 GN=UBC36 PE=1 SV	UBC36_ARATH	3	3	
314	Vesicle-fusing ATPase OS=Arabidopsis thaliana OX=3702 GN=NSF PE=2 SV=2	NSF_ARATH	3	4	2
315	Phytepsin OS=Hordeum vulgare OX=4513 PE=1 SV=1	ASPR_HORVU	3	3	3
316	30S ribosomal protein S10, chloroplastic OS=Mesembryanthemum crystallinum OX=3544 (	RR10_MESCR	2	2	2
317	Vacuolar protein sorting-associated protein 26A OS=Arabidopsis thaliana OX=3702 GN=VPS	VP26A_ARATH	2	3	3
318	Mitochondrial pyruvate carrier 2 OS=Arabidopsis thaliana OX=3702 GN=MPC2 PE=3 SV=1	MPC2_ARATH (+2)	2	2	2
319	60S ribosomal protein L23 OS=Syntrichia ruralis OX=38588 GN=RPL23 PE=2 SV=1	RL23_SYNRU	3	3	3
320	Galactolipid galactosyltransferase SFR2, chloroplastic OS=Arabidopsis thaliana OX=3702 GN	SFR2_ARATH	3	2	
321	Tripeptidyl-peptidase 2 OS=Arabidopsis thaliana OX=3702 GN=TPP2 PE=1 SV=1	TPPII_ARATH	2	2	2
322.1	Calcium-transporting ATPase 10, plasma membrane-type OS=Arabidopsis thaliana OX=3702	ACA10_ARATH	2		
322.2	Calcium-transporting ATPase 8, plasma membrane-type OS=Arabidopsis thaliana OX=3702 (	ACA8_ARATH	3		
323	40S ribosomal protein S10-2 OS=Arabidopsis thaliana OX=3702 GN=RPS10B PE=2 SV=1	RS102_ARATH	2	2	2
324	Heat shock protein 83 OS=Ipomoea nil OX=35883 GN=HSP83A PE=2 SV=1	HSP83_IPONI	4	3	6
325	Outer plastidial membrane protein porin OS=Pisum sativum OX=3888 GN=POR1 PE=1 SV=2	VDAC_PEA		2	
326	L-galactono-1,4-lactone dehydrogenase 2, mitochondrial OS=Oryza sativa subsp. japonica O	GLDH2_ORYSJ	2	2	2

327	Heat shock 70 kDa protein 9, mitochondrial OS=Arabidopsis thaliana OX=3702 GN=HSP70-9	HSP7I_ARATH		4	
328	2-Cys peroxiredoxin BAS1, chloroplastic OS=Oryza sativa subsp. japonica OX=39947 GN=BAS	BAS1_ORYSJ	2	3	2
329	Calcium-dependent protein kinase 29 OS=Arabidopsis thaliana OX=3702 GN=CPK29 PE=2 SV	CDPKT_ARATH	3	3	3
330	Hexokinase-7 OS=Oryza sativa subsp. japonica OX=39947 GN=HXK7 PE=2 SV=2	HXK7_ORYSJ	2	2	2
331	Succinate dehydrogenase subunit 5, mitochondrial OS=Arabidopsis thaliana OX=3702 GN=S	SDH5_ARATH	2	2	2
332	ABC transporter B family member 1 OS=Arabidopsis thaliana OX=3702 GN=ABCB1 PE=1 SV=1	AB1B_ARATH	2	4	3
333	Vacuolar protein sorting-associated protein 41 homolog OS=Solanum lycopersicum OX=408	VPS41_SOLLC	3	2	2
334	Aconitate hydratase 3, mitochondrial OS=Arabidopsis thaliana OX=3702 GN=ACO3 PE=1 SV=	ACO3M_ARATH		3	
335	L-ascorbate peroxidase 2, cytosolic OS=Oryza sativa subsp. japonica OX=39947 GN=APX2 PE	APX2_ORYSJ	2	2	2
336	ATP synthase protein MI25 OS=Triticum timopheevii OX=4570 PE=3 SV=1	MI25_TRITI (+1)	2	2	2
337	Carotenoid 9,10(9',10')-cleavage dioxygenase OS=Crocus sativus OX=82528 GN=CCD PE=1 SV	CCD_CROSA	3		3
338	ADP-ribosylation factor-like protein 8c OS=Arabidopsis thaliana OX=3702 GN=ARL8C PE=2 S	ARL8C_ARATH	2	2	2
339	Peroxisomal membrane protein 11-5 OS=Oryza sativa subsp. japonica OX=39947 GN=PEX11	PX115_ORYSJ	2	2	2
340	Casein kinase 1-like protein 1 OS=Arabidopsis thaliana OX=3702 GN=CKL1 PE=2 SV=2	CKL1_ARATH	3	3	
341	Magnesium-protoporphyrin IX monomethyl ester [oxidative] cyclase, chloroplastic OS=Goss	CRD1_GOSHI		4	
342	Histone H2A OS=Zea mays OX=4577 PE=2 SV=1	H2A_MAIZE	3		3
343	50S ribosomal protein L20, chloroplastic OS=Phalaenopsis aphrodite subsp. formosana OX=	RK20_PHAAO			2
344	Coatomer subunit alpha-2 OS=Arabidopsis thaliana OX=3702 GN=At2g21390 PE=2 SV=1	COPA2_ARATH	5	3	5
345	Coatomer subunit beta-1 OS=Arabidopsis thaliana OX=3702 GN=At4g31480 PE=3 SV=2	COPB1_ARATH (+1)	3	4	4
346	Peroxiredoxin Q, chloroplastic (Fragment) OS=Sedum lineare OX=114260 GN=PRXQ PE=1 SV	PERQ_SEDLI	4	3	4
347	Proteasome subunit beta type-6 OS=Arabidopsis thaliana OX=3702 GN=PBA1 PE=1 SV=2	PSB6_ARATH	3	3	2
348	Transmembrane 9 superfamily member 1 OS=Arabidopsis thaliana OX=3702 GN=TMN1 PE=1	TMN1_ARATH	3	2	3
349.1	30S ribosomal protein S1, chloroplastic OS=Spinacia oleracea OX=3562 GN=RPS1 PE=1 SV=1	RR1_SPIOL	3	2	3
349.2	30S ribosomal protein S1, chloroplastic OS=Arabidopsis thaliana OX=3702 GN=RPS1 PE=1 S	RPS1_ARATH		2	
350	Vacuolar protein sorting-associated protein 32 homolog 1 OS=Arabidopsis thaliana OX=370	VP321_ARATH	3	2	3
351	Glycerate dehydrogenase HPR, peroxisomal OS=Arabidopsis thaliana OX=3702 GN=HPR PE=	HPR1_ARATH	3	3	2
352	LysM domain receptor-like kinase 3 OS=Medicago truncatula OX=3880 GN=LYK3 PE=1 SV=1	LYK3_MEDTR	3	3	2
353	Photosystem I chlorophyll a/b-binding protein 2, chloroplastic OS=Arabidopsis thaliana OX	LHCA2_ARATH	2	2	2
354	30S ribosomal protein S11, chloroplastic OS=Adiantum capillus-veneris OX=13818 GN=rps1	RR11_ADICA	2	2	2
355.1	Probable UDP-arabinopyranose mutase 1 (Fragments) OS=Phoenix dactylifera OX=42345 PE	RGP1_PHODC		2	
355.2	Probable UDP-arabinopyranose mutase 1 OS=Solanum tuberosum OX=4113 GN=UPTG1 PE=	RGP1_SOLTU	2		2
356	Beta-glucosidase 1 OS=Oryza sativa subsp. japonica OX=39947 GN=BGLU1 PE=2 SV=1	BGL01_ORYSJ	3	3	4
357	Chlorophyll a-b binding protein 6A, chloroplastic OS=Solanum lycopersicum OX=4081 GN=	CB11_SOLLC	2	2	2
358	Cytochrome c OS=Allium porrum OX=4681 PE=1 SV=1	CYC_ALLPO	3	3	
359	30S ribosomal protein S19, chloroplastic OS=Picea abies OX=3329 GN=rps19 PE=3 SV=1	RR19_PICAB (+1)	2	2	2

360	RHOMBOID-like protein 6, mitochondrial OS=Arabidopsis thaliana OX=3702 GN=RBL6 PE=2	RBL6_ARATH	2	2	
361	Proteasome subunit beta type-5-B OS=Arabidopsis thaliana OX=3702 GN=PBE2 PE=1 SV=1	PSB5B_ARATH		2	2
362	Chloride channel protein CLC-a OS=Arabidopsis thaliana OX=3702 GN=CLC-A PE=1 SV=2	CLCA_ARATH		2	2
363	Non-structural maintenance of chromosomes element 4 homolog A OS=Arabidopsis thalian	NSE4A_ARATH	2	2	2
364	Allene oxide synthase 2, chloroplastic OS=Solanum lycopersicum OX=4081 GN=AOS2 PE=1 S	AOS2_SOLLC	2	2	
365	PLAT domain-containing protein 1 OS=Arabidopsis thaliana OX=3702 GN=PLAT1 PE=1 SV=1	PLAT1_ARATH		2	
366	Probable inactive ATP-dependent zinc metalloprotease FTSHI 5, chloroplastic OS=Arabidops	FTSI5_ARATH		2	
367	Pyrophosphate-energized membrane proton pump 2 OS=Arabidopsis thaliana OX=3702 GN	AVP2_ARATH	2	3	2
368	NADH dehydrogenase [ubiquinone] 1 beta subcomplex subunit 9 OS=Arabidopsis thaliana O	NDUB9_ARATH	3	2	3
369	Dihydroorotate dehydrogenase (quinone), mitochondrial OS=Oryza sativa subsp. japonica C	PYRD_ORYSJ	2	3	4
370	Ribonuclease J OS=Arabidopsis thaliana OX=3702 GN=RNJ PE=1 SV=1	RNJ_ARATH	2	4	2
371	Beta-adaptin-like protein C OS=Arabidopsis thaliana OX=3702 GN=BETAC-AD PE=1 SV=2	APBLC_ARATH	4	3	2
372	Serine/threonine-protein kinase BSK2 OS=Arabidopsis thaliana OX=3702 GN=BSK2 PE=1 SV=	BSK2_ARATH	3	4	
373	D-3-phosphoglycerate dehydrogenase 2, chloroplastic OS=Arabidopsis thaliana OX=3702 GI	SERA2_ARATH	3	3	2
374	Phosphoglucomutase, cytoplasmic OS=Pisum sativum OX=3888 GN=PGM1 PE=2 SV=1	PGMC_PEA	3	6	4
375.1	Serine/threonine-protein phosphatase PP2A catalytic subunit OS=Hevea brasiliensis OX=39	PP2A_HEVBR		2	3
375.2	Serine/threonine-protein phosphatase PP2A-3 catalytic subunit OS=Arabidopsis thaliana O	PP2A3_ARATH (+2)			3
376	Protein CURVATURE THYLAKOID 1A, chloroplastic OS=Arabidopsis thaliana OX=3702 GN=CU	CUT1A_ARATH		3	3
377	Glucose-6-phosphate 1-dehydrogenase, cytoplasmic isoform OS=Solanum tuberosum OX=4	G6PD_SOLTU	3	2	2
378.1	Malate dehydrogenase [NADP], chloroplastic OS=Zea mays OX=4577 PE=1 SV=1	MDHP_MAIZE	2	2	2
378.2	Malate dehydrogenase [NADP], chloroplastic OS=Flaveria bidentis OX=4224 PE=1 SV=1	MDHP_FLABI			2
379	Probable protein phosphatase 2C 33 OS=Oryza sativa subsp. japonica OX=39947 GN=Os03g	P2C33_ORYSJ	3	3	3
380	Importin subunit alpha-1b OS=Oryza sativa subsp. japonica OX=39947 GN=Os05g0155601 I	IMA1B_ORYSJ	2	2	2
381	Protein TIC 214 OS=Lemna minor OX=4472 GN=TIC214 PE=3 SV=1	TI214_LEMMI	3	2	3
382	Protein TIC 22, chloroplastic OS=Pisum sativum OX=3888 GN=TIC22 PE=1 SV=1	TIC22_PEA	3	2	2
383	Isocitrate dehydrogenase [NADP], chloroplastic (Fragment) OS=Medicago sativa OX=3879 PE	IDHP_MEDSA	3		3
384	Probable protein phosphatase 2C 9 OS=Arabidopsis thaliana OX=3702 GN=At1g22280 PE=1	P2C09_ARATH	3	3	2
385	Aspartic proteinase oryzasin-1 OS=Oryza sativa subsp. japonica OX=39947 GN=Os05g0567	ASPR1_ORYSJ	3	3	3
386	Cluster of Probable mediator of RNA polymerase II transcription subunit 36b OS=Arabidopsi	MD36B_ARATH [2]	3		
386.1	Probable mediator of RNA polymerase II transcription subunit 36b OS=Arabidopsis thaliana	MD36B_ARATH	2		
386.2	Mediator of RNA polymerase II transcription subunit 36a OS=Arabidopsis thaliana OX=3702	MD36A_ARATH	2		
387	Metal transporter Nramp2 OS=Oryza sativa subsp. japonica OX=39947 GN=NRAMP2 PE=2 SV	NRAM2_ORYSJ	2	2	
388	Proteasome subunit alpha type-7 OS=Cicer arietinum OX=3827 GN=PAD1 PE=2 SV=1	PSA7_CICAR	2	3	2
389	Brefeldin A-inhibited guanine nucleotide-exchange protein 3 OS=Arabidopsis thaliana OX=3	BIG3_ARATH	2		
390	Pyruvate dehydrogenase E1 component subunit beta-1, mitochondrial OS=Oryza sativa subs	ODPB1_ORYSJ	2	2	2

391	3-deoxy-manno-octulosonate cytidylyltransferase, mitochondrial OS=Arabidopsis thaliana (	KDSB_ARATH	2	2	2
392	40S ribosomal protein S7 OS=Hordeum vulgare OX=4513 GN=RPS7 PE=2 SV=1	RS7_HORVU	2	2	
393	Oligopeptide transporter 3 OS=Arabidopsis thaliana OX=3702 GN=OPT3 PE=2 SV=3	OPT3_ARATH	2	2	2
394	60S ribosomal protein L27a-3 OS=Arabidopsis thaliana OX=3702 GN=RPL27AC PE=2 SV=2	R27A3_ARATH	2		
395	26S proteasome non-ATPase regulatory subunit 6 homolog OS=Arabidopsis thaliana OX=37(	PSMD6_ARATH	3	3	2
396	Protein ROOT HAIR DEFECTIVE 3 OS=Oryza sativa subsp. japonica OX=39947 GN=RHD3 PE=2	RHD3_ORYSJ	3		2
397	Ras-related protein RABG2 OS=Arabidopsis thaliana OX=3702 GN=RABG2 PE=2 SV=2	RABG2_ARATH	2	2	2
398	Calnexin homolog OS=Glycine max OX=3847 PE=2 SV=1	CALX_SOYBN	4		4
399	Long chain acyl-CoA synthetase 9, chloroplastic OS=Arabidopsis thaliana OX=3702 GN=LACS	LACS9_ARATH	2	3	
400	26S proteasome non-ATPase regulatory subunit 6 OS=Oryza sativa subsp. japonica OX=3994	PSMD6_ORYSJ	4	3	2
401	Dihydroorotate dehydrogenase (quinone), mitochondrial OS=Arabidopsis thaliana OX=370	PYRD_ARATH	3	3	3
402	Sterol 3-beta-glucosyltransferase UGT80A2 OS=Arabidopsis thaliana OX=3702 GN=UGT80A2	U80A2_ARATH	3		2
403	Alcohol dehydrogenase class-3 OS=Pisum sativum OX=3888 PE=1 SV=1	ADHX_PEA	3	3	
404	40S ribosomal protein S27-1 OS=Arabidopsis thaliana OX=3702 GN=RPS27A PE=3 SV=1	RS271_ARATH (+3)	3	2	3
405	Bifunctional enolase 2/transcriptional activator OS=Arabidopsis thaliana OX=3702 GN=ENO	ENO2_ARATH	5	5	3
406	Glycerol-3-phosphate dehydrogenase SDP6, mitochondrial OS=Arabidopsis thaliana OX=37(	SDP6_ARATH	2	3	3
407	Coatomer subunit gamma-2 OS=Oryza sativa subsp. japonica OX=39947 GN=Os07g020110C	COPG2_ORYSJ	3	3	3
408	ATP synthase subunit epsilon, mitochondrial OS=Arabidopsis thaliana OX=3702 GN=At1g51	ATP5E_ARATH		2	
409	Monodehydroascorbate reductase, seedling isozyme OS=Cucumis sativus OX=3659 PE=2 SV=	MDARS_CUCSA	2	2	2
410	60S acidic ribosomal protein P2 OS=Parthenium argentatum OX=35935 PE=3 SV=1	RLA2_PARAR	2	2	2
411	Protein ACTIVITY OF BC1 COMPLEX KINASE 8, chloroplastic OS=Arabidopsis thaliana OX=370	AB1K8_ARATH	2	3	3
412	Bifunctional nitrilase/nitrile hydratase NIT4A OS=Nicotiana tabacum OX=4097 GN=NIT4A PI	NRL4A_TOBAC (+1)	2	2	2
413	External alternative NAD(P)H-ubiquinone oxidoreductase B4, mitochondrial OS=Arabidopsi	NDB4_ARATH	2	2	2
414	Probable solanesyl-diphosphate synthase 3, chloroplastic OS=Oryza sativa subsp. japonica C	SPS3_ORYSJ	2	3	3
415	40S ribosomal protein Sa-1 OS=Arabidopsis thaliana OX=3702 GN=RPSaA PE=1 SV=3	RSSA1_ARATH (+1)	3	4	2
416.1	DNA-directed RNA polymerase subunit alpha OS=Ceratophyllum demersum OX=4428 GN=rp	RPOA_CERDE		2	2
416.2	DNA-directed RNA polymerase subunit alpha OS=Drimys granadensis OX=224735 GN=rpoA I	RPOA_DRIGR		2	
417	50S ribosomal protein L13, chloroplastic OS=Spinacia oleracea OX=3562 GN=RPL13 PE=1 S\	RK13_SPIOL	2	2	2
418	Proteasome subunit alpha type-4 OS=Petunia hybrida OX=4102 GN=PAC1 PE=2 SV=1	PSA4_PETHY			2
419	Long chain base biosynthesis protein 2a OS=Oryza sativa subsp. japonica OX=39947 GN=Os1	LCB2A_ORYSJ		2	3
420	DNA-directed RNA polymerase subunit beta" OS=Glycine max OX=3847 GN=rpoC2 PE=3 SV=:	RPOC2_SOYBN		2	
421	Calcium-dependent protein kinase 1 OS=Oryza sativa subsp. japonica OX=39947 GN=CPK1 P	CDPK1_ORYSJ	3	3	3
422	Fe-S cluster assembly factor HCF101, chloroplastic OS=Arabidopsis thaliana OX=3702 GN=H	HF101_ARATH	2		
423	Long chain acyl-CoA synthetase 4 OS=Arabidopsis thaliana OX=3702 GN=LACS4 PE=2 SV=1	LACS4_ARATH			2
424	Histone H2A.2.1 OS=Triticum aestivum OX=4565 PE=1 SV=1	H2A2_WHEAT	2	2	2

425	Glutamine synthetase cytosolic isozyme 1-5 OS=Arabidopsis thaliana OX=3702 GN=GLN1-5 I	GLN15_ARATH			2
426	30S ribosomal protein S14, chloroplastic OS=Cucumis sativus OX=3659 GN=rps14 PE=3 SV=	RR14_CUCSA			2
427	6-phosphogluconate dehydrogenase, decarboxylating 2 OS=Arabidopsis thaliana OX=3702 (	6PGD2_ARATH		3	
428	Serine hydroxymethyltransferase 4 OS=Arabidopsis thaliana OX=3702 GN=SHM4 PE=1 SV=1	GLYC4_ARATH	3	2	2
429	GDP-mannose 3,5-epimerase OS=Arabidopsis thaliana OX=3702 GN=At5g28840 PE=1 SV=1	GME_ARATH	2	3	
430	Xylose isomerase OS=Hordeum vulgare OX=4513 GN=XYLA PE=1 SV=1	XYLA_HORVU	3	4	3
431	Putative coatomer subunit beta'-3 OS=Oryza sativa subsp. japonica OX=39947 GN=Os02g02	COB23_ORYSJ	3	2	5
432	Transmembrane 9 superfamily member 6 OS=Arabidopsis thaliana OX=3702 GN=TMN6 PE=2	TMN6_ARATH	3		3
433.1	Pantothenate kinase 2 OS=Arabidopsis thaliana OX=3702 GN=PANK2 PE=1 SV=2	PANK2_ARATH	2	2	2
433.2	Pantothenate kinase 2 OS=Oryza sativa subsp. japonica OX=39947 GN=Os09g0533100 PE=2	PANK2_ORYSJ	2	2	
434	Callose synthase 12 OS=Arabidopsis thaliana OX=3702 GN=CALS12 PE=2 SV=1	CALSC_ARATH	2	4	
435	UPF0603 protein OsI_019212, chloroplastic OS=Oryza sativa subsp. indica OX=39946 GN=C	U603_ORYSI (+1)	2	2	2
436	Ketol-acid reductoisomerase, chloroplastic OS=Arabidopsis thaliana OX=3702 GN=At3g586	ILV5_ARATH	3	3	3
437	Coatomer subunit beta-2 OS=Oryza sativa subsp. japonica OX=39947 GN=Os01g0281400 PE	COPB2_ORYSJ		4	2
438	ATP-dependent zinc metalloprotease FTSH 4, mitochondrial OS=Arabidopsis thaliana OX=37	FTSH4_ARATH	3	2	
439	DnaJ protein homolog 1 (Fragment) OS=Allium porrum OX=4681 GN=DNAJ1 PE=2 SV=1	DNJH1_ALLPO			2
440	Histone H1-I OS=Volvox carteri OX=3067 GN=H1-I PE=2 SV=3	H11_VOLCA			2
441	ABC transporter G family member 22 OS=Arabidopsis thaliana OX=3702 GN=ABCG22 PE=1 S	AB22G_ARATH	2		2
442	Dihydrolipoyllysine-residue acetyltransferase component 2 of pyruvate dehydrogenase com	ODP22_ARATH	2	2	
443	SPX domain-containing membrane protein OsI_21475 OS=Oryza sativa subsp. indica OX=39	SPXM3_ORYSI (+1)	3		2
444	26S proteasome non-ATPase regulatory subunit 12 homolog B OS=Arabidopsis thaliana OX=	PS12B_ARATH	2		
445	Dolichyl-diphosphooligosaccharide--protein glycosyltransferase 48 kDa subunit OS=Oryza s	OST48_ORYSJ	2	2	2
446	Proteasome subunit alpha type-6 OS=Nicotiana tabacum OX=4097 GN=PAA1 PE=2 SV=1	PSA6_TOBAC		2	2
447	Elongation factor G, chloroplastic OS=Arabidopsis thaliana OX=3702 GN=CPEFG PE=1 SV=1	EFGC_ARATH	2	3	
448	ATP-dependent 6-phosphofructokinase 7 OS=Arabidopsis thaliana OX=3702 GN=PFK7 PE=1	PFKA7_ARATH	2		
449	Protein CHUP1, chloroplastic OS=Arabidopsis thaliana OX=3702 GN=CHUP1 PE=1 SV=1	CHUP1_ARATH		2	2
450	Pyruvate kinase 1, cytosolic OS=Oryza sativa subsp. indica OX=39946 GN=OsI_35105 PE=3 S	KPYC1_ORYSI (+1)		2	
451	Ubiquinol oxidase 1a, mitochondrial OS=Oryza sativa subsp. japonica OX=39947 GN=AOX1A	AOX1A_ORYSJ	2	2	
452	NADH-ubiquinone oxidoreductase chain 5 OS=Marchantia polymorpha OX=3197 GN=ND5 F	NU5M_MARPO	2		
453	Flotillin-like protein 1 OS=Arabidopsis thaliana OX=3702 GN=FLOT1 PE=2 SV=1	FLOT1_ARATH		2	2
454	ABC transporter C family member 14 OS=Arabidopsis thaliana OX=3702 GN=ABCC14 PE=1 S	AB14C_ARATH	2		3
455	6-phosphofructo-2-kinase/fructose-2,6-bisphosphatase OS=Arabidopsis thaliana OX=3702 (	F26_ARATH	3	2	2
456	ABC transporter G family member 7 OS=Arabidopsis thaliana OX=3702 GN=ABCG7 PE=2 SV=	AB7G_ARATH	3	3	3
457	Cyprosin (Fragment) OS=Cynara cardunculus OX=4265 GN=CYPRO1 PE=1 SV=2	CYPR1_CYNCA	2		2
458	Probable calcium-binding protein CML13 OS=Arabidopsis thaliana OX=3702 GN=CML13 PE	CML13_ARATH	2	2	2

459	Heat shock 70 kDa protein 15 OS=Arabidopsis thaliana OX=3702 GN=HSP70-15 PE=1 SV=1	HSP7P_ARATH		2	2
460	Nucleoside diphosphate kinase B OS=Flaveria bidentis OX=4224 PE=2 SV=1	NDKB_FLABI	2	2	2
461	Prohibitin-7, mitochondrial OS=Arabidopsis thaliana OX=3702 GN=PHB7 PE=1 SV=1	PHB7_ARATH	2	2	2
462	Photosystem I reaction center subunit VI-2, chloroplastic OS=Arabidopsis thaliana OX=370	PSAH2_ARATH	2	2	2
463	40S ribosomal protein S30 OS=Arabidopsis thaliana OX=3702 GN=RPS30A PE=3 SV=3	RS30_ARATH	2	2	2
464	Cytochrome b6-f complex iron-sulfur subunit, chloroplastic OS=Fritillaria agrestis OX=6417	UCRIA_FRIAG	2	2	2
465	Eukaryotic peptide chain release factor subunit 1-1 OS=Arabidopsis thaliana OX=3702 GN=E	ERF1X_ARATH	2	2	
466	Vacuolar protein-sorting-associated protein 11 homolog OS=Arabidopsis thaliana OX=3702	VPS11_ARATH	2		2
467	Dynamin-related protein 1A OS=Arabidopsis thaliana OX=3702 GN=DRP1A PE=1 SV=3	DRP1A_ARATH	2	3	2
468	HMG1/2-like protein OS=Ipomoea nil OX=35883 PE=2 SV=1	HMGL_IPONI	2	2	
469	Vacuolar-sorting receptor 2 OS=Arabidopsis thaliana OX=3702 GN=VSR2 PE=2 SV=1	VSR2_ARATH	3	3	2
470	Thioredoxin H4-1 OS=Oryza sativa subsp. japonica OX=39947 GN=Os01g0168200 PE=2 SV=1	TRH41_ORYSJ (+1)	2	2	
471	Cytochrome c1-1, heme protein, mitochondrial OS=Solanum tuberosum OX=4113 GN=CYC	CY11_SOLTU	4	4	5
472	Glucan endo-1,3-beta-glucosidase 5 OS=Arabidopsis thaliana OX=3702 GN=At4g31140 PE=	E135_ARATH	2		2
473	Fructose-1,6-bisphosphatase, cytosolic OS=Oryza coarctata OX=77588 PE=2 SV=1	F16P2_ORYCO	2	3	2
474	Calcium-transporting ATPase 1, endoplasmic reticulum-type OS=Arabidopsis thaliana OX=3	ECA1_ARATH (+1)	2	2	2
475	Phytochrome-associated serine/threonine-protein phosphatase 1 OS=Arabidopsis thaliana	FYPP1_ARATH (+1)	2	2	
476	ABC transporter B family member 25, mitochondrial OS=Arabidopsis thaliana OX=3702 GN=	AB25B_ARATH	2	2	2
477	Spermidine synthase 1 OS=Pisum sativum OX=3888 GN=SPDSYN1 PE=2 SV=1	SPD1_PEA	2		
478	Peroxisomal isocitrate dehydrogenase [NADP] OS=Arabidopsis thaliana OX=3702 GN=ICDH	ICDHX_ARATH		2	3
479	Heat shock protein 90-5, chloroplastic OS=Arabidopsis thaliana OX=3702 GN=HSP90-5 PE=	HS905_ARATH	2		
480	8-hydroxygeraniol oxidoreductase OS=Catharanthus roseus OX=4058 GN=8HGO PE=1 SV=1	8HGO_CATRO	2		
481	Proline--tRNA ligase, cytoplasmic OS=Arabidopsis thaliana OX=3702 GN=At3g62120 PE=1 S	SYPC_ARATH			2
482	Magnesium-chelatase subunit ChlI-1, chloroplastic OS=Arabidopsis thaliana OX=3702 GN=C	CHLI1_ARATH	2	2	2
483	Dynamin-related protein 12A OS=Glycine max OX=3847 PE=1 SV=1	SDLCA_SOYBN		3	5
484	Protein NRT1/ PTR FAMILY 8.5 OS=Arabidopsis thaliana OX=3702 GN=NPF8.5 PE=2 SV=1	PTR17_ARATH	2		
485	60S ribosomal protein L15 OS=Quercus suber OX=58331 GN=RPL15 PE=2 SV=1	RL15_QUESU		2	
486	Isocitrate dehydrogenase [NAD] regulatory subunit 3, mitochondrial OS=Arabidopsis thaliar	IDH3_ARATH	2		3
487	Ubiquinol oxidase 1a, mitochondrial OS=Arabidopsis thaliana OX=3702 GN=AOX1A PE=1 SV	AOX1A_ARATH	2	2	2
488	Lectin (Fragment) OS=Allium cepa OX=4679 PE=1 SV=2	LEC_ALLCE	2	2	2
489	NADH-ubiquinone oxidoreductase chain 5 OS=Arabidopsis thaliana OX=3702 GN=ND5 PE=2	NU5M_ARATH (+1)	2		
490	Serine/threonine-protein phosphatase PP1 isozyme 5 OS=Arabidopsis thaliana OX=3702 GN	PP15_ARATH		2	2
491	Ras-related protein RABG1 OS=Arabidopsis thaliana OX=3702 GN=RABG1 PE=2 SV=1	RABG1_ARATH	2	2	2
492	Probable methyltransferase PMT21 OS=Arabidopsis thaliana OX=3702 GN=ERD3 PE=2 SV=1	PMTL_ARATH	3	3	
493	Vesicle-associated membrane protein 713 OS=Arabidopsis thaliana OX=3702 GN=VAMP713	VA713_ARATH	2		

494	Hypersensitive-induced response protein 4 OS=Arabidopsis thaliana OX=3702 GN=HIR4 PE=:	HIR4_ARATH			2
495	D-3-phosphoglycerate dehydrogenase 3, chloroplastic OS=Arabidopsis thaliana OX=3702 GI	SERA3_ARATH		2	2
496	50S ribosomal protein L3, chloroplastic OS=Spinacia oleracea OX=3562 GN=RPL3 PE=1 SV=2	RK3_SPIOL	2		
497	40S ribosomal protein S19 OS=Oryza sativa subsp. japonica OX=39947 GN=RPS19A PE=2 SV=	RS19_ORYSJ			2
498	Chaperone protein dnaJ A7A, chloroplastic OS=Oryza sativa subsp. japonica OX=39947 GN=I	DJA7A_ORYSJ (+2)	3	2	
499	Serine/threonine-protein kinase SAPK1 OS=Oryza sativa subsp. japonica OX=39947 GN=SAPI	SAPK1_ORYSJ	4	4	3
500	60S ribosomal protein L7a-1 OS=Oryza sativa subsp. japonica OX=39947 GN=RPL7A-1 PE=2 S	RL7A1_ORYSJ (+1)		2	
501	Probable inactive receptor kinase At3g08680 OS=Arabidopsis thaliana OX=3702 GN=At3g08	Y3868_ARATH	3	3	
502	Probable pyridoxal 5'-phosphate synthase subunit PDX1.1 OS=Oryza sativa subsp. japonica (	PDX11_ORYSJ	3	4	4
503	Vacuolar protein sorting-associated protein 20 homolog 2 OS=Arabidopsis thaliana OX=370	VP202_ARATH	2	2	
504	UDP-glucuronic acid decarboxylase 1 OS=Arabidopsis thaliana OX=3702 GN=UXS1 PE=1 SV=	UXS1_ARATH		2	
505	Peroxiredoxin-2C OS=Oryza sativa subsp. japonica OX=39947 GN=PRXIIC PE=1 SV=1	PRX2C_ORYSJ	2	2	2
506	Probable sucrose-phosphate synthase 3 OS=Arabidopsis thaliana OX=3702 GN=SPS3 PE=2 S\	SPSA3_ARATH	2		2
507	Protein TIC 40, chloroplastic OS=Arabidopsis thaliana OX=3702 GN=TIC40 PE=1 SV=1	TIC40_ARATH	2	2	2
508	Zeta-carotene desaturase, chloroplastic/chromoplastic OS=Narcissus pseudonarcissus OX=3	ZDS_NARPS	2	2	2
509	Magnesium transporter MRS2-11, chloroplastic OS=Arabidopsis thaliana OX=3702 GN=MRS	MRS2B_ARATH	2		
510	Ureidoglycolate hydrolase OS=Oryza sativa subsp. japonica OX=39947 GN=UAH PE=1 SV=2	UAH_ORYSJ		2	
511	Eukaryotic translation initiation factor 3 subunit A OS=Arabidopsis thaliana OX=3702 GN=TI	EIF3A_ARATH		3	
512	25.3 kDa vesicle transport protein OS=Arabidopsis thaliana OX=3702 GN=SEC22 PE=2 SV=1	SEC22_ARATH	2	2	2
513	Protein ILITYHIA OS=Arabidopsis thaliana OX=3702 GN=ILA PE=1 SV=1	ILA_ARATH	2		
514	NADH dehydrogenase [ubiquinone] iron-sulfur protein 5-A OS=Arabidopsis thaliana OX=370	NDS5A_ARATH (+1)	2	2	2
515	Ketol-acid reductoisomerase, chloroplastic OS=Spinacia oleracea OX=3562 GN=AHRI PE=1 S	ILV5_SPIOL	3	2	3
516	Probable phospholipid-transporting ATPase 5 OS=Arabidopsis thaliana OX=3702 GN=ALA5 F	ALA5_ARATH		2	
517	Pyridoxal 5'-phosphate synthase subunit PDX1.3 OS=Arabidopsis thaliana OX=3702 GN=PD)	PDX13_ARATH	4	2	4
518	Ribulose bisphosphate carboxylase small chain, chloroplastic OS=Flaveria trinervia OX=422	RBS_FLATR	2	2	
519	Protein TIC 214 OS=Calycanthus floridus var. glaucus OX=212734 GN=TIC214 PE=3 SV=1	TI214_CALFG	2		2
520	ATP-dependent zinc metalloprotease FTSH 11, chloroplastic/mitochondrial OS=Arabidopsis	FTSHB_ARATH	2		2
521	Thioredoxin reductase 2 OS=Arabidopsis thaliana OX=3702 GN=NTR2 PE=2 SV=2	TRXB2_ARATH		2	
522	Vacuolar-sorting receptor 1 OS=Arabidopsis thaliana OX=3702 GN=VSR1 PE=1 SV=2	VSR1_ARATH	3	2	2
523	Calcium-dependent protein kinase 18 OS=Oryza sativa subsp. japonica OX=39947 GN=CPK1:	CDPKI_ORYSJ	3	4	3
524	Receptor-like cytoplasmic kinase 1 OS=Arabidopsis thaliana OX=3702 GN=CARK1 PE=1 SV=1	CARK1_ARATH		2	2
525	Protein EXPORTIN 1A OS=Arabidopsis thaliana OX=3702 GN=XPO1 PE=1 SV=1	XPO1A_ARATH	2	2	
526	Nucleoside diphosphate kinase 2, chloroplastic OS=Nicotiana tabacum OX=4097 GN=NDPK:	NDK2_TOBAC			2
527	Photosystem I reaction center subunit psaK, chloroplastic OS=Arabidopsis thaliana OX=370	PSAK_ARATH	2	2	
528	Pyrophosphate--fructose 6-phosphate 1-phosphotransferase subunit beta OS=Solanum tub(	PFPB_SOLTU			2

529	ATP synthase subunit a, chloroplastic OS=Cicer arietinum OX=3827 GN=atpI PE=3 SV=1	ATPI_CICAR (+2)		2	
530	Delta-aminolevulinic acid dehydratase, chloroplastic OS=Oryza sativa subsp. japonica OX=3	HEM2_ORYSJ	2		2
531	Calreticulin OS=Oryza sativa subsp. japonica OX=39947 GN=Os07g0246200 PE=1 SV=2	CALR_ORYSJ	2		
532	ATP-citrate synthase beta chain protein 1 OS=Oryza sativa subsp. japonica OX=39947 GN=A	ACLB1_ORYSJ		4	
533	ABC transporter B family member 26, chloroplastic OS=Arabidopsis thaliana OX=3702 GN=	AB26B_ARATH	3		
534	Coatomer subunit gamma OS=Arabidopsis thaliana OX=3702 GN=At4g34450 PE=1 SV=2	COPG_ARATH	3	2	3
535	Proteasome subunit alpha type-1 OS=Oryza sativa subsp. japonica OX=39947 GN=PAF1 PE=2	PSA1_ORYSJ	2		
536	Clathrin light chain 2 OS=Oryza sativa subsp. japonica OX=39947 GN=Os06g0731800 PE=2 S	CLC2_ORYSJ	2		
537	Thioredoxin M-type, chloroplastic OS=Pisum sativum OX=3888 PE=2 SV=1	TRXM_PEA			2
538	Serine--glyoxylate aminotransferase OS=Arabidopsis thaliana OX=3702 GN=AGT1 PE=1 SV=2	SGAT_ARATH	2		2
539	Probable inactive ATP-dependent zinc metalloprotease FTSHI 2, chloroplastic OS=Arabidops	FTSI2_ARATH			2
540	Histidine--tRNA ligase, cytoplasmic OS=Oryza sativa subsp. japonica OX=39947 GN=Os05g01	SYHC_ORYSJ		2	
541	T-complex protein 1 subunit zeta 2 OS=Arabidopsis thaliana OX=3702 GN=CCT6B PE=1 SV=1	TCPZB_ARATH		2	2
542	(S)-2-hydroxy-acid oxidase GLO1 OS=Arabidopsis thaliana OX=3702 GN=GLO1 PE=1 SV=1	GLO1_ARATH	3	3	3
543	Phosphoenolpyruvate carboxykinase (ATP) OS=Cucumis sativus OX=3659 GN=PCK PE=2 SV=	PCKA_CUCSA	2		
544	Elongation factor 1-delta OS=Pimpinella brachycarpa OX=45043 PE=2 SV=3	EF1D_PIMBR	2	2	
545	Sedoheptulose-1,7-bisphosphatase, chloroplastic OS=Triticum aestivum OX=4565 PE=2 SV=	S17P_WHEAT		2	
546	DNA-directed RNA polymerase II subunit RPB2 OS=Solanum lycopersicum OX=4081 GN=RP	RPB2_SOLLC			2
547	Transmembrane 9 superfamily member 4 OS=Arabidopsis thaliana OX=3702 GN=TMN4 PE=2	TMN4_ARATH			2
548	Dynamin-related protein 1B OS=Arabidopsis thaliana OX=3702 GN=DRP1B PE=1 SV=1	DRP1B_ARATH	2		3
549	UDP-glucuronic acid decarboxylase 6 OS=Arabidopsis thaliana OX=3702 GN=UXS6 PE=2 SV=	UXS6_ARATH (+1)	2	2	
550	Probable receptor-like protein kinase At2g47060 OS=Arabidopsis thaliana OX=3702 GN=At2	Y2706_ARATH		2	2
551	Pheophorbide a oxygenase, chloroplastic OS=Arabidopsis thaliana OX=3702 GN=PAO PE=1 S	PAO_ARATH	2		
552	Long chain acyl-CoA synthetase 7, peroxisomal OS=Arabidopsis thaliana OX=3702 GN=LACS	LACS7_ARATH		2	
553	ABC transporter B family member 28 OS=Arabidopsis thaliana OX=3702 GN=ABCB28 PE=2 S	AB28B_ARATH	2		
554	DEAD-box ATP-dependent RNA helicase 12 OS=Arabidopsis thaliana OX=3702 GN=RH12 PE=	RH12_ARATH	2		2
555	Probable histone H2A.7 OS=Arabidopsis thaliana OX=3702 GN=At5g59870 PE=1 SV=1	H2A7_ARATH	2		2
556	ABC transporter B family member 18 OS=Arabidopsis thaliana OX=3702 GN=ABCB18 PE=3 S	AB18B_ARATH	2		
557	Guanine nucleotide-binding protein subunit beta-like protein OS=Medicago sativa OX=387	GBLP_MEDSA			3
558	Thiamine thiazole synthase 1, chloroplastic OS=Vitis vinifera OX=29760 GN=THI1-1 PE=3 SV=	THI41_VITVI		2	2
559	UDP-glucose 6-dehydrogenase 4 OS=Arabidopsis thaliana OX=3702 GN=UGD4 PE=1 SV=1	UGDH4_ARATH	2	3	
560	CDPK-related kinase 5 OS=Arabidopsis thaliana OX=3702 GN=CRK5 PE=2 SV=1	CAMK5_ARATH	2		
561	Probable methyltransferase PMT2 OS=Arabidopsis thaliana OX=3702 GN=At1g26850 PE=2 S	PMT2_ARATH	2	2	
562	L-ascorbate peroxidase T, chloroplastic OS=Arabidopsis thaliana OX=3702 GN=APXT PE=2 S	APXT_ARATH	2		
563	Exocyst complex component SEC8 OS=Arabidopsis thaliana OX=3702 GN=SEC8 PE=1 SV=1	SEC8_ARATH	2	2	

564	Eukaryotic translation initiation factor 3 subunit A OS=Zea mays OX=4577 GN=TIF3A1 PE=2 S	EIF3A_MAIZE			2
565	Phospholipid-transporting ATPase 1 OS=Arabidopsis thaliana OX=3702 GN=ALA1 PE=2 SV=1	ALA1_ARATH	2		
566	Histone H3-like 5 OS=Arabidopsis thaliana OX=3702 GN=At5g65350 PE=2 SV=3	H3L5_ARATH	3		
567	DNA damage-binding protein 1a OS=Arabidopsis thaliana OX=3702 GN=DDB1A PE=1 SV=1	DDB1A_ARATH			2
568	NAD(P)H-quinone oxidoreductase subunit H, chloroplastic OS=Dioscorea elephantipes OX=1	NDHH_DIOEL			2
569	Triosephosphate isomerase, chloroplastic OS=Fragaria ananassa OX=3747 GN=TPI PE=2 SV=1	TPIC_FRAAN		2	
570	Serine/threonine-protein kinase VPS15 OS=Arabidopsis thaliana OX=3702 GN=VPS15 PE=1 S	VPS15_ARATH		2	
571	Serine/threonine-protein kinase STY13 OS=Arabidopsis thaliana OX=3702 GN=STY13 PE=1 SV	STY13_ARATH	2	2	2
572	ABC transporter B family member 11 OS=Arabidopsis thaliana OX=3702 GN=ABCB11 PE=2 SV	AB11B_ARATH	2		
573	Histone H3.3b OS=Lilium longiflorum OX=4690 GN=soH3-1 PE=2 SV=3	H33B_LILLO		2	
574	Calcium-dependent protein kinase 13 OS=Oryza sativa subsp. japonica OX=39947 GN=CPK13	CDPKD_ORYSJ			2
575	Proteasome subunit alpha type-6 OS=Oryza sativa subsp. japonica OX=39947 GN=PAA1 PE=2	PSA6_ORYSJ		2	2
576	Mannosyl-oligosaccharide 1,2-alpha-mannosidase MNS3 OS=Arabidopsis thaliana OX=3702	MNS3_ARATH		2	
577	DEAD-box ATP-dependent RNA helicase 30 OS=Arabidopsis thaliana OX=3702 GN=RH30 PE=1	RH30_ARATH		2	
578	40S ribosomal protein S29 OS=Triticum aestivum OX=4565 GN=RPS29 PE=1 SV=1	RS29_WHEAT			2
579	Hypersensitive-induced response protein 2 OS=Arabidopsis thaliana OX=3702 GN=HIR2 PE=1	HIR2_ARATH			3
580	Dynein-1-alpha heavy chain, flagellar inner arm I1 complex OS=Chlamydomonas reinhardtii	DYH1A_CHLRE		2	
581	Importin subunit alpha-1a OS=Oryza sativa subsp. japonica OX=39947 GN=Os01g0253300 P	IMA1A_ORYSJ	2		
582	Phosphoglucomutase, cytoplasmic OS=Bromus inermis OX=15371 GN=PGM1 PE=2 SV=1	PGMC_BROIN	2	6	4
583	Probable aldo-keto reductase 1 OS=Glycine max OX=3847 GN=AKR1 PE=2 SV=1	AKR1_SOYBN		2	
584	Acetyl-coenzyme A synthetase, chloroplastic/glyoxysomal OS=Arabidopsis thaliana OX=3702	ACS_ARATH			2
585	Lysine--tRNA ligase OS=Solanum lycopersicum OX=4081 GN=LYSRS PE=2 SV=1	SYK_SOLLC		2	
586	ARF guanine-nucleotide exchange factor GNOM OS=Arabidopsis thaliana OX=3702 GN=GN P	GNOM_ARATH	2		
587	Preprotein translocase subunit SECY, chloroplastic OS=Zea mays OX=4577 GN=SECY PE=2 SV	SECY_MAIZE	2		2
588	Proteasome subunit alpha type-3 OS=Spinacia oleracea OX=3562 GN=PAG1 PE=2 SV=1	PSA3_SPIOL	3	3	3
589	50S ribosomal protein L22, chloroplastic OS=Platanus occidentalis OX=4403 GN=rpl22 PE=3	RK22_PLAOC	2	2	2
590	T-complex protein 1 subunit gamma OS=Arabidopsis thaliana OX=3702 GN=CCT3 PE=1 SV=1	TCPG_ARATH		2	
591	Alcohol dehydrogenase 2 OS=Solanum lycopersicum OX=4081 GN=ADH2 PE=2 SV=2	ADH2_SOLLC			2
592	Carotenoid 9,10(9',10')-cleavage dioxygenase 1 OS=Arabidopsis thaliana OX=3702 GN=CCD1	CCD1_ARATH		2	2
593	Ras-related protein RABC2a OS=Arabidopsis thaliana OX=3702 GN=RABC2A PE=1 SV=1	RAC2A_ARATH	3	3	3
594	Xylose isomerase OS=Arabidopsis thaliana OX=3702 GN=XYLA PE=2 SV=2	XYLA_ARATH		2	2
595	Probable methyltransferase PMT14 OS=Arabidopsis thaliana OX=3702 GN=At4g18030 PE=2	PMTE_ARATH	2	2	
596	60S ribosomal protein L6 OS=Mesembryanthemum crystallinum OX=3544 GN=RPL6 PE=2 SV	RL6_MESCR			2
597	Probable methyltransferase PMT9 OS=Arabidopsis thaliana OX=3702 GN=At5g14430 PE=2 S	PMT9_ARATH	2	2	
598	Probable protein disulfide-isomerase A6 OS=Medicago sativa OX=3879 PE=2 SV=1	PDIA6_MEDSA	2		

599	Ubiquitin-activating enzyme E1 3 OS=Triticum aestivum OX=4565 GN=UBA3 PE=1 SV=1	UBE13_WHEAT			3
600	Uncharacterized aarF domain-containing protein kinase At5g05200, chloroplastic OS=Arabi	Y5520_ARATH		2	
601	Pyrophosphate--fructose 6-phosphate 1-phosphotransferase subunit alpha OS=Ricinus com	PFPA_RICCO	2		
602	Photosystem II D2 protein OS=Nephroselmis olivacea OX=31312 GN=psbD PE=3 SV=1	PSBD_NEPOL		5	
603	Dolichyl-diphosphooligosaccharide--protein glycosyltransferase 48 kDa subunit OS=Arabido	OST48_ARATH	2		2
604	Probable leucine-rich repeat receptor-like protein kinase At1g68400 OS=Arabidopsis thaliar	Y1680_ARATH	2	2	
605	Vacuolar-sorting receptor 1 OS=Pisum sativum OX=3888 GN=BP80 PE=1 SV=1	VSR1_PEA		2	
606	Cytochrome b-c1 complex subunit Rieske, mitochondrial OS=Zea mays OX=4577 PE=2 SV=1	UCRI_MAIZE	2	2	
607	SPX domain-containing membrane protein At4g11810 OS=Arabidopsis thaliana OX=3702 G	SPXM2_ARATH	2		2
608	Probable protein phosphatase 2C 41 OS=Oryza sativa subsp. japonica OX=39947 GN=Os04g(	P2C41_ORYSJ	2		
609	DnaJ protein homolog 2 OS=Allium porrum OX=4681 GN=LDJ2 PE=2 SV=1	DNJH2_ALLPO	2		
610	Proteasome subunit alpha type-3 OS=Arabidopsis thaliana OX=3702 GN=PAG1 PE=1 SV=2	PSA3_ARATH	3		
611	Mannose-specific lectin OS=Allium sativum OX=4682 GN=LECASAL PE=1 SV=3	ASAL_ALLSA			2
612	Disease resistance protein LAZ5 OS=Arabidopsis thaliana OX=3702 GN=LAZ5 PE=1 SV=1	LAZ5_ARATH	2		

diplantae_20190430 database (7108828 entries)

Table S2 Lipid profiles of active and inactive GC-VLNs

			% total signal					% total signal	
			active1	active2	active3	active4	active5	average of active GC-ELNs	
Mass	Compound Fo	Compound Na	sample01	sample02	sample03	sample04	sample05	ave	stdev
926.6	C49H80O15	DGDG(34:6)	0	0	0.00169946	0.00136989	0	0.00061387	0.00084861
928.6	C49H82O15	DGDG(34:5)	0.00150404	0	0.00509838	0.00136989	0.00148997	0.001892456	0.00190035
930.6	C49H84O15	DGDG(34:4)	0.01102962	0.01532016	0.01487029	0.0242014	0.02036286	0.017156867	0.005149
932.6	C49H86O15	DGDG(34:3)	1.76975288	1.96528941	2.16086548	2.14479214	1.75170258	1.958480498	0.19627359
934.6	C49H88O15	DGDG(34:2)	0.13486219	0.18288442	0.20520999	0.17443274	0.1162173	0.162721329	0.03635846
936.6	C49H90O15	DGDG(34:1)	0.04762791	0.03638538	0.0565071	0.05662215	0.02930265	0.045289038	0.01219215
954.6	C51H84O15	DGDG(36:6)	2.6062494	2.55583374	2.74654244	2.66283913	2.30274143	2.574841227	0.16778781
956.6	C51H86O15	DGDG(36:5)	0.29328766	0.30831824	0.34796477	0.28722041	0.27266366	0.301890947	0.0287558
958.6	C51H88O15	DGDG(36:4)	0.1759726	0.17283057	0.1928889	0.17488937	0.14502329	0.172320945	0.01723654
960.6	C51H90O15	DGDG(36:3)	0.27824727	0.25852772	0.36156046	0.29680964	0.25031418	0.289091853	0.04434733
962.6	C51H92O15	DGDG(36:2)	0.02005386	0.01675643	0.03526383	0.02968096	0.01390634	0.023132284	0.00901812
964.7	C51H94O15	DGDG(36:1)	0	0.00909635	0.0012746	0.00593619	0.00297993	0.003857413	0.00367724
982.6	C53H88O15	DGDG(38:6)	0.00451212	0	0	0	0	0.000902424	0.00201788
984.6	C53H90O15	DGDG(38:5)	0	0	0.00679785	0	0	0.001359569	0.00304009
986.6	C53H92O15	DGDG(38:4)	0.00350942	0	0	0.00410967	0	0.001523819	0.00209734
988.7	C53H94O15	DGDG(38:3)	0.00501346	0.00861759	0.00169946	0	0.00198662	0.003463427	0.00340058
Total DGDG		Total DGDG	5.35162242	5.52986	6.13824301	5.8642736	4.9086908	5.558537967	0.47314639
764.5	C43H70O10	MGDG(34:6)	0.0140377	0.00622382	0.00339892	0.00228315	0.00595986	0.00638069	0.00459653
766.5	C43H72O10	MGDG(34:5)	0.01203231	0.0043088	0.00509838	0.00365304	0.00794648	0.006607804	0.00344644
768.5	C43H74O10	MGDG(34:4)	0.04512118	0.05697185	0.04588546	0.04246661	0.05910196	0.049909412	0.00756465
770.5	C43H76O10	MGDG(34:3)	0.43366466	0.35140619	0.38365347	0.3867658	0.4613926	0.403376543	0.04372947
772.6	C43H78O10	MGDG(34:2)	0.0355956	0.02633153	0.01784435	0.04292324	0.0387391	0.032286762	0.01012131
774.6	C43H80O10	MGDG(34:1)	0.00451212	0.00239378	0.00084973	0	0.01142307	0.003835738	0.0045753
792.5	C45H74O10	MGDG(36:6)	8.3529328	7.65242043	6.66401396	6.95310836	8.40290784	7.60507668	0.791948
794.5	C45H76O10	MGDG(36:5)	0.96333716	0.84236948	0.74755069	0.80937706	1.03428427	0.879383732	0.11693299
796.6	C45H78O10	MGDG(36:4)	0.44569697	0.34039483	0.29868038	0.3520619	0.39136423	0.365639662	0.05562402
798.6	C45H80O10	MGDG(36:3)	0.09525582	0.09766603	0.07265199	0.07123431	0.12267381	0.091896392	0.02114883
800.6	C45H82O10	MGDG(36:2)	0.00601616	0.00191502	0.00424865	0.01643869	0.00496655	0.006717014	0.00563955
802.6	C45H84O10	MGDG(36:1)	0.01654443	0.00766008	0.01274596	0.0091326	0.01887289	0.012991195	0.00475893
820.6	C47H78O10	MGDG(38:6)	0.03960637	0.02393775	0.02251787	0.03242075	0.04022906	0.031742359	0.00837153
822.6	C47H80O10	MGDG(38:5)	0.02707271	0.02154398	0.01402056	0.01461217	0.0198662	0.019423123	0.00537456
824.6	C47H82O10	MGDG(38:4)	0.00350942	0.00813884	0.00594812	0.00593619	0.00645652	0.005997817	0.00165776

826.6	C47H84O10	MGDG(38:3)	0	0.00574506	0.00042487	0	0	0.001233985	0.00252847
Total MGDG		Total MGDG	10.4949354	9.44942745	8.29953335	8.74241387	10.6261845	9.522498908	1.03359462
738.5	C38H73O10F	PG(32:1)	0.18108633	0.09785753	0.17249536	0.21224173	0.17382929	0.167502046	0.04213519
740.5	C38H75O10F	PG(32:0)	0.19933534	0.20107711	0.24625199	0.28256278	0.15575104	0.216995653	0.04865997
760.5	C40H71O10F	PG(34:4)	0.61063995	0.44773171	0.50440021	0.65078939	0.4700344	0.536719131	0.08928387
762.5	C40H73O10F	PG(34:3)	0.70469254	0.5040333	0.55793326	0.75691025	0.63273861	0.631261591	0.10337547
764.5	C40H75O10F	PG(34:2)	1.35323427	0.951765	1.39185908	1.76953343	1.14727331	1.32273302	0.30567251
766.5	C40H77O10F	PG(34:1)	0.16564486	0.11796524	0.15584063	0.21352029	0.14323533	0.159241271	0.03520896
768.5	C40H79O10F	PG(34:0)	0.02667163	0.00804308	0.02260284	0.03324268	0.03615649	0.025343345	0.0110442
784.5	C42H71O10F	PG(36:6)	0.00421131	0.00402154	0.00237925	0.00767139	0.0041719	0.004491078	0.00193486
786.5	C42H73O10F	PG(36:5)	0.01684524	0.00268103	0.01427548	0.0217356	0.01946888	0.015001245	0.00743347
788.5	C42H75O10F	PG(36:4)	0.03228671	0.01608617	0.03330945	0.03452124	0.01807825	0.026856364	0.00898518
790.5	C42H77O10F	PG(36:3)	0.00842262	0.00134051	0	0.00127856	0.01529698	0.005267735	0.006512
792.5	C42H79O10F	PG(36:2)	0.0280754	0.00268103	0.02260284	0.01278565	0.01668761	0.016566505	0.00969546
794.6	C42H81O10F	PG(36:1)	0.01263393	0.00268103	0.00832736	0.0178999	0.00139063	0.008586572	0.00688918
Total PG		Total PG	3.34378012	2.35796428	3.13227775	4.01469289	2.83411273	3.136565556	0.61476856
500.3	C22H43O9P	LPG(16:1)	0	0.0093836	0.00356887	0	0.00278127	0.003146747	0.00384103
502.3	C22H45O9P	LPG(16:0)	0	0	0	0.00894995	0	0.00178999	0.00400254
524.3	C24H43O9P	LPG(18:3)	0.02526786	0	0	0	0	0.005053572	0.01130013
526.3	C24H45O9P	LPG(18:2)	0	0	0	0	0	0	0
528.3	C24H47O9P	LPG(18:1)	0.00701885	0.00670257	0	0	0.00834381	0.004413045	0.00407532
Total LysoPG		Total LysoPG	0.03228671	0.01608617	0.00356887	0.00894995	0.01112507	0.014403355	0.01096037
494.3	C24H48O7PI	LPC(16:1)	0	0	0.00122463	0	0	0.000244926	0.00054767
496.3	C24H50O7PI	LPC(16:0)	0.00650284	0.01310961	0.03122802	0.01250377	0.00787355	0.014243558	0.00991565
518.3	C26H48O7PI	LPC(18:3)	0.0057803	0.01793947	0.02449256	0.01052949	0.00143155	0.012034676	0.00927193
520.3	C26H50O7PI	LPC(18:2)	0.03179168	0.03449897	0.07225305	0.03553704	0.02863108	0.040542365	0.01792761
522.3	C26H52O7PI	LPC(18:1)	0	0.00068998	0.00673545	0.00460665	0	0.002406417	0.00308664
524.4	C26H54O7PI	LPC(18:0)	0.00722538	0.00275992	0	0	0.00143155	0.002283371	0.00299181
Total LysoPC		Total LysoPC	0.05130021	0.06899795	0.13593371	0.06317696	0.03936774	0.071755312	0.03764115
452.3	C21H42O7PI	LPE(16:1)	0	0	0	0	0	0	0
454.3	C21H44O7PI	LPE(16:0)	0.08185006	0.10333252	0.14225444	0.14404513	0.07696123	0.109688676	0.0321223
476.3	C23H42O7PI	LPE(18:3)	0.00832374	0.01059821	0.01528353	0.03032529	0	0.012906153	0.01120237
478.3	C23H44O7PI	LPE(18:2)	0.10959585	0.0331194	0.20691554	0.14530868	0.09895016	0.118777925	0.06381018
480.3	C23H46O7PI	LPE(18:1)	0	0.00927343	0	0	0	0.001854686	0.0041472
Total LysoPE		Total LysoPE	0.19976964	0.15632356	0.36445352	0.3196791	0.17591139	0.243227441	0.09288828
734.6	C40H80O8PI	PC(32:0)	0.080755	0.08642318	0.08141492	0.08496557	0.09517163	0.085746061	0.00577881
754.5	C42H76O8PI	PC(34:4)	0.10442457	0.09971905	0.09203425	0.08877	0.09793023	0.096575622	0.00622242
756.5	C42H78O8PI	PC(34:3)	5.44260877	5.37951042	4.7834214	5.25518392	4.89099439	5.150343779	0.29615104

758.6	C42H80O8PI	PC(34:2)	17.6909151	17.8523694	16.0292994	16.8637634	16.3474522	16.95675989	0.8033037
760.6	C42H82O8PI	PC(34:1)	0.80267689	0.92605759	0.84187745	0.98090848	0.90688912	0.891681905	0.07031484
778.5	C44H76O8PI	PC(36:6)	1.03589177	0.99719051	0.89910387	0.9739337	0.88964788	0.959153547	0.0632278
780.5	C44H78O8PI	PC(36:5)	4.23058755	4.16493238	3.6701609	3.85959272	3.88065784	3.961186278	0.23215909
782.6	C44H80O8PI	PC(36:4)	10.0769713	9.73856256	8.82407917	9.65627363	9.57854217	9.574885771	0.46076781
784.6	C44H82O8PI	PC(36:3)	1.47029799	1.44792063	1.29791898	1.39242083	1.52826333	1.427364353	0.08721183
786.6	C44H84O8PI	PC(36:2)	1.63598498	1.681928	1.52446484	1.6460494	1.47309137	1.592303719	0.0889422
788.6	C44H86O8PI	PC(36:1)	0.41282515	0.32907287	0.37698647	0.33542378	0.34413511	0.359688675	0.03496686
806.6	C46H80O8PI	PC(38:6)	0.05569311	0.04919473	0.05427661	0.05960271	0.05379266	0.054511965	0.00374688
808.6	C46H82O8PI	PC(38:5)	0.19492587	0.19411975	0.21710645	0.18261257	0.18344677	0.194442282	0.01391802
810.6	C46H84O8PI	PC(38:4)	0.32998165	0.38026198	0.28436225	0.31830385	0.30482509	0.323546965	0.03595636
812.6	C46H86O8PI	PC(38:3)	0.40516734	0.30314592	0.32447974	0.37410214	0.34206616	0.349792261	0.04041555
814.6	C46H88O8PI	PC(38:2)	0.33972795	0.3257489	0.34099871	0.31069499	0.32275597	0.327985306	0.01263468
836.6	C48H86O8PI	PC(40:5)	0.04733914	0.04520597	0.03539779	0.053262	0.02482738	0.041206456	0.01119103
838.6	C48H88O8PI	PC(40:4)	0.02923888	0.01595505	0.01533904	0.01902214	0.01931019	0.01977306	0.00558161
840.6	C48H90O8PI	PC(40:3)	0.05847776	0.06514978	0.05191676	0.06974786	0.05517196	0.060092824	0.00728807
842.7	C48H92O8PI	PC(40:2)	0.14897906	0.16088007	0.16400976	0.14964085	0.1379299	0.15228793	0.01043234
Total PC		Total PC	44.5934698	44.2433487	39.9086488	42.6742746	41.4769014	42.57932865	1.94824403
686.5	C37H68O8PI	PE(32:3)	0.01095097	0.012549	0.01299254	0.01396392	0.01518789	0.013128865	0.0015839
688.5	C37H70O8PI	PE(32:2)	0.06789601	0.10039203	0.10579639	0.06981959	0.10848496	0.090477796	0.01996189
690.5	C37H72O8PI	PE(32:1)	0.05475485	0.06692802	0.06310662	0.0658299	0.06509098	0.063142072	0.00489139
692.5	C37H74O8PI	PE(32:0)	0.02847252	0.02091501	0.02969723	0.0219433	0.03688489	0.027582589	0.0064818
712.5	C39H70O8PI	PE(34:4)	0.05694504	0.06901952	0.05753839	0.06383506	0.07376977	0.064221555	0.00727926
714.5	C39H72O8PI	PE(34:3)	3.12869187	3.53149864	3.54232305	3.32640495	3.58976739	3.42373718	0.19349836
716.5	C39H74O8PI	PE(34:2)	17.8927884	20.3722609	20.3008427	19.9434684	21.1599918	19.93387044	1.2243709
718.5	C39H76O8PI	PE(34:1)	0.08541756	0.0104575	0.05753839	0	0.06726068	0.044134825	0.03708359
736.5	C41H70O8PI	PE(36:6)	0.21901938	0.22797356	0.23200963	0.20945878	0.20612143	0.218916556	0.01125426
738.5	C41H72O8PI	PE(36:5)	1.2046066	1.34901784	1.2936857	1.30263414	1.44718939	1.319426736	0.08849744
740.5	C41H74O8PI	PE(36:4)	3.67624033	4.23842581	4.2179351	4.07447203	4.58999874	4.159414401	0.33005406
742.5	C41H76O8PI	PE(36:3)	0.4248976	0.61699265	0.54011842	0.5605516	0.60317639	0.549147333	0.07611127
744.5	C41H78O8PI	PE(36:2)	0.84103443	0.90143673	0.90390952	0.84182482	0.94815857	0.887272813	0.04579296
746.6	C41H80O8PI	PE(36:1)	0.15659886	0.14535929	0.16797497	0.17255414	0.17466079	0.163429609	0.0122806
764.5	C43H74O8PI	PE(38:6)	0.00657058	0.0146405	0.03155331	0.01196907	0.02386669	0.017720032	0.00994998
766.5	C43H76O8PI	PE(38:5)	0.0350431	0.05647051	0.05011408	0.05585568	0.05858188	0.05121305	0.00956778
768.5	C43H78O8PI	PE(38:4)	0.18507138	0.1809148	0.17725536	0.14861599	0.18984868	0.176341242	0.01619497
770.6	C43H80O8PI	PE(38:3)	0.10293911	0.11503253	0.12806932	0.12368042	0.10631526	0.115207328	0.01080316
798.6	C45H84O8PI	PE(40:3)	0.05037446	0.06274502	0.06867485	0.06184021	0.07810917	0.064348742	0.01015148
800.6	C45H86O8PI	PE(40:2)	0.55521413	0.6075809	0.73036632	0.61939954	0.75830988	0.654174156	0.08634638

824.6	C47H86O8PI	PE(42:4)	0	0.0062745	0.00185608	0.00598454	0	0.002823023	0.00311375
826.6	C47H88O8PI	PE(42:3)	0.04380388	0.07111102	0.06867485	0.05585568	0.05641218	0.05917152	0.01104127
828.6	C47H90O8PI	PE(42:2)	0.73371493	0.85333221	0.81481783	0.762031	0.8592009	0.804619373	0.0554466
Total PE		Total PE	29.561046	33.6313284	33.5968507	32.5119927	35.2163883	32.90352124	2.10268866
822.5	C41H73O13F	PI(32:3)	0.00268065	0.00426642	0.00151447	0.00651081	0.00531113	0.004056698	0.00199979
824.5	C41H75O13F	PI(32:2)	0	0.00170657	0.00151447	0.00325541	0.00088519	0.001472328	0.0011988
826.5	C41H77O13F	PI(32:1)	0	0	0.00454342	0	0.00177038	0.00126276	0.00198772
828.5	C41H79O13F	PI(32:0)	0.00134032	0.00127993	0.02461021	0.00366233	0.00398335	0.006975228	0.00993866
848.5	C43H75O13F	PI(34:4)	0.00089355	0	0.00530066	0	0	0.001238842	0.00230336
850.5	C43H77O13F	PI(34:3)	0.0352952	0.01066606	0.65614612	0.08830291	0.02611305	0.163304666	0.27704894
852.5	C43H79O13F	PI(34:2)	0.10097108	0.04266424	1.95140051	0.28077883	0.09648549	0.49446003	0.81940099
854.5	C43H81O13F	PI(34:1)	0.00089355	0	0.01287303	0.00895237	0.00265556	0.005074903	0.00558876
872.5	C45H75O13F	PI(36:6)	0.00268065	0.00170657	0.00757237	0.00081385	0.00088519	0.002731726	0.00280951
874.5	C45H77O13F	PI(36:5)	0	0.00085328	0.01968817	0.00244156	0	0.004596602	0.00849514
876.5	C45H79O13F	PI(36:4)	0.00446775	0.00085328	0.03559015	0.00732467	0.00354075	0.01035532	0.01429486
878.5	C45H81O13F	PI(36:3)	0	0.00085328	0.01287303	0.00244156	0.00088519	0.003410612	0.00536252
880.6	C45H83O13F	PI(36:2)	0.00446775	0	0.03104673	0.00488311	0.00354075	0.008787668	0.01259093
882.6	C45H85O13F	PI(36:1)	0.0017871	0	0.00984408	0.0016277	0.00088519	0.002828815	0.00398503
Total PI		Total PI	0.15547759	0.06484964	2.77451745	0.4109951	0.14694121	0.710556198	1.16107536
756.5	C40H70O10F	PS(34:4)	0	0	0	0.00357328	0	0.000714657	0.00159802
758.5	C40H72O10F	PS(34:3)	0.04413603	0.02903474	0.05070188	0.05806586	0.03012031	0.042411762	0.01271588
760.5	C40H74O10F	PS(34:2)	0.31483701	0.1938771	0.23688582	0.22958346	0.17586374	0.230209428	0.05357305
762.5	C40H76O10F	PS(34:1)	0	0	0.00498707	0.00893321	0.00582974	0.003950003	0.00389379
780.5	C42H70O10F	PS(36:6)	0.0019616	0	0.00166236	0.00178664	0	0.00108212	0.00099354
782.5	C42H72O10F	PS(36:5)	0.02157761	0.00936604	0.01329885	0.01071985	0.00971623	0.012935718	0.00507031
784.5	C42H74O10F	PS(36:4)	0.04904003	0.03371776	0.03324713	0.03751948	0.04080816	0.038866512	0.00646475
786.5	C42H76O10F	PS(36:3)	0.01765441	0.00749283	0.00831178	0.00893321	0.00388649	0.009255746	0.005086
788.5	C42H78O10F	PS(36:2)	0.03530882	0.0206053	0.03324713	0.03573283	0.0213757	0.029253958	0.00760672
790.6	C42H80O10F	PS(36:1)	0.0019616	0.00187321	0	0.00178664	0.00388649	0.001901589	0.001376
808.5	C44H74O10F	PS(38:6)	0.0019616	0	0	0	0.00194325	0.000780969	0.00106941
810.5	C44H76O10F	PS(38:5)	0	0	0	0	0	0	0
812.5	C44H78O10F	PS(38:4)	0.00784641	0	0.00332471	0	0	0.002234224	0.00345185
814.6	C44H80O10F	PS(38:3)	0.0058848	0	0.00332471	0.00893321	0.00194325	0.004017194	0.00348475
816.6	C44H82O10F	PS(38:2)	0.06375204	0.02341511	0.04239009	0.04019944	0.0340068	0.040752697	0.01481601
818.6	C44H84O10F	PS(38:1)	0	0	0	0	0.00194325	0.000388649	0.00086905
840.6	C46H82O10F	PS(40:4)	0	0	0.00166236	0	0	0.000332471	0.00074343
842.6	C46H84O10F	PS(40:3)	0.08434886	0.05432305	0.0881049	0.10005194	0.05635413	0.076636575	0.02030118
844.6	C46H86O10F	PS(40:2)	0.85035417	0.4299014	0.71398217	0.68875038	0.46735061	0.630067748	0.17716858

846.6	C46H88O10F	PS(40:1)	0.02452002	0.01966869	0.00249353	0.01339981	0	0.012016411	0.01062886
868.6	C48H86O10F	PS(42:4)	0	0	0	0.00178664	0	0.000357328	0.00079901
870.6	C48H88O10F	PS(42:3)	0.08042565	0.02435171	0.06483191	0.05895918	0.04275141	0.054263972	0.02147931
872.6	C48H90O10F	PS(42:2)	0.81210295	0.43645763	0.64665672	0.65569751	0.41974109	0.594131181	0.16532838
874.6	C48H92O10F	PS(42:1)	0	0	0	0	0.00194325	0.000388649	0.00086905
898.6	C50H92O10F	PS(44:3)	0	0	0	0	0	0	0
900.7	C50H94O10F	PS(44:2)	0	0.00187321	0	0	0	0.000374642	0.00083772
Total PS		Total PS	2.41767363	1.2859578	1.94911314	1.96441258	1.31946388	1.787324203	0.48092045
666.5	C35H69O8P	PA(32:0)	0.02285277	0.00484955	0.00860736	0.00231272	0.00754631	0.009233742	0.00799749
682.4	C37H61O8P	PA(34:6)	0	0	0	0	0	0	0
684.4	C37H63O8P	PA(34:5)	0.00507839	0	0.00215184	0	0	0.001446047	0.00223412
686.4	C37H65O8P	PA(34:4)	0.01777438	0.02182299	0.02797391	0.01387632	0.01257718	0.018804957	0.0062713
688.5	C37H67O8P	PA(34:3)	0.49895218	0.38675192	0.45081031	0.4521368	0.34084164	0.42589857	0.06207457
690.5	C37H69O8P	PA(34:2)	1.89297123	1.6403616	1.93773121	1.7356965	1.77464043	1.796280194	0.12021524
692.5	C37H71O8P	PA(34:1)	0.06094072	0	0.01721471	0.0462544	0.00251544	0.025385056	0.02708279
710.4	C39H65O8P	PA(36:6)	0.09014149	0.08850436	0.07854213	0.06359981	0.05659732	0.075477021	0.01493037
712.5	C39H67O8P	PA(36:5)	0.27423326	0.21822993	0.26682806	0.21508298	0.23142015	0.241158875	0.02762858
714.5	C39H69O8P	PA(36:4)	0.71224471	0.65832694	0.67244975	0.68340882	0.6276014	0.670806323	0.03122925
716.5	C39H71O8P	PA(36:3)	0.11807265	0.10062824	0.13448995	0.10522877	0.12954498	0.117592919	0.01474164
718.5	C39H73O8P	PA(36:2)	0.10537667	0.07638047	0.10006052	0.10754149	0.06162819	0.090197469	0.02022137
Total PA		Total PA	3.79863844	3.19585601	3.69685976	3.4251386	3.24491304	3.472281173	0.26798982
Total		Total	100	100	100	100	100	100	7.1054E-15

		% total signal					% total signal		
		inactive1	inactive2	inactive3	inactive4	inactive5	averaage of inactive GC-EL		active/inactive
Compound Fo	Compound Na	sample06	sample07	sample08	sample09	sample10	ave	stdev	
C49H80O15	DGDG(34:6)	0.00548318	0.00277711	0.00403365	0.01099896	0.00155766	0.00497011	0.00367224	0.12351241
C49H82O15	DGDG(34:5)	0	0.00971987	0.00230495	0.00289446	0.00207687	0.00339923	0.00369818	0.55673072
C49H84O15	DGDG(34:4)	0.0274159	0.01527408	0.02938805	0.02605018	0.01557655	0.02274095	0.00678367	0.75444806
C49H86O15	DGDG(34:3)	4.43150532	3.56858063	3.40670908	3.51503707	3.70670048	3.72570652	0.40906684	0.52566687
C49H88O15	DGDG(34:2)	0.15791556	0.22077989	0.21666485	0.21766369	0.22014862	0.20663452	0.02728786	0.78748375
C49H90O15	DGDG(34:1)	0.08553759	0.05693066	0.04379396	0.05383703	0.06645996	0.06131184	0.01577446	0.73866707
C51H84O15	DGDG(36:6)	6.1669315	5.69202496	4.74847533	5.06097029	4.99410262	5.33250094	0.58199229	0.48285809
C51H86O15	DGDG(36:5)	0.19191127	0.1763462	0.25757763	0.20550694	0.26687828	0.21964407	0.04035374	1.37445529
C51H88O15	DGDG(36:4)	0.29280176	0.22008561	0.1809382	0.21997926	0.15940006	0.21464098	0.05087899	0.80283338
C51H90O15	DGDG(36:3)	0.2785455	0.27215634	0.22415593	0.29812979	0.22482159	0.25956183	0.03341763	1.11376875
C51H92O15	DGDG(36:2)	0.01425627	0.05901349	0.02881182	0	0.02388405	0.02519312	0.02186855	0.91819829
C51H94O15	DGDG(36:1)	0.00328991	0	0	0.00405225	0.01038437	0.00354531	0.00424951	1.08803414
C53H88O15	DGDG(38:6)	0	0	0	0.02084014	0	0.00416803	0.00931999	0.21651091
C53H90O15	DGDG(38:5)	0.02193272	0	0	0.01215675	0	0.00681789	0.00995505	0.19941195
C53H92O15	DGDG(38:4)	0.00328991	0.00347138	0.00460989	0.00521004	0	0.00331624	0.00201756	0.45950173
C53H94O15	DGDG(38:3)	0	0.00694276	0.00864354	0.00231557	0	0.00358038	0.0040056	0.96733623
	Total DGDG	11.6808164	10.304103	9.15610688	9.65564243	9.69199112	10.097732	0.97409853	0.55047391
C43H70O10	MGDG(34:6)	0.0153529	0.00208283	0.01555838	0.01215675	0.01038437	0.01110705	0.00549778	0.57447231
C43H72O10	MGDG(34:5)	0.0274159	0.00833132	0.01440591	0.01042007	0.0083075	0.01377614	0.00801958	0.47965577
C43H74O10	MGDG(34:4)	0.05373515	0.0263825	0.04091278	0.03878582	0.03530685	0.03902462	0.00992052	1.2789211
C43H76O10	MGDG(34:3)	0.85866584	0.67067099	0.65979058	0.54473812	0.50779564	0.64833223	0.13725234	0.62217567
C43H78O10	MGDG(34:2)	0.03399571	0.02429967	0.03054052	0.0405225	0.03011467	0.03189461	0.00594853	1.01229509
C43H80O10	MGDG(34:1)	0.00438654	0.00485993	0.00403365	0	0.01038437	0.0047329	0.0037069	0.8104414
C45H74O10	MGDG(36:6)	19.6144282	16.9771405	15.6419347	13.6659223	13.0095374	15.7817926	2.65987868	0.48188928
C45H76O10	MGDG(36:5)	0.58615184	0.43149277	0.41460203	0.31694381	0.28686819	0.40721173	0.11757403	2.15952457
C45H78O10	MGDG(36:4)	0.3706629	0.32839273	0.28350827	0.26108065	0.21547565	0.29182404	0.05999212	1.25294565
C45H80O10	MGDG(36:3)	0.05483179	0.06248487	0.08355427	0.05152146	0.03426842	0.05733216	0.01793113	1.60287681
C45H82O10	MGDG(36:2)	0	0.00138855	0	0.00926228	0.00674984	0.00348014	0.00426385	1.93010132
C45H84O10	MGDG(36:1)	0.01425627	0.0145798	0.02362569	0	0.00882671	0.01225769	0.00866949	1.05983999
C47H78O10	MGDG(38:6)	0.06470151	0.05137645	0.06741965	0.0480481	0.045172	0.05534354	0.01007255	0.57355125
C47H80O10	MGDG(38:5)	0.00328991	0.02152257	0.03515042	0.01157786	0.01557655	0.01742346	0.01192043	1.11476841
C47H82O10	MGDG(38:4)	0	0.01110842	0.00749107	0.00115779	0.0057114	0.00509374	0.00457556	1.17748868

C47H84O10	MGDG(38:3)	0	0.00069428	0.00172871	0	0.00311531	0.00110766	0.00132675	1.11404768
	Total MGDG	21.7018744	18.6368082	17.3242566	15.0121375	14.2335949	17.3817343	2.99031336	0.54784515
C38H73O10F	PG(32:1)	0.10747031	0.21383713	0.17586732	0.30310827	0.23987892	0.20803239	0.07286056	0.80517291
C38H75O10F	PG(32:0)	0.24564642	0.21772508	0.24201925	0.35335616	0.30675426	0.27310023	0.05558722	0.79456414
C40H71O10F	PG(34:4)	0.6847394	0.92727554	0.86804238	1.12004179	1.2110251	0.96222484	0.20850523	0.55778973
C40H73O10F	PG(34:3)	0.76764507	0.90589183	0.91483277	1.29834077	1.19067174	1.01547644	0.22037692	0.62164081
C40H75O10F	PG(34:2)	1.78093656	2.19669049	1.95712901	2.89168531	2.83638652	2.33256558	0.5074839	0.56707217
C40H77O10F	PG(34:1)	0.02149406	0	0.020975	0.06969869	0.03925291	0.03028413	0.02605283	5.25824084
C40H79O10F	PG(34:0)	0	0.01555179	0.04356347	0.048627	0.01599193	0.02474684	0.02060298	1.02410447
C42H71O10F	PG(36:6)	0	0.00388795	0	0	0	0.00077759	0.00173874	5.77564065
C42H73O10F	PG(36:5)	0.01228232	0.02138371	0.01129423	0.016209	0.0130843	0.01485071	0.00408933	1.01013629
C42H75O10F	PG(36:4)	0.0307058	0.05637524	0.04517693	0.05348969	0.02035336	0.04122021	0.01534478	0.65153395
C42H77O10F	PG(36:3)	0.00307058	0	0.01129423	0.0097254	0.00145381	0.0051088	0.00507902	1.03110917
C42H79O10F	PG(36:2)	0	0.00388795	0.01774808	0.0129672	0.01453812	0.00982827	0.0075273	1.68559757
C42H81O10F	PG(36:1)	0.00921174	0	0.00322692	0.0048627	0.00581525	0.00462332	0.00338588	1.85722987
	Total PG	3.66320227	4.56250671	4.31116959	6.18211197	5.89520623	4.92283936	1.07493372	0.63714562
C22H43O9P	LPG(16:1)	0	0	0	0.0194508	0.00290762	0.00447168	0.0084677	0.70370517
C22H45O9P	LPG(16:0)	0	0	0.00322692	0	0	0.00064538	0.00144312	2.77352492
C24H43O9P	LPG(18:3)	0	0	0	0	0	0	0	#DIV/0!
C24H45O9P	LPG(18:2)	0	0	0	0	0	0	0	#DIV/0!
C24H47O9P	LPG(18:1)	0.03684696	0	0	0	0	0.00736939	0.01647846	0.59883432
	Total LysoPG	0.03684696	0	0.00322692	0.0194508	0.00290762	0.01248646	0.01561522	1.15351775
C24H48O7PN	LPC(16:1)	0	0	0	0	0	0	0	#DIV/0!
C24H50O7PN	LPC(16:0)	0.0142242	0.00300176	0.00249141	0.01585165	0.01122443	0.00935869	0.00626264	1.52196065
C26H48O7PN	LPC(18:3)	0.00632187	0.0080047	0.00332188	0.00834298	0.00748295	0.00669487	0.00203526	1.79759548
C26H50O7PN	LPC(18:2)	0.01580466	0.00400235	0.0132875	0.02669752	0.06734657	0.02542772	0.02478829	1.59441596
C26H52O7PN	LPC(18:1)	0	0	0	0	0	0	0	#DIV/0!
C26H54O7PN	LPC(18:0)	0.01580466	0.01000588	0	0.00500579	0	0.00616327	0.00680135	0.37048065
	Total LysoPC	0.05215539	0.0250147	0.01910079	0.05589794	0.08605395	0.04764455	0.02688217	1.50605495
C21H42O7PN	LPE(16:1)	0	0	0	0	0	0	0	#DIV/0!
C21H44O7PN	LPE(16:0)	0.05462155	0.06724028	0.07175335	0.08169536	0.13074364	0.08121084	0.02934853	1.3506655
C23H42O7PN	LPE(18:3)	0.00910359	0	0	0.00800935	0.01293069	0.00600873	0.00578152	2.14790178
C23H44O7PN	LPE(18:2)	0.10317404	0.06147683	0.11002181	0.10251967	0.21263801	0.11796607	0.05629014	1.0068821
C23H46O7PN	LPE(18:1)	0	0	0.00318904	0	0	0.00063781	0.00142618	2.90790875
	Total LysoPE	0.16689917	0.12871711	0.1849642	0.19222438	0.35631234	0.20582344	0.08764622	1.18172857
C40H80O8PN	PC(32:0)	0.03350107	0.03277818	0.03680713	0.03376144	0.01730354	0.03083027	0.0077171	2.78122952
C42H76O8PN	PC(34:4)	0.05786548	0.04049069	0.04640899	0.05466138	0.05335257	0.05055582	0.00701091	1.91027692
C42H78O8PN	PC(34:3)	2.75317856	2.73794212	2.72212747	2.93885299	2.75558824	2.78153788	0.08896048	1.85161735

C42H80O8PN	PC(34:2)	9.73662815	9.87780104	10.238784	10.1316474	9.82985088	9.9629423	0.21256206	1.70198315
C42H82O8PN	PC(34:1)	0.02284164	0	0	0	0.01658256	0.00788484	0.01102121	113.088164
C44H76O8PN	PC(36:6)	0.52992596	0.53601965	0.53450357	0.55304454	0.52199003	0.53509675	0.01142202	1.79248621
C44H78O8PN	PC(36:5)	2.6937903	2.74469057	2.88295864	2.74030356	2.68276919	2.74890245	0.07980241	1.44100649
C44H80O8PN	PC(36:4)	7.44789619	7.9197868	7.68868986	7.59391252	7.21629584	7.57331624	0.26314011	1.26429235
C44H82O8PN	PC(36:3)	0.2954185	0.29114737	0.35366853	0.3633374	0.36049035	0.33281243	0.0362874	4.28879521
C44H84O8PN	PC(36:2)	0.61215585	0.55530094	0.56170884	0.63182124	0.54650337	0.58149805	0.03799623	2.7382787
C44H86O8PN	PC(36:1)	0.14770925	0.16099871	0.13842682	0.09083435	0.15789477	0.13917278	0.02844695	2.58447573
C46H80O8PN	PC(38:6)	0.03959217	0.01928128	0.0320062	0.02250763	0.04902669	0.03248279	0.01222962	1.6781797
C46H82O8PN	PC(38:5)	0.17359644	0.13496898	0.14082729	0.14790726	0.13987026	0.14743404	0.01533731	1.31884249
C46H84O8PN	PC(38:4)	0.25582633	0.20630972	0.22084279	0.23633008	0.20331656	0.2245251	0.02187628	1.44102806
C46H86O8PN	PC(38:3)	0.27105408	0.19859721	0.19683814	0.19131483	0.20331656	0.21222416	0.03316598	1.6482207
C46H88O8PN	PC(38:2)	0.22232526	0.16967529	0.16323163	0.16398414	0.18457106	0.18075747	0.02476762	1.8145048
C48H86O8PN	PC(40:5)	0.03045552	0.02313754	0.01440279	0.01125381	0.01297765	0.01844546	0.00812738	2.23396172
C48H88O8PN	PC(40:4)	0.01827331	0.01156877	0	0.00643075	0.01009373	0.00927331	0.00672712	2.13225443
C48H90O8PN	PC(40:3)	0.02740996	0.03663444	0.04160806	0.02250763	0.02451334	0.03053469	0.00822011	1.96801835
C48H92O8PN	PC(40:2)	0.11268541	0.09062203	0.0800155	0.08359976	0.07209807	0.08780415	0.01542756	1.73440463
	Total PC	25.4821294	25.7877513	26.0938563	26.0180128	25.0584053	25.688031	0.42495597	1.65755517
C37H68O8PN	PE(32:3)	0	0.01819819	0.00755208	0.01011586	0.01360962	0.00989515	0.00682258	1.32679797
C37H70O8PN	PE(32:2)	0.07665263	0.08795793	0.11076382	0.06828205	0.08619426	0.08597014	0.01595629	1.05243285
C37H72O8PN	PE(32:1)	0.00958158	0.02123123	0.02265624	0.02528965	0.03175578	0.02210289	0.00808058	2.85673336
C37H74O8PN	PE(32:0)	0.00958158	0.01213213	0.00503472	0.01011586	0.01814616	0.01100209	0.00476255	2.50703202
C39H70O8PN	PE(34:4)	0.04790789	0.06369368	0.05789927	0.05816619	0.05897502	0.05732841	0.00576632	1.12023963
C39H72O8PN	PE(34:3)	2.5894215	2.43400834	2.46323631	2.34055686	2.44632914	2.45471043	0.08909212	1.39476214
C39H74O8PN	PE(34:2)	15.6395309	15.6125333	15.6818913	15.1421763	15.6090996	15.5370463	0.22265187	1.28298971
C39H76O8PN	PE(34:1)	0	0	0	0	0	0	0	#DIV/0!
C41H70O8PN	PE(36:6)	0.14851446	0.16378374	0.16362837	0.13150617	0.14516928	0.1505204	0.01361924	1.45439789
C41H72O8PN	PE(36:5)	1.17853411	1.14042011	1.25867977	1.10009966	1.17950037	1.1714468	0.05868933	1.12632236
C41H74O8PN	PE(36:4)	4.33326872	4.34481863	4.5174017	4.19681697	4.44013842	4.36648889	0.12099656	0.95257643
C41H76O8PN	PE(36:3)	0.28744734	0.26993987	0.21901028	0.214962	0.18372987	0.23501787	0.042595	2.33661946
C41H78O8PN	PE(36:2)	0.32577366	0.31240232	0.32725674	0.31864956	0.32209433	0.32123532	0.00596448	2.76206493
C41H80O8PN	PE(36:1)	0.15570065	0.11070568	0.12712666	0.15553133	0.15310822	0.14043451	0.02050911	1.16374254
C43H74O8PN	PE(38:6)	0.02874473	0.02426426	0.02013888	0.02023172	0.0226827	0.02321246	0.003547	0.76338459
C43H76O8PN	PE(38:5)	0.04790789	0.04246245	0.06796871	0.05816619	0.05897502	0.05509605	0.01001817	0.92952306
C43H78O8PN	PE(38:4)	0.19881775	0.18956451	0.15230025	0.16564719	0.19393708	0.18005336	0.02008167	0.97938325
C43H80O8PN	PE(38:3)	0.06228026	0.05156155	0.07048607	0.09610066	0.07031637	0.07014898	0.01643959	1.64232364
C45H84O8PN	PE(40:3)	0.01437237	0.04549548	0.05538191	0.07081101	0.07031637	0.05127543	0.02321801	1.25496254
C45H86O8PN	PE(40:2)	0.67789665	0.58689173	0.640668	0.57028155	0.57046989	0.60924157	0.04801506	1.07375168

C47H86O8PN	PE(42:4)	0	0.01819819	0.00251736	0.00758689	0.00453654	0.0065678	0.00707058	0.42982798
C47H88O8PN	PE(42:3)	0.02874473	0.04549548	0.05286455	0.05816619	0.05217021	0.04748823	0.01140323	1.24602488
C47H90O8PN	PE(42:2)	0.74257231	0.79465444	0.71744747	0.67017565	0.69182233	0.72333444	0.04824459	1.11237531
	Total PE	26.6032518	26.3904132	26.7419104	25.4894355	26.4230766	26.3296175	0.49071316	1.24967715
C41H73O13F	PI(32:3)	0.00781813	0.00742445	0.00924323	0.00412704	0.01203023	0.00812862	0.00287532	0.49906366
C41H75O13F	PI(32:2)	0.00195453	0	0	0.00206352	0.00185081	0.00117377	0.00107414	1.25435609
C41H77O13F	PI(32:1)	0	0	0	0	0.00185081	0.00037016	0.00082771	3.41137985
C41H79O13F	PI(32:0)	0	0.00433093	0.00051351	0.00670644	0.00508971	0.00332812	0.0029377	2.09584674
C43H75O13F	PI(34:4)	0	0	0.0041081	0.00206352	0.00185081	0.00160449	0.0017094	0.77211171
C43H77O13F	PI(34:3)	0.26093018	0.06867616	0.18435108	0.35028258	0.43910355	0.26066871	0.14366213	0.62648357
C43H79O13F	PI(34:2)	0.94794859	0.2536687	0.60183696	1.2855732	1.63888807	0.9455831	0.54616334	0.52291547
C43H81O13F	PI(34:1)	0.00781813	0.00247482	0	0	0	0.00205859	0.00339334	2.4652328
C45H75O13F	PI(36:6)	0.01563627	0.00371222	0.00718918	0.0051588	0.01295564	0.00893042	0.00513949	0.30588994
C45H77O13F	PI(36:5)	0.0058636	0.00123741	0.00924323	0.01960344	0.02220966	0.01163147	0.00897857	0.39518671
C45H79O13F	PI(36:4)	0.03322706	0.01237408	0.02259456	0.04539745	0.05367335	0.0334533	0.0166869	0.30954554
C45H81O13F	PI(36:3)	0.00390907	0	0.00308108	0.00825408	0.00555242	0.00415933	0.00305094	0.81999119
C45H83O13F	PI(36:2)	0.01563627	0.00371222	0.00924323	0.0154764	0.01850805	0.01251524	0.00597123	0.70215761
C45H85O13F	PI(36:1)	0.0117272	0.00123741	0.0041081	0.00722232	0.01017943	0.00689489	0.00430174	0.41027691
	Total PI	1.31246902	0.3588484	0.85551227	1.7519288	2.22374254	1.30050021	0.73161291	0.54637146
C40H70O10F	PS(34:4)	0	0	0.00225462	0	0	0.00045092	0.0010083	1.58487138
C40H72O10F	PS(34:3)	0.03647155	0.03938884	0.02818276	0.04869781	0.04164634	0.03887746	0.00749221	1.09090881
C40H74O10F	PS(34:2)	0.19093927	0.14261477	0.1544415	0.20045378	0.17369569	0.172429	0.02419742	1.3350969
C40H76O10F	PS(34:1)	0.00429077	0	0	0.00906006	0.00203153	0.00307647	0.00378481	1.28393956
C42H70O10F	PS(36:6)	0	0.00271647	0	0	0	0.00054329	0.00121484	1.99177457
C42H72O10F	PS(36:5)	0.00858154	0.01629883	0.00901848	0.01359009	0.00203153	0.00990409	0.00545413	1.30609811
C42H74O10F	PS(36:4)	0.02574462	0.04346355	0.03381931	0.02718017	0.03859904	0.03376134	0.00750199	1.15121359
C42H76O10F	PS(36:3)	0.00429077	0.01086589	0.00450924	0.00906006	0.00203153	0.0061515	0.00366806	1.50463311
C42H78O10F	PS(36:2)	0.02145385	0.00814942	0.00901848	0.00906006	0.02234681	0.01400572	0.00722283	2.08871444
C42H80O10F	PS(36:1)	0	0	0	0	0	0	0	#DIV/0!
C44H74O10F	PS(38:6)	0	0	0	0.00226501	0	0.000453	0.00101295	1.72398334
C44H76O10F	PS(38:5)	0	0	0	0	0.00203153	0.00040631	0.00090853	0
C44H78O10F	PS(38:4)	0.00429077	0.00271647	0.00450924	0.00226501	0.00203153	0.00316261	0.00115869	0.7064504
C44H80O10F	PS(38:3)	0	0.00271647	0.00225462	0.00679504	0.00609459	0.00357214	0.00282746	1.12458905
C44H82O10F	PS(38:2)	0.04076232	0.03938884	0.02141889	0.04190277	0.03148869	0.0349923	0.00861525	1.16461891
C44H84O10F	PS(38:1)	0	0	0	0	0	0	0	#DIV/0!
C46H82O10F	PS(40:4)	0	0	0	0	0	0	0	#DIV/0!
C46H84O10F	PS(40:3)	0.05578001	0.05704591	0.06087475	0.06115539	0.08329267	0.06362975	0.01123953	1.20441427
C46H86O10F	PS(40:2)	0.43980394	0.41697844	0.43626906	0.63533654	0.54140236	0.49395807	0.0927704	1.27554906

C46H88O10F	PS(40:1)	0	0.0149406	0.00789117	0.00566254	0.01117341	0.00793354	0.00565013	1.51463383
C48H86O10F	PS(42:4)	0	0	0	0	0.00203153	0.00040631	0.00090853	0.87945685
C48H88O10F	PS(42:3)	0.05148924	0.05161297	0.04734703	0.07701049	0.05281974	0.05605589	0.01189544	0.9680333
C48H90O10F	PS(42:2)	0.63503398	0.51612966	0.47121568	0.68176934	0.59523787	0.5798773	0.08592337	1.02458085
C48H92O10F	PS(42:1)	0	0	0	0	0	0	0	#DIV/0!
C50H92O10F	PS(44:3)	0	0	0	0	0	0	0	#DIV/0!
C50H94O10F	PS(44:2)	0	0	0.00225462	0	0	0.00045092	0.0010083	0.83083107
	Total PS	1.51893262	1.36502713	1.29527946	1.83126415	1.60998639	1.52409795	0.21178763	1.17270954
C35H69O8P	PA(32:0)	0.00555419	0.00351634	0.00875549	0.0117278	0.00525943	0.00696265	0.0032666	1.32618225
C37H61O8P	PA(34:6)	0	0	0	0	0	0	0	#DIV/0!
C37H63O8P	PA(34:5)	0.01110838	0.00703268	0	0	0.00525943	0.0046801	0.00476969	0.30897782
C37H65O8P	PA(34:4)	0.04998773	0.09494117	0.17219125	0.15539336	0.12622628	0.11974796	0.04884856	0.15703781
C37H67O8P	PA(34:3)	0.9414355	1.46807187	1.67667581	1.57885518	1.62910796	1.45882926	0.29943276	0.29194545
C37H69O8P	PA(34:2)	3.67965202	5.45208487	6.19742571	6.20254064	6.47304155	5.60094896	1.13927444	0.32070997
C37H71O8P	PA(34:1)	0	0	0	0	0	0	0	#DIV/0!
C39H65O8P	PA(36:6)	0.11386093	0.33229411	0.28747183	0.3474361	0.35895599	0.28800379	0.10106194	0.26206954
C39H67O8P	PA(36:5)	0.85534553	1.32917645	1.54388425	1.47477095	1.55679082	1.3519936	0.29198379	0.17837279
C39H69O8P	PA(36:4)	1.91341905	3.43722216	3.77215575	3.64294812	3.89855133	3.33285928	0.81161046	0.20127052
C39H71O8P	PA(36:3)	0.08608997	0.11779738	0.12695456	0.17445104	0.16172742	0.13340408	0.03539369	0.8814792
C39H73O8P	PA(36:2)	0.12496931	0.1986732	0.22910192	0.20377054	0.20380285	0.19206356	0.03934472	0.46962301
	Total PA	7.78142261	12.4408102	14.0146166	13.7918937	14.4187231	12.4894932	2.73461492	0.27801618
	Total	100	100	100	100	100	100	0	
